# Catalytic direct amidations in *tert*-butyl acetate using B(OCH_2_CF_3_)_3_†
†Electronic supplementary information (ESI) available: Experimental procedures and data for all compounds, along with ^1^H and ^13^C NMR spectra for all amides. See DOI: 10.1039/c9ob01012b


**DOI:** 10.1039/c9ob01012b

**Published:** 2019-06-14

**Authors:** Charlotte E. Coomber, Victor Laserna, Liam T. Martin, Peter D. Smith, Helen C. Hailes, Michael J. Porter, Tom D. Sheppard

**Affiliations:** a Department of Chemistry , University College London , 20 Gordon St , London , WC1H 0AJ , UK . Email: Tom.sheppard@ucl.ac.uk; b Early Chemical Development , Pharmaceutical Sciences , IMED Biotech Unit , AstraZeneca , Macclesfield SK10 2NA , UK

## Abstract

Catalytic direct amidation reactions have been the focus of considerable recent research effort, due to the widespread use of amide formation processes in pharmaceutical synthesis. However, the vast majority of catalytic amidations are performed in non-polar solvents (aromatic hydrocarbons, ethers) which are typically undesirable from a sustainability perspective, and are often poor at solubilising polar carboxylic acid and amine substrates. As a consequence, most catalytic amidation protocols are unsuccessful when applied to polar and/or functionalised substrates of the kind commonly used in medicinal chemistry. In this paper we report a practical and useful catalytic direct amidation reaction using *tert*-butyl acetate as the reaction solvent. The use of an ester solvent offers improvements in terms of safety and sustainability, but also leads to an improved reaction scope with regard to polar substrates and less nucleophilic anilines, both of which are important components of amides used in medicinal chemistry. An amidation reaction was scaled up to 100 mmol and proceeded with excellent yield and efficiency, with a measured process mass intensity of 8.

## Abstract 2

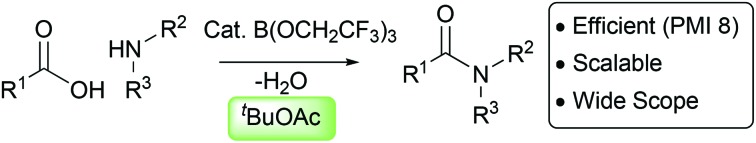
B(OCH_2_CF_3_)_3_-catalysed direct amidations of challenging substrates (polar heteroycles, poorly nucleophilic anilines) work well in ^*t*^BuOAc under Dean–Stark conditions.

## Introduction

**Scheme 1:**
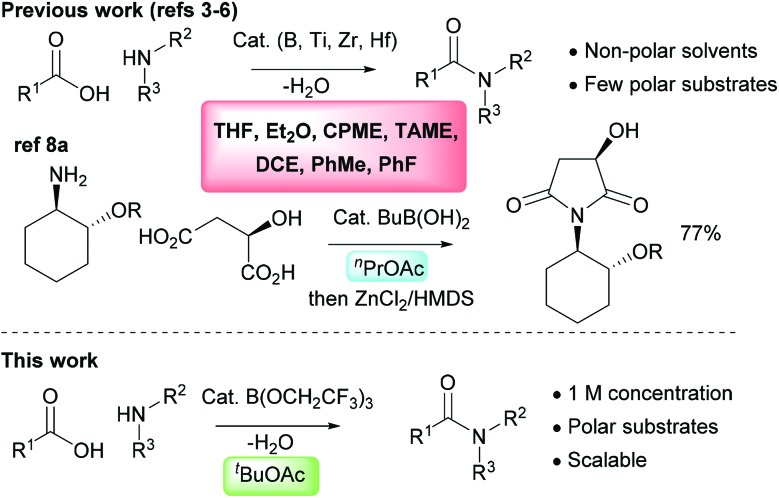
Solvents previously employed in catalytic amidation reactions and our new approach using *tert*-butyl acetate. CPME = cyclopentyl methyl ether; TAME = *tert*-amyl methyl ether; DCE = 1,2-dichloroethane.

The direct amidation reaction between carboxylic acids and amines is one of the most common processes employed in the synthesis of organic molecules in both academic and industrial organisations. It remains a highly inefficient reaction, however, due to the widespread use of stoichiometric activating agents that leads to the generation of high molecular weight by-products as well as increased solvent use during both work-up and purification.^1^,^2^ There has consequently been considerable interest in recent years in the development of catalytic direct amidation reactions which enable amides to be produced from carboxylic acids and amines with a molecule of water as the only stoichiometric by-product. Common catalytic systems include those based around boronic acids,^3^ boric acid derivatives^4^ or boron heterocycles,^5^ as well as salts of the group(IV) metals titanium, zirconium or hafnium.^6^ However, to date these catalytic amidation methods have failed to become widely adopted.^7^ This is partly due to the fact that they cannot often be applied to common amide targets which incorporate polar functional groups such as heterocyclic rings. It is also a consequence of the poor efficiency of many catalytic amidation reactions due to the large quantities of solvents employed both in the reaction itself and in the work-up procedure.^7^ The use of molecular sieves to efficiently remove water from the reaction mixture exacerbates these problems as higher dilution reaction conditions are normally required, along with excess solvent during work-up for washing the molecular sieves to recover the amide product. For larger scale reactions, Dean–Stark water removal is employed as this is considerably more efficient in terms of solvent usage and scalability, and there are a few reports of direct amidation reactions catalysed by boric acid or simple boronic acids being employed on an industrial scale.^8^ The solvents used for catalytic amidation reactions are typically aromatic hydrocarbons (toluene,3a,4a fluorobenzene3*b*), chlorinated solvents (1,2-dichloroethane3*i*) or ethers (Et_2_O,6*d* THF,5a,b CPME,4*e* TAME4g,h), and these are often sub-optimal from a safety and/or sustainability perspective (Scheme 1).^9^ In addition, these relatively non-polar solvents do not effectively solubilise many polar functionalised carboxylic acids or amines. In this paper, we outline a highly effective and scalable method for performing catalytic direct amidation reactions in an ester solvent, and demonstrate its application to challenging substrates including polar heterocycles and poorly nucleophilic anilines.

## Results & discussion

### Background

We have recently reported that B(OCH_2_CF_3_)_3_ can be employed as a very effective catalyst for direct amidation reactions which is applicable to a wide range of substrates.4*g* Remarkably, it can also be used for the catalytic direct amidation of many unprotected amino acids.4*h* The reactions were largely performed in *tert*-amyl methyl ether (TAME) as solvent, although selected reactions involving less nucleophilic but relatively apolar amines were more efficient in toluene. Important advantages of this catalytic system include an extremely wide substrate scope, together with a relatively high reaction concentration (0.5 M) which leads to efficient and scalable reactions. B(OCH_2_CF_3_)_3_ is available commercially, or can be synthesised on a large scale from B_2_O_3_ and CF_3_CH_2_OH.4d,g

**Scheme 2:**
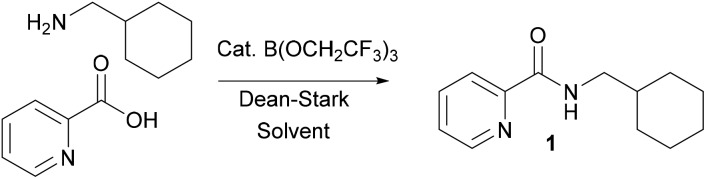
Preparation of amide **1***via* B(OCH_2_CF_3_)_3_-catalysed amidation.

During the course of an ongoing research project on Pd-catalysed direct C–H arylation reactions,^10^ we needed to prepare a series of amides derived from picolinic acid such as **1** (Scheme 2). Initial evaluation of our catalytic amidation method using TAME as a solvent led to a moderate 71% yield of the desired amide **1** with 20 mol% catalyst (Table 1, entry 1).

**Table 1:** Optimisation of reaction solvent and conditions for the synthesis of amide **1**. Reaction conditions: 5 mmol scale, 0.5 M concentration in indicated solvent, 10 mol% B(OCH_2_CF_3_)_3_, reflux, Dean–Stark. 24 h

Entry	Catalyst	Solvent	Bp	Yield^*a*^
1^*b*^	B(OCH_2_CF_3_)_3_	TAME	86 °C	71%
2	B(OCH_2_CF_3_)_3_	EtOAc	77 °C	9%
3^*c*^	B(OCH_2_CF_3_)_3_	^*n*^PrOAc	102 °C	27%
4	B(OCH_2_CF_3_)_3_	^*n*^BuOAc	126 °C	20%
5	B(OCH_2_CF_3_)_3_	^i^PrOAc	89 °C	52%
6	B(OCH_2_CF_3_)_3_	^*t*^BuOAc	97 °C	75%
**7^*c*^**	**B(OCH**_**2**_ **CF**_**3**_ **)**_**3**_	^***t***^ **BuOAc**	**97 °C**	**91%**
8^*c*^	B(OCH_2_CF_3_)_3_	EtCN	97 °C	76%
9^*c*^	B(OCH_2_CF_3_)_3_	^*n*^PrCN	117 °C	64%
10^*d*^	B(OMe)_3_	^*t*^BuOAc	97 °C	8%
11	B(OEt)_3_	^*t*^BuOAc	97 °C	10%
12^*c*^	Ti(O^i^Pr)_4_	^*t*^BuOAc	97 °C	14%
13^*e*^	ArB(OH)_2_	^*t*^BuOAc	97 °C	73%

^*a*^Isolated yields.

^*b*^20 mol% B(OCH_2_CF3)_3_.

^*c*^1 M concentration.

^*d*^0.75 M concentration.

^*e*^Ar = 3,5-bis-(trifluoromethyl)phenyl.

We hypothesised that this was due to the relatively poor solubility of picolinic acid (and the corresponding ammonium salt) in TAME. We therefore decided to explore other reaction solvents which might circumvent these problems. Esters were identified as potentially suitable, as they are typically greener and more sustainable solvents than ethers or aromatic hydrocarbons, and offer a better safety profile (*i.e.* they are typically not prone to peroxide formation).^9^ They are also considerably more polar so should be much more effective at solubilising polar substrates and/or ammonium salts derived from them. Importantly, we had previously observed that stoichiometric amidation mediated by B(OCH_2_CF_3_)_3_ could be carried out in ethyl acetate as a solvent without any significant amidation of the ester being observed.4*e* To date, there is only a single report of a catalytic amidation reaction performed in an ester solvent, using BuB(OH)_2_ as a catalyst in *n*-propyl acetate.8*a* Aside from this, esters have not been explored as solvents for catalytic amidation reactions.

### Reaction optimisation

We selected the synthesis of amide **1** as a test reaction for evaluating a range of ester solvents. Using 10 mol% B(OCH_2_CF_3_)_3_ catalyst, the reaction could be performed in a range of ester solvents under Dean–Stark reaction conditions though the yields were somewhat variable (Table 1, entries 2–7). A very low yield was obtained in ethyl acetate (entry 2), and *n*-propyl acetate (entry 3) or *n*-butyl acetate (entry 4) offered relatively little improvement. Pleasingly, isopropyl acetate (entry 5) and *tert*-butyl acetate (entry 6) gave significantly improved yields with the latter being particularly effective, even though these solvents have lower boiling points than their unbranched isomers. The yield of amide **1** could be further improved in *tert*-butyl acetate by increasing the concentration of the reaction mixture to 1 M, leading to a reduction in the solvent requirements of the process. Finally, the nitrile solvents propionitrile (entry 8) and butyronitrile (entry 9) were also effective for the amidation reaction, potentially offering useful more-polar alternative solvents.^11^ It should be noted that the most effective solvents have relatively low boiling points and form azeotropes with a high proportion of water present, with *tert*-butyl acetate (bp 97 °C; 22 wt% H_2_O in azeotrope) and propionitrile (bp 97 °C; 24 wt% H_2_O in azeotrope) being most effective.^12^ This may reflect a balance between the need for efficient water removal from the reaction and the greater levels of catalyst decomposition which may take place at higher reaction temperatures.‡
‡Analysis of the reaction mixture and Dean–Stark trap by ^19^F NMR indicated that in ^*t*^BuOAc, 49% of the CF_3_CH_2_OR group remained in the reaction mixture after 24 h (*cf.* 78% in TAME4*g*). In contrast, analysis of an amidation performed in ^*n*^BuOAc showed that only 16% of the CF_3_CH_2_OR group remained in the reaction mixture after 24 h. Minor additional signals could be seen in the ^19^F NMR spectrum of the ^*t*^BuOAc reaction, whereas in ^*n*^BuOAc multiple species were present at significant concentration. See ESI† for further details. A selection of other simple amidation catalysts (entries 10–13) were also evaluated in ^*t*^BuOAc, with only an arylboronic acid being effective.

### Reaction scope

**Scheme 3:**
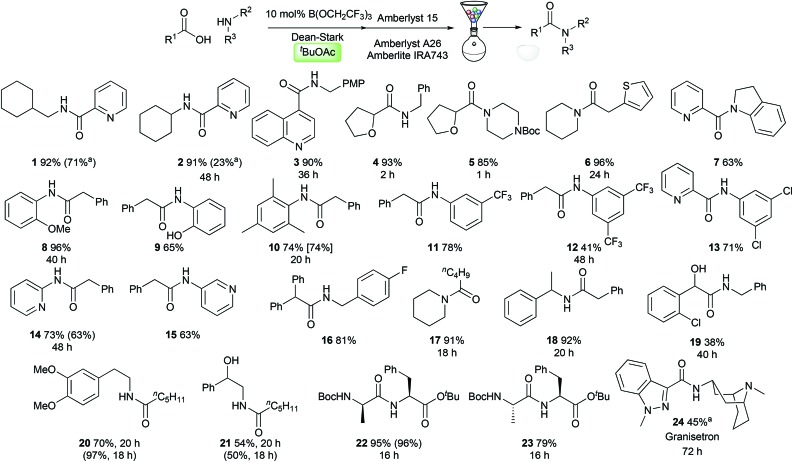
Scope of the catalytic amidation reaction in *tert*-butyl acetate. All reactions were run for 24 h unless otherwise indicated. Yields given in parentheses/brackets are for the synthesis of the same amide in TAME/PhMe respectively. ^a^ 20 mol% B(OCH_2_CF_3_)_3_ used.

With optimised conditions in hand, we evaluated them in the synthesis of a selection of challenging amides **1–24**. We were particularly interested in examining the reactivity of medicinally relevant substrates such as polar heterocycles and poorly nucleophilic amines. Pleasingly, amides could be prepared effectively from heterocyclic carboxylic acids, containing a pyridine (**1–2**, **7**), a quinoline (**3**), a tetrahydrofuran (**4–5**), and a thiophene (**6**). A range of anilines and related derivatives could also be employed including indoline (**7**), electron-rich anilines (**8–10**), electron-deficient anilines (**11–13**) and aminopyridines (**14–15**). Aliphatic amines including simple alkylamines (**1–4**), electron-deficient benzylamines (**16**), secondary amines (**5–6**, **17**), and branched systems (**18**, **22–24**) were also good substrates. The amide **19** derived from 2-chloromandelic acid was obtained in only 38% yield, demonstrating the challenging nature of this particular substrate. Amides derived from hexanoic acid and dimethyldopamine (**20**) or 1-phenylethanolamine (**21**) were synthesised effectively, with the latter reaction demonstrating that the amidation reaction is chemoselective for acylation of the amine over the alcohol. Dipeptide synthesis from Boc-protected d- or l-alanine and l-phenylalanine *tert*-butyl ester proceeded efficiently, giving diastereomeric amides **22**/**23** with no observable epimerisation. Finally, the serotonin 5-HT3 receptor antagonist Granisetron **24** (used commonly as an antiemetic)^13^ could be prepared in 45% yield using only 20 mol% catalyst. Notably, in several cases amides were prepared from reactants where both the carboxylic acid and the amine can be considered as challenging substrates for catalytic amidation (**5**, **7**, **13**, **22–24**).§
§We have previously noted that 2-aminopyridine (amide **14**) shows low reactivity in stoichiometric B(OCH_2_CF_3_)_3_ amidation reactions.4*e* Others have recently noted that sterically encumbered (amides **8–10**) or electron-deficient (amides **11–13**) anilines were particularly challenging substrates for catalytic amidation reactions.5*a* As can be seen in Scheme 3, with the exception of amide **20**, *tert*-butyl acetate offers comparable or improved amide yields over amide syntheses previously performed in TAME or toluene (**1**, **2**, **10**, **14**, **21**). In most cases, the amides could be purified using a solid-phase work-up procedure employing scavenger resins to remove unreacted amine (Amberlyst 15), carboxylic acid (Amberlyst A-26) and boron compounds (Amberlite IRA743), with no requirement for aqueous work-up or chromatography.4*d*

### Multigram scale reaction

**Scheme 4:**
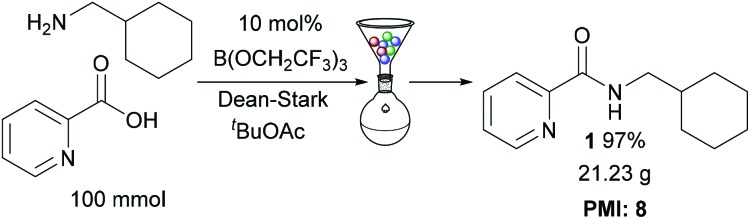
Large scale synthesis of amide **1**.

To demonstrate the efficiency of our protocol, amide **1** was prepared on a 100 mmol scale (Scheme 4), using the solid-phase work-up procedure to purify the amide. This gave 21.23 g of amide **1** in 97% overall yield. The process mass intensity^14^ for the reaction was calculated to be 8, showing that this catalytic amidation method is extremely competitive for use in large scale preparations (*e.g.* a typical PMI for stoichiometric amidation reactions used in the synthesis of pharmaceutical intermediates is ∼43 (ref. 7*a*)).

## Conclusions

We have demonstrated that *tert*-butyl acetate is a very effective solvent for B(OCH_2_CF_3_)_3_-catalysed direct amidation reactions. These conditions were found to be particularly effective for challenging pharmaceutically relevant substrates including carboxylic acids containing polar heterocycles, as well as electron-deficient anilines and aminopyridines. The reactions can be run at 1 M concentration, and the amides can typically be purified using a simple resin-based filtration process which has low solvent requirements. A 100 mmol synthesis of an amide gave a 97% yield of product with process mass intensity of **8**. *tert*-Butyl acetate is a readily available low-cost solvent, produced industrially from acetic acid and isobutylene, which has a good safety profile^9^ making it potentially suitable for widespread use in catalytic direct amidation reactions.

## Experimental section

### General amidation procedure

A suspension of carboxylic acid (5 mmol, 1 equiv.), amine (5 mmol, 1 equiv.) and B(OCH_2_CF_3_)_3_ (108 μL, 0.5 mmol, 10 mol%) in ^*t*^BuOAc (5 mL, 1 M) with a Dean–Stark trap (side arm filled with ^*t*^BuOAc) was heated to reflux. An air condenser was fitted and the reaction mixture heated for 1–48 h (see Scheme 3). The reaction was cooled to room temperature and water (0.5 mL), dimethyl carbonate (5 mL), Amberlite IRA-743 (0.25 g), Amberlyst A15 (0.5 g) and A-26(OH) (0.5 g) resins were added and the resulting suspension was stirred for 30 min. Anhydrous magnesium sulfate (∼0.5 g) was added, the mixture filtered, and the resins/solids washed with ethyl acetate (2 × 5 mL). The combined filtrates were concentrated *in vacuo* to yield the pure amide.

## Further experimental details

Characterisation data for all amides **1–24**, together with ^1^H and ^13^C spectra can be found in the ESI.†

## Conflicts of interest

There are no conflicts to declare.

## Supplementary Material

Supplementary information
