# Supplementary material for: Catalytic direct amidations in tert-butyl acetate using B(OCH2CF3)3
Source: Org Biomol Chem. 2019 Jun 14;17(26):6465–9. doi: 10.1039/c9ob01012b (PMC6724682; doi:10.1039/c9ob01012b)

Electronic Supplementary Material (ESI) for Organic & Biomolecular Chemistry.
This journal is © The Royal Society of Chemistry 2019

Catalytic Direct Amidations in *tert*-Butyl Acetate using $B(OCH_2CF_3)_3$

Charlotte E. Coomber, Victor Laserna, Liam T. Martin, Peter D. Smith, Helen C. Hailes, Michael J. Porter, Tom D. Sheppard*

Department of Chemistry, 20 Gordon Street, University College London, London, UK

Email tom.sheppard@ucl.ac.uk

Contents

1. General information P2
2. Solvent and catalyst screening/stability study P3
3. General procedure for catalytic amidation P7
4. Large scale amidation and PMI calculations P7
5. Spectroscopic data P8
6. ^{1}H and ^{13}C NMR spectra P20

1. General Methods

All reagents and solvents were purchased and used as supplied unless otherwise stated. All reactions were carried out at atmospheric pressure with stirring and under air atmosphere unless otherwise indicated. Piperidine was distilled before use. Preparation of Tris(2,2,2-trifluoroethyl)borate was performed according to literature procedure (*Sci. Adv.*, **2017**, 3, e1701028). All resins were pre-washed with EtOAc, Et_2O and CH_2Cl_2 and dried in vacuo prior to use. *In vacuo* is used to describe evaporation of solvent by Büchi rotary evaporator between 17 ºC and 50 ºC at a pressure of ~ 10 mmHg. All reactions were monitored by TLC or ^{1}H NMR. TLC plates used were pre-coated with silica gel 60 F254 on aluminium (Merck KGaA). The spotted TLCs were visualised by UV light (254 nm or 365 nm) or chemically stained ($KMnO_4$, or Ninhydrin). ^{1}H NMR and ^{13}C NMR spectra were recorded at 400, 600 or 700 MHz (for ^{1}H) and 100, 125 or 175 MHz (for ^{13}C) on a Bruker AMX400, AMX600 or NEO700 at ambient temperature, unless otherwise indicated. Deuterated solvents for NMR detection used were $CDCl_3$, MeOD-d4 or DMSO-d6 as stated in the spectrum. Peaks are assigned as singlet (s), doublet (d), triplet (t), quartet (q), quintet (qn) or multiplet (m). All shifts are reported in parts per million (ppm) and compared against residual solvent signals: $CDCl_3$ (δ = 7.26 ppm, s), DMSO (δ = 2.50 ppm, qn) or MeOD (δ = 3.31, qn) as the internal standard. Coupling constants (J) are quoted in Hertz (Hz) to one decimal place. Mass spectrometry was performed on VG70 SE (ES+, CI, ES- modes). Infra-red spectra were obtained using a Perkin-Elmer Spectrum 100 FTIR Spectrometer operating in ATR mode, all frequencies given in reciprocal centimetres (cm^{-1}). Melting points were measured with a Gallenkamp heating block and are uncorrected.

2. Optimisation of conditions and catalysts

A suspension of cyclohexanmethylamine (652 µL, 5.0 mmol, 1 eq), 2-picolinic acid (615 mg, 5.0 mmol, 1 eq) and $B(OCH_2CF_3)_3$ in solvent (5-10 mL as below) with a Dean-Stark apparatus (side arm filled with the given solvent) was heated to reflux for 24 hours. The reaction mixture was cooled to room temperature and water (0.5 mL), dimethyl carbonate (5 mL) Amberlite IRA-743 (0.25 g) and A-26(OH) (0.5 g) resins were added and the resulting suspension was stirred for 30 min. $MgSO_4$ (~0.5 g) was added and the mixture filtered and the resins washed with EtOAc (2 × 5 mL). The combined filtrates were concentrated *in vacuo* to yield the amide. In cases where the reaction had not gone to full conversion the product was further purified by flash column chromatography (20% EtOAc in petrol).

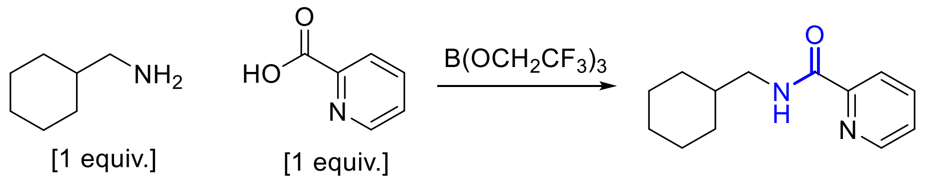

**Entry**	**Solvent**	**Concentration [M]**	**$B(OCH_2CF_3)_3$ mol%**	**Yield (isolated)**
1	*t*BuOAc	1	20	92
2	*t*BuOAc	0.5	10	84
3	*i*PrOAc	0.5	10	52
4	EtOAc	0.5	10	9
5	*n*BuOAc	0.5	10	20
6	*t*BuOAc	0.5	10	75
7	***t*BuOAc**	**1**	**10**	**91**
8	*t*BuOAc	0.5	10	59
9	*n*PrOAc	1	10	27
10	*n*PrCN	1	10	64
11	EtCN	1	10	76
12	TAME	1	20	71

Catalyst screen

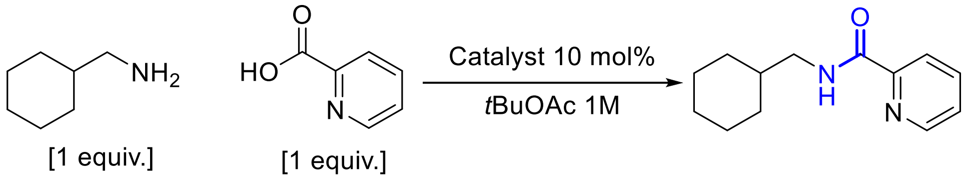

Entry	Catalyst	Yield
1	$B(OCH_2CF_3)_3$	91
2	$B(OMe)_3$	8
3	$B(OEt)_3$	10
4	$Ti(O^iPr)_4$	14
5	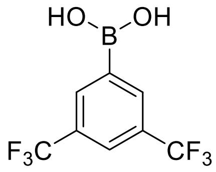	73
6[a]	$B(OMe)_3$	9

[a] 0.75 M

2. Catalyst Stability Study

A suspension of cyclohexanmethylamine (652 µL, 5.0 mmol, 1 eq), 2-picolinic acid (615 mg, 5.0 mmol, 1 eq) and $B(OCH_2CF_3)_3$ (107 µl, 0.5 mmol) in solvent (5 mL of either ^{t}BuOAc or ^{n}BuOAc) with a Dean-Stark apparatus (side arm filled with the given solvent) was heated to reflux for 24 hours then allowed to cool to room temperature. The contents of the Dean-Stark trap were transferred to a flask and fluorobenzene (96 mg, 1 mmol) was added as an internal standard. A sample from this mixture was removed, diluted with CDCl3 and analysed by ^{19}F NMR. Fluorobenzene (96 mg, 1 mmol) was also added to the reaction flask and a sample from this mixture was also analysed by ^{19}F NMR.

^{t}BuOAc

The reaction flask contained 49% of the total quantity of CF_3CH_2OH (green/blue).

The Dean-Stark trap contained 51% (red).

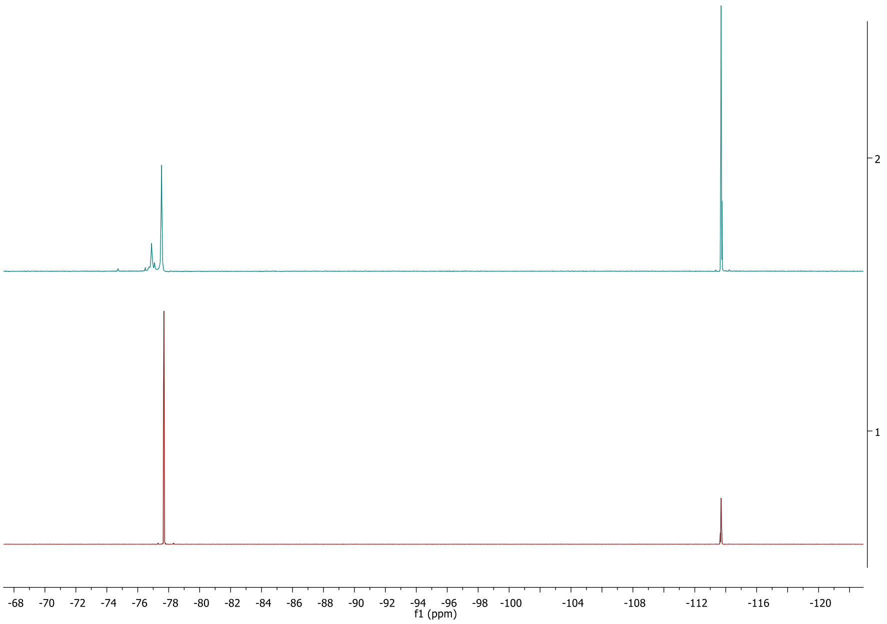

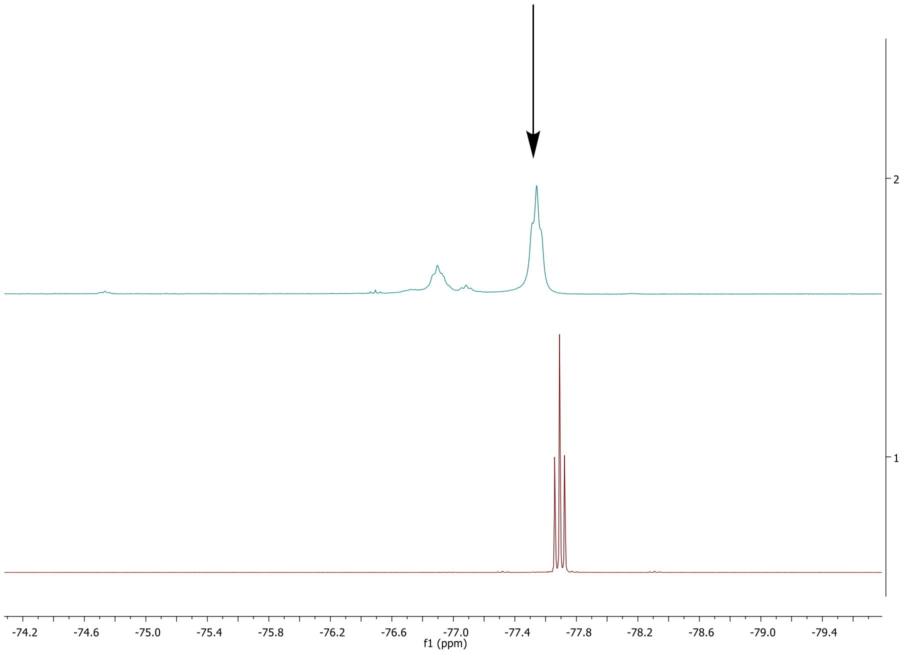

The peak indicated with the arrow is the same signal observed in a similar reaction in TAME where 78% of the CF_3CH_2OH remained in the reaction flask after 24 h (*Sci. Adv.* **2017**, *3*, e1701028).

^{n}BuOAc

The reaction flask contained 16% of the total quantity of CF_3CH_2OH (green/blue).

The Dean-Stark trap contained 84% (red).

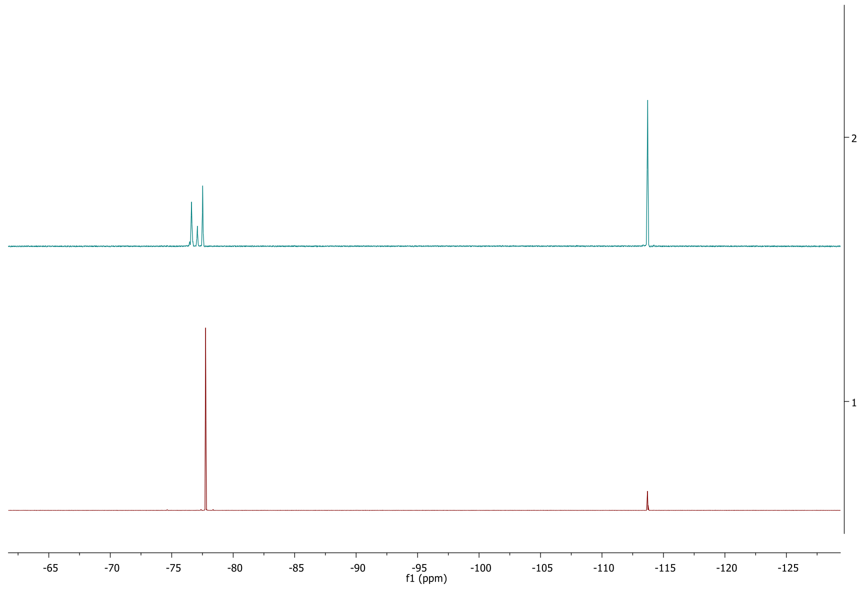

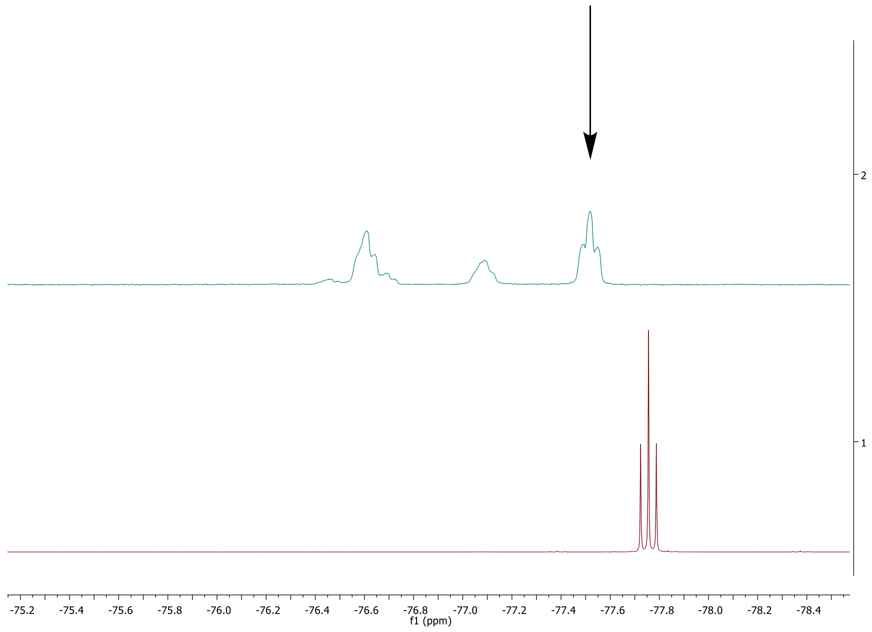

The peak indicated with the arrow is the same signal observed in a similar reaction in TAME where 78% of the CF_3CH_2OH remained in the reaction flask after 24 h (*Sci. Adv.* **2017**, *3*, e1701028).

3. General Procedure for catalytic amidation

A suspension of carboxylic acid (5 mmol, 1 equiv.), amine (5 mmol, 1 equiv.) and $B(OCH_2CF_3)_3$ (108 µL, 0.5 mmol, 10 mol%) in *t*BuOAc (5 mL, 1 M) with a Dean-Stark apparatus (side arm filled with *t*BuOAc) was heated to reflux. An air condenser was fitted and the reaction mixture heated for 1 – 48 hours. Upon completion, the reaction was cooled to room temperature and water (0.5 mL), dimethyl carbonate (5 mL) Amberlite IRA-743 (0.25 g), Amberlyst A15 (0.5 g) and A-26(OH) (0.5 g) resins were added and the resulting suspension was stirred for 30 min. $MgSO_4$ (~0.5 g) was added and the mixture filtered and the resins washed with EtOAc (2 × 5 mL). The combined filtrates were concentrated *in vacuo* to yield the pure amide.

4. Large scale amidation and PMI calculations

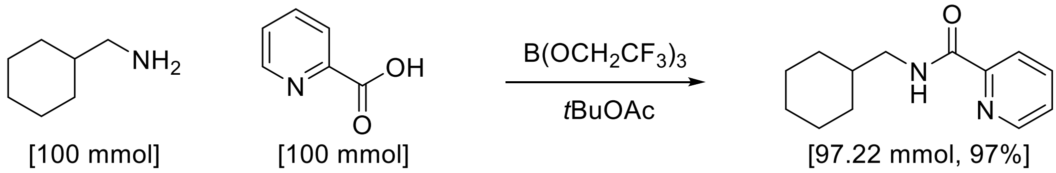

A suspension of 2-picolinic acid (12.31 g, 100 mmol, 1 equiv.), cyclohexanemethylamine (13.01 mL, 100 mmol, 1 equiv.) and $B(OCH_2CF_3)_3$ (2.15 mL,10 mmol, 10 mol %) in *t*BuOAc (100 mL, 1 M) with a Dean-Stark (side arm filled with *t*BuOAc) was heated to reflux. An air condenser was fitted and the reaction mixture heated for 24 hours. Upon completion, the reaction was cooled to room temperature and water (10 mL), Amberlite IRA-743 (2.5 g) and A-26(OH) (10 g) resins were added and the resulting suspension was stirred for 30 min. $MgSO_4$ (10 g) was added and the mixture filtered and the resins washed with EtOAc (3 × 10 mL). The combined filtrates were concentrated *in vacuo* to yield the amide as a white solid (21.23 g, 97.22 mmol, 97%).

$$\textbf{Process Mass Intensity} = \frac{\textbf{total mass in a process or process step}}{\textbf{mass of product}}$$

**Conditions**	**Input Reaction**	**Input Workup**	**Yield**
Solvent (1 M)	*t*BuOAc (86.6 g)	Resins (12.5 g)	
Catalyst	$B(OCH_2CF_3)_3$ (3.079 g)	H_2O (10 g)	97%
Acid (1 equiv.)	2-Picolinic acid (12.311 g)	EtOAc (27.06 g)	21.23 g
Amine (1 equiv.)	Cyclohexanemethylamine (11.320 g)	$MgSO_4$ (10 g)	
24 h, 98 °C	*Total = 113.31 g*	*Total = 59.56 g*	

Reaction input+workup/product output

(113.31+59.56)/21.23 **= PMI = 8**

5. Spectroscopic data

N-(Cyclohexylmethyl)picolinamide 1

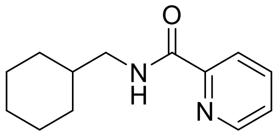

Reaction performed on a 5 mmol or 100 mmol scale for 24 h. Purified using the standard resin work up, but without the use of Amberlyst A-15.

White solid; 5 mmol 92%, 100 mmol scale 97%; **M.p** 65 - 67 °C; **v_{max}** (solid/cm^{-1}) 3354 (NH), 2916 (CH), 2847 (CH), 1657 (CO), 1525 (CC); **^{1}H NMR** (400 MHz, $CDCl_3$) δ 8.57 – 8.51 (m, 1H, Ar*H*), 8.20 (d, *J* = 7.8 Hz, 1H, Ar*H*), 8.12 (s, 1H, N*H*), 7.84 (td, *J* = 7.7, 1.7 Hz, 1H, Ar*H*), 7.44 – 7.38 (m, 1H, Ar*H*), 3.32 (t, *J* = 6.6 Hz, 2H, NHCH_2), 1.80 (m, 2H, CHCH_2), 1.76 – 1.69 (m, 2H, $CHCH_2CH_2$), 1.67 – 1.55 (m, 2H, $CHCH_2CH_2CH_2$ and C*H*), 1.31 – 1.11 (m, 3H, $CHCH_2CH_2$ and $CHCH_2CH_2CH_2$), 1.01 (qd, *J* = 12.1, 2.9 Hz, 2H, $NHCH_2CHCH_2$); **^{13}C NMR** (176 MHz, $CDCl_3$) *δ* 164.4 (C), 150.2 (C), 148.1 (CH), 137.4 (CH), 126.1 (CH), 122.3 (CH), 45.8 (CH_2), 38.3 (CH), 31.0 (CH_2), 26.5 (CH_2), 26.0 (CH_2).
Yield in TAME using 20 mol% $B(OCH_2CF_3)_3$ 71%

[Data in accordance with the literature *Org. Lett*. **2017**, 19, 4880−4883]

N-Cyclohexylpicolinamide 2

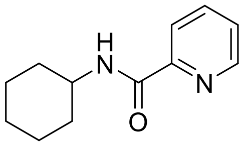

Reaction performed on a 5 mmol scale for 48 h. Purified using the standard resin work up, but without the use of Amberlyst A-15.

White solid, 91%; **M.p** 54 – 56 °C; **v_{max}** (solid/cm^{-1}) 3379, 2935, 2857, 1666; **^{1}H NMR** (400 MHz, $CDCl_3$) *δ* 8.52 (ddd, *J* = 4.8, 1.7, 0.9 Hz, 1H, Ar*H*), 8.18 (dt, *J* = 7.8, 1.1 Hz, 1H, Ar*H*), 7.93 (s, 1H, N*H*), 7.82 (td, *J* = 7.7, 1.7 Hz, 1H, Ar*H*), 7.39 (ddd, *J* = 7.6, 4.8, 1.2 Hz, 1H, Ar*H*),

4.03 – 3.89 (m, 1H, NHC*H*), 2.05 – 1.95 (m, 2H, NHCHC*H*$_2$), 1.80 – 1.70 (m, 2H, NHCHCH$_2$C*H*$_2$), 1.68 – 1.59 (m, 1H, CHCH$_2$CH$_2$C*H*$_2$), 1.48 – 1.16 (m, 5H, 3 × C*H*$_2$); **^{13}C NMR** (176 MHz, $CDCl_3$) *δ* 163.4 (C), 150.4 (C), 148.1 (CH), 137.4 (CH), 126.1 (CH), 122.3 (CH), 48.3 (CH), 33.2 (CH_2), 25.7 (CH_2), 25.0 (CH_2).

The yield in TAME using 20 mol% $B(OCH_2CF_3)_3$ was 23%.

[Data in accordance with the literature *J. Org. Chem*. **2013**, 19, 9689 – 9714]

***N*-(4-Methoxybenzyl)quinoline-4-carboxamide 3**

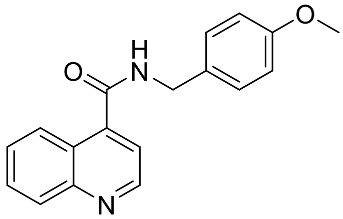

Reaction performed on a 2.5 mmol scale for 36 h. Purified using the standard resin work-up procedure, but without the use of Amberlyst A-15. Further purified by recrystallisation from isopropyl alcohol.

Off white solid, 90%; **M.p** 123 – 124 °C; **ν_{max}** (solid/cm^{-1}) 3273 (NH), 2929 (CH), 2834 (CH), 1634 (CO), 1510 (CC); **^{1}H NMR** (700 MHz, $CDCl_3$) *δ* 8.81 (d, *J* = 4.3 Hz, 1H, Ar*H*), 8.19 (d, *J* = 8.4 Hz, 1H, Ar*H*), 8.06 (d, *J* = 8.4 Hz, 1H, Ar*H*), 7.75 – 7.69 (m, 1H, Ar*H*), 7.56 (t, *J* = 7.6 Hz, 1H, Ar*H*), 7.35 (d, *J* = 4.3 Hz, 1H, Ar*H*), 7.29 (d, *J* = 8.6 Hz, 2H, Ar*H*), 6.90 – 6.86 (m, 2H, Ar*H*), 6.59 (br s, 1H, N*H*) 4.62 (d, *J* = 5.7 Hz, 2H, C*H*$_2$), 3.80 (s, 3H, C*H*$_3$); **^{13}C NMR** (176 MHz, $CDCl_3$) *δ* 167.2 (C), 159.4 (C), 149.9 (CH), 148.7(C), 142.0 (C), 130.1 (CH), 129.9 (CH), 129.8 (C), 129.5 (CH), 127.8 (CH), 125.4 (CH), 124.6 (C), 118.5 (CH), 114.4 (CH), 55.5 (CH_3), 43.8 (CH_2); **HRMS** (ESI) Found: 293.1286, $C_{18}H_{16}N_2O_2$+H ([M+H]$^+$) requires 293.1285.

***N*-Benzyltetrahydrofuran-2-carboxamide 4**

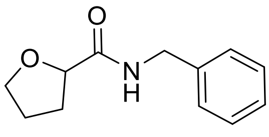

Reaction performed on a 5 mmol scale for 2 h. Purified using the standard resin work up.

Yellow oil, 93%; **ν_{max}** (film/cm^{-1}) 3328 (NH), 2951 (CH), 2875 (CH), 1654 (CO), 1520 (CC); **^{1}H NMR** (700 MHz, $CDCl_3$) *δ* 7.31 (m, 2H, Ar*H*), 7.28 – 7.17 (m, 3H, Ar*H*), 7.15 – 6.91 (s, 1H, N*H*), 4.50 – 4.32 (m, 3H, NHC*H*$_2$, C*H*), 3.93 – 3.79 (m, 2H, OC*H*$_2$), 2.33 – 2.24 (m, 1H, CHC*H*$_2$),

2.11 – 2.03 (m, 1H, CHC*H*$_2$), 1.94 – 1.80 (m, 2H, OCH$_2$C*H*$_2$); **^{13}C NMR** (176 MHz, $CDCl_3$) *δ* 173.3 (C), 138.3 (C), 128.8 (CH), 127.8 (CH), 127.6 (CH), 78.6 (CH), 69.5 (CH_2), 43.0 (CH_2), 30.4 (CH_2), 25.7 (CH_2).

[Data in accordance with the literature *Chem. Commun.*, **2014**, 50, 7017 – 7019]

***tert*-Butyl 4-(tetrahydrofuran-2-carbonyl)piperazine-1-carboxylate 5**

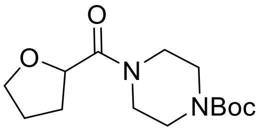

Reaction performed on a 5 mmol scale for 1 h. Purified using the standard resin work up.

White solid, 85%; **M.p** 55 – 58 °C; $\mathbf{v_{max}}$ (solid/cm^{-1}) 2974 (CH), 2868 (CH), 1683 (CO), 1644 (CO); **^{1}H NMR** (700 MHz, $CDCl_3$) *δ* 4.44 (dd, *J* = 7.4, 5.8 Hz, 1H, OC*H*), 3.75 (dd, *J* = 14.7, 7.0 Hz, 1H, OC*H*$_2$), 3.67 (dd, *J* = 13.8, 7.6 Hz, 1H, OC*H*$_2$), 3.52 (m, 2H, NC*H*$_2$), 3.37 – 3.27 (m, 4H, NC*H*$_2$), 3.26 – 3.21 (m, 1H, NC*H*$_2$), 3.20 – 3.14 (m, 1H, NC*H*$_2$), 2.13 (dq, *J* = 12.2, 6.2 Hz, 1H, OCHC*H*$_2$), 1.90 – 1.79 (m, 2H, OCHC*H*$_2$, OCH$_2$C*H*$_2$), 1.77 – 1.71 (m, 1H, OCH$_2$C*H*$_2$), 1.29 (m, 9H, C(C*H*$_3$)$_3$); **^{13}C NMR** (176 MHz, $CDCl_3$) *δ* 170.0 (C), 154.5 (C), 80.1 (C), 75.9 (CH), 69.0 (CH_2), 45.3 (CH_2), 43.6 (br, CH_2), 41.9 (CH_2), 28.3 (CH_2), 25.7 (CH_3); **HRMS** (ESI) Found: 285.1810, $C_{14}H_{24}N_2O_4$+H ([M+H]$^+$) requires 285.1809.

1-(Piperidin-1-yl)-2-(thiophen-2-yl)ethan-1-one 6

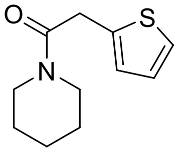

Reaction performed on a 5 mmol scale for 24 h. Purified using the standard resin work up.

Yellow oil, 96%. $\mathbf{v_{max}}$ (film/cm^{-1}) 2932 (CH), 2852 (CH), 1630 (CO); **^{1}H NMR** (700 MHz, $CDCl_3$) *δ* 7.14 (s, 1H, Ar*H*), 6.90 (d, *J* = 2.9 Hz, 1H, Ar*H*), 6.85 (s, 1H, Ar*H*), 3.86 (br s, 2H, COC*H*$_2$), 3.53 (br s, 2H, NC*H*$_2$) 3.40 (br s, 2H, NC*H*$_2$), 1.57 (br s, 2H, NCH$_2$CH$_2$C*H*$_2$), 1.49 (br s, 2H, NCH$_2$C*H*$_2$), 1.41 (br s, 2H, NCH$_2$C*H*$_2$); **^{13}C NMR** (176 MHz, $CDCl_3$) *δ* 168.3 (C), 137.1 (C), 126.9 (CH), 126.0 (CH), 124.7 (CH), 47.5 (CH_2), 43.2 (CH_2), 36.3 (CH_2), 26.4 (CH_2), 25.5 (CH_2), 24.5, (CH_2).

[Data in accordance with the literature *Chem. Commun.*, **2017**, 53, 9159-9162]

Indolin-1-yl(pyridin-2-yl)methanone 7

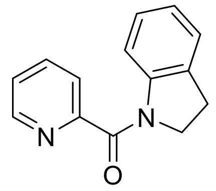

Reaction performed on a 5 mmol scale for 24 h. Purified by flash column chromatography (50% EtOAc in petrol).

White solid, 63%; **M.p** 102 – 103 °C; **v_{max}** (film/cm^{-1}) 3049 (CH), 1630 (CO), 1587, 1480, 1434, 1078, 747. **^{1}H NMR** (600 MHz, $CDCl_3$) δ 8.62 (d, *J* = 4.6 Hz, 1H, Ar*H*), 8.31 (d, *J* = 7.9 Hz, 1H, Ar*H*), 7.85 (m, 3H, Ar*H*), 7.50 – 7.36 (m, 1H, Ar*H*), 7.22 (d, *J* = 7.4 Hz, 1H, Ar*H*), 7.07 (t, *J* = 7.2 Hz, 1H, Ar*H*), 4.35 (t, *J* = 8.2 Hz, 2H, NCH_2), 3.15 (t, *J* = 8.3 Hz, 2H, CH_2). **^{13}C NMR** (176 MHz, $CDCl_3$) δ 166.2 (C), 154.7 (C), 148.1 (CH), 143.41 (C), 137.1 (CH), 132.3 (C), 127.5 (CH), 125.1 (CH), 124.7 (CH), 124.4 (CH), 124.2 (CH), 118.0 (CH), 50.6 (CH_2), 28.8 (CH_2); **HRMS** Found 225.1021 $[C_{14}H_{12}N_2O+H]^+$ requires 225.1022.

***N*-(2-Methoxyphenyl)-2-phenylacetamide 8**

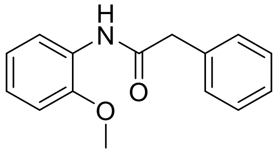

Reaction performed on a 5 mmol scale for 40 h. Purified using the standard resin work up.

White Solid, 96%; **Mp** 81 – 83 °C; **v_{max}** (solid/cm^{-1}) 3259 (NH), 2932 (CH), 2839 (CH), 1643 (CO), 1527 (CC); **^{1}H NMR** (700 MHz, $CDCl_3$) δ 8.36 (dd, *J* = 8.0, 1.3 Hz, 1H, Ar*H*), 7.89 – 7.71 (m, 1H, N*H*), 7.43 – 7.37 (m, 2H, Ar*H*), 7.37 – 7.29 (m, 3H, Ar*H*), 7.01 (td, *J* = 7.9, 1.4 Hz, 1H, Ar*H*), 6.96 – 6.91 (m, 1H, Ar*H*), 6.80 (d, *J* = 8.1 Hz, 1H, Ar*H*), 3.78 (s, 2H, CH_2), 3.69 (s, 3H, CH_3); **^{13}C NMR** (176 MHz, $CDCl_3$) δ 169.1 (C), 148.0 (C), 134.8 (C), 129.7 (C), 129.2 (CH), 127.7 (CH), 127.6 (CH), 123.9 (CH), 121.2 (CH), 119.7 (CH), 110.1 (CH), 55.8 (CH_3), 45.3 (CH_2).

[Data in accordance with the literature *J. Org. Chem*. **2013**, 78, 4512 – 4523]

***N*-(2-Hydroxyphenyl)-2-phenylacetamide 9**

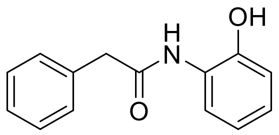

Reaction performed on a 5 mmol scale for 24 h. Purified using the standard resin work up.

Yellow oil, 65%; **ν_{max}** (film/cm^{-1}) 3086 (NH), 3060 (OH), 1613 (CO), 1567, 1453, 1239, 1138, 840 and 761. **^{1}H NMR** (400 MHz, $CDCl_3$) *δ* 7.73 – 7.67 (m, 1H, Ar*H*), 7.49 – 7.44 (m, 1H, Ar*H*), 7.38 (m, 4H, Ar*H*), 7.30 (m, 3H, Ar*H*), 4.28 (s, 2H, C*H*$_2$); **^{13}C NMR** (176 MHz, $CDCl_3$) *δ* 165.3 (CO), 151.2 (C), 141.5 (C), 134.9 (CH), 129.1 (C), 128.9 (CH), 127.4 (CH), 124.8 (CH), 124.3 (CH), 119.9 (CH), 110.6 (CH), 35.4 (CH_2). **HRMS** Found 210.0911 [$C_{14}H_{13}NO_2$-H_2O+H]$^+$ requires 210.0913.
[OH and NH protons are not visible by ^{1}H NMR]

***N*-Mesityl-2-phenylacetamide 10**

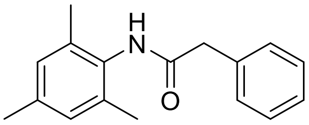

Reaction performed on a 5 mmol scale for 20 h. Purified by flash column chromatography (20-60% EtOAc in petrol).

White solid, 72%; **M.p** 99-102 °C; **ν_{max}** (solid/cm^{-1}) 3250 (NH), 2916 (CH), 1654 (CO), 1517 (CC); **^{1}H NMR** (400 MHz, DMSO) *δ* 9.35 (s, 1H, N*H*), 7.38 – 7.30 (m, 4H, Ar*H*), 7.27 – 7.24 (m, 1H, Ar*H*), 6.84 (s, 2H, Ar*H*), 3.61 (s, 2H, C*H*$_2$), 2.20 (s, 3H, C*H*$_3$), 2.02 (s, 6H, 2 × C*H*$_3$); **^{13}C NMR** (101 MHz, DMSO) *δ* 168.8 (C), 136.5 (C), 135.3 (C), 134.9 (CH), 132.5 (C), 129.0 (CH), 128.3 (CH), 128.2 (CH), 126.5 (CH), 42.6 (CH_2), 20.5 (CH_3), 18.0 (CH_3).

[Data in accordance with the literature *Adv. Synth. Catal*, **2015**, 357, 3273 – 3283]

2-Phenyl-*N*-(3-(trifluoromethyl)phenyl)acetamide 11

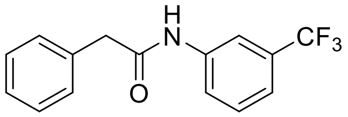

Reaction performed on a 5 mmol scale for 24 h. Purified by flash column chromatography (20% EtOAc in petrol).

White solid, 78%; **M.p** 83 – 84 °C; **v$_{max}$** (film/cm^{-1}) 3268 (NH), 1657 (CO), 1528, 1442, 1332, 1160, 1114, 693; **^{1}H NMR** (600 MHz, $CDCl_3$) δ 7.69 (s, 1H, Ar*H*), 7.63 (d, *J* = 8.1 Hz, 1H, Ar*H*), 7.46 – 7.36 (br s, 1H, N*H*), 7.34 (m, 7H, Ar*H*), 3.76 (s, 2H, C*H*$_2$); **^{13}C NMR** (101 MHz, $CDCl_3$) δ 169.3 (C), 138.1 (C), 134.0 (C), 131.4 (q, $J_{C\text{-}F}$ = 32.5 Hz, C-CF_3), 129.5 (CH), 129.4 (CH), 129.2 (CH), 127.9 (CH), 123.9 (q, $J_{C\text{-}F}$ = 272.5 Hz, CF_3), 123.3 (CH), 121.1 (CH), 116.5 (CH), 44.8 (CH_2); **HRMS** Found 280.0944 [$C_{15}H_{12}F_3NO$+H]$^+$ requires 280.0949.

N-(3,5-bis(Trifluoromethyl)phenyl)-2-phenylacetamide 12

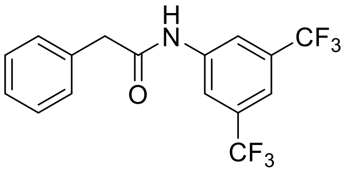

Reaction performed on a 5 mmol scale for 48 h. Purified by flash column chromatography (20% EtOAc in petrol).

White solid, 41%; **M.p** 106 – 107 °C; **v$_{max}$** (film/cm^{-1}) 3288 (NH), 1673 (CO), 1569, 1471, 1382, 1277, 1125, 881, 680; **^{1}H NMR** (400 MHz, $CDCl_3$) δ 7.93 (s, 2H, Ar*H*), 7.58 (s, 1H, Ar*H*), 7.46 – 7.38 (m, 3H, Ar*H*), 7.37-7.34 (m, 2H, Ar*H*), 7.34 (br s, 1H, NH), 3.79 (s, 2H, C*H*$_2$). **^{13}C NMR** (101 MHz, $CDCl_3$) δ 169.5 (C), 139.0 (C), 133.5 (C), 132.4 (q, $J_{C\text{-}F}$ = 32.1Hz, C-CF_3), 129.6 (CH), 129.5 (CH) 128.2 (CH), 123.1 (q, $J_{C\text{-}F}$ = 272.8Hz, CF_3), 119.5 (CH), 117.7 (CH), 44.8 (CH_2); **HRMS** Found 348.0808 [$C_{16}H_{11}F_6NO$+H]$^+$ requires 348.0818.

N-(3,5-Dichlorophenyl)picolinamide 13

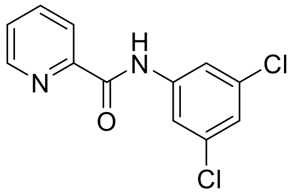

Reaction performed on a 5 mmol scale for 24 h. Purified by flash column chromatography (20 % EtOAc in petrol).

White solid, 71%; **M.p** 169 – 171 °C; **v$_{max}$** (film/cm^{-1}) 3319 (N-H), 1691 (C=O), 1579, 1528, 1441, 1404, 1366, 1279, 1111, 841, 665; **^{1}H NMR** (600 MHz, $CDCl_3$) δ 10.09 (s, 1H, N*H*), 8.61 (ddd, *J* = 4.7, 1.6, 0.9 Hz, 1H, Ar*H*), 8.28 (dt, *J* = 7.8, 1.0 Hz, 1H, Ar*H*), 7.93 (td, *J* = 7.7, 1.5 Hz, 1H, Ar*H*), 7.76 (d, *J* = 1.8 Hz, 2H, Ar*H*), 7.57 – 7.51 (m, 1H, Ar*H*), 7.14 (t, *J* = 1.8 Hz, 1H, Ar*H*); **^{13}C NMR** (176 MHz, $CDCl_3$) δ 162.2 (C), 149.1 (C), 148.2 (CH), 139.6 (C), 138.0 (CH),

135.4 (CH), 127.0 (CH), 124.3 (CH), 122.7 (CH), 118.0 (CH); **HRMS** Found 267.0085 $[C_{12}H_8Cl_2N_2O+H]^+$ requires 267.0086.

2-Phenyl-*N*-(pyridin-2-yl)acetamide 14

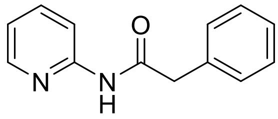

Reaction performed on a 5 mmol scale for 48 h. Purified using the standard resin work up, but without the use of Amberlyst A-15.

White solid, 73%; **M.p** 122-123 °C; **v_{max}** (solid/cm^{-1}) 3273 (NH), 2930 (CH), 1636 (CO), 1512 (CC); **^{1}H NMR** (400 MHz, $CDCl_3$) δ 8.29 – 8.16 (m, 2H, Ar*H*), 7.98 (s, 1H, N*H*), 7.72 – 7.64 (m, 1H, Ar*H*), 7.43 – 7.36 (m, 2H, Ar*H*), 7.35 – 7.29 (m, 3H, Ar*H*), 7.05 – 6.97 (m, 1H, Ar*H*), 3.76 (s, 2H, C*H₂*); **^{13}C NMR** (101 MHz, $CDCl_3$) δ 169.6 (C), 151.3 (C), 147.8 (CH), 138.5 (CH), 134.0 (C), 129.6 (CH), 129.3 (CH), 127.8 (CH), 120.0 (CH), 114.0 (CH), 45.1 (CH_2).

[Data in accordance with the literature *Org. Biomol. Chem*. **2015**, 13, 10888 - 10894]

2-Phenyl-*N*-(pyridin-3-yl)acetamide 15

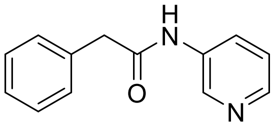

Reaction performed on a 5 mmol scale for 24 h. Purified by flash column chromatography (20% EtOAc in petrol).

White solid, 63%; **M.p** 109 – 110 °C; **v_{max}** (film/cm^{-1}) 3281 (NH), 2924 (CH), 1639 (CO), 1578, 1414, 1282 695. **^{1}H NMR** (600 MHz, $CDCl_3$) δ 8.42 (d, *J* = 2.3 Hz, 1H, Ar*H*), 8.31 (dd, *J* = 4.7, 1.4 Hz, 1H, Ar*H*), 8.10 (m, 1H, Ar*H*), 7.50 (m, 1H, N*H*), 7.40 (dd, *J* = 10.1, 4.5 Hz, 2H, Ar*H*), 7.37 – 7.30 (m, 3H, Ar*H*), 7.23 (dd, *J* = 8.4, 4.7 Hz, 1H, Ar*H*), 3.75 (s, 2H, C*H*$_2$). **^{13}C NMR** (151 MHz, $CDCl_3$) δ 169.7 (C), 145.5 (CH), 141.2 (CH), 134.7 (C), 134.1 (C), 129.5 (CH), 129.4 (CH), 127.9 (CH), 127.3 (CH), 123.7 (CH), 44.7 (CH_2). **HRMS** Found 213.1022 $[C_{13}H_{12}N_2O+Na]^+$ requires 213.1022.

***N*-(4-Fluorobenzyl)-2,2-diphenylacetamide 16**

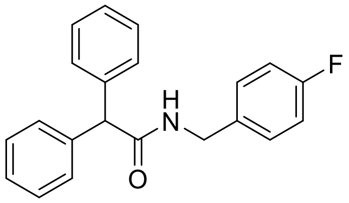

Reaction performed on a 5 mmol scale for 24 h. Purified using the standard resin work up.

White solid, 81%; **M.p** 136 – 138 °C; $\mathbf{v_{max}}$ (film/cm^{-1}) 3293 (NH), 2932 (CH), 1636 (CO), 1601, 1547, 1218; **^{1}H NMR** (400 MHz, $CDCl_3$) δ 7.35 – 7.30 (m, 4H, Ar*H*), 7.29 – 7.23 (m, 6H, Ar*H*), 7.20 – 7.15 (m, 2H,Ar*H*), 7.02 – 6.94 (m, 2H, Ar*H*), 5.88 (s, 1H, N*H*), 4.95 (s, 1H, PhC*H*CO), 4.44 (d, J = 5.9 Hz, 2H, ArCH_2N); **^{13}C NMR** (176 MHz, $CDCl_3$) δ 171.94 (C), 162.2 (d, $J_{C\text{-}F}$ = 245.8 Hz, C), 139.4 (C), 134.1 (d, J = 3.2 Hz, C), 129.5 (d, J = 8.1 Hz, CH), 129.0 (CH), 128.9 (CH), 127.5 (CH), 115.6 (d, J = 21.5 Hz, CH), 59.3 (CH), 43.2 (CH_2); **HRMS** Found 320.1447 $[C_{21}H_{18}FNO+H]^+$ requires 320.1445.

1-(Piperidin-1-yl)pentan-1-one 17

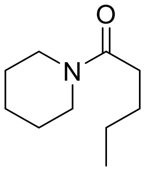

Reaction performed on a 5 mmol scale for 18 h. Purified using the standard resin work up.

Yellow oil, 91%; $\mathbf{v_{max}}$ (film/cm^{-1}) 2932 (CH), 2855 (CH), 1634 (CO); **^{1}H NMR** (700 MHz, $CDCl_3$) δ 3.40 (m, 2H, NCH_2), 3.26 (m, 2H, NCH_2), 2.18 (m, 2H, COCH_2), 1.64 – 1.29 (m, 8H, 4 × CH_2), 1.27 – 1.17 (m, 2H, CH_2CH$_3$), 0.79 (m, 3H, CH_3); **^{13}C NMR** (176 MHz, $CDCl_3$) δ 171.5 (C), 46.7 (CH_2), 42.6 (CH_2), 33.2 (CH_2), 27.6 (CH_2), 26.6 (CH_2), 25.6 (CH_2), 24.6 (CH_2), 22.6 (CH_2), 13.9 (CH_3).

[Data in accordance with the literature *Org. Lett.*, **2018**, 20, 5861 – 5865]

2-Phenyl-*N*-(1-phenylethyl)acetamide 18

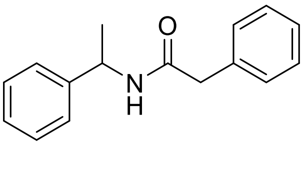

Reaction performed on a 5 mmol scale for 20 h. Standard resin work-up used.

White solid, 92%; **M. p** 102 – 105 °C; **v_{max}** (film/cm^{-1}): 3318 (NH), 2974 (CH), 1646 (CO), 1525 (CC); **^{1}H NMR** (600 MHz; $CDCl_3$) *δ* 7.36 – 7.19 (10 H, m, Ar*H*), 5.80 (1H, br s, N*H*), 5.12 (1H, quin., *J* = 7.2 Hz, C*H*), 3.56 (2H, s, CH_2), 1.40 (3H, d, *J* = 6.9 Hz, CH_3); **^{13}C NMR** (176 MHz, $CDCl_3$) *δ* 170.2 (C), 143.3 (C), 135.2 (C), 129.5 (CH), 129.1 (CH), 128.8 (CH), 127.5 (CH), 127.4 (CH), 126.1 (CH), 48.9 (CH), 44.0 (CH_2), 22.0 (CH_3); **MS**: LC-MS ES$^+$ [M+H$^+$] 240

[Data in accordance with the literature *Green Chem.* **2011**, 13, 835-838]

***N*-Benzyl-2-(2-chlorophenyl)-2-hydroxyacetamide 19**

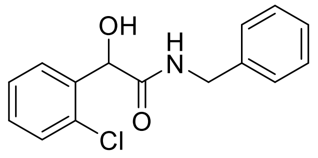

Reaction performed on a 5 mmol scale for 40 h. Purified using the standard resin work up.

Off white solid; 38%; **M.p** 144-146 °C; **v_{max}** (solid/cm^{-1}) 3264 (NH), 3195 (OH), 1919 (CH), 1633 (CO), 1532 (CC); **^{1}H NMR** (700 MHz, $CDCl_3$) *δ* 7.35 – 7.27 (m, 7H, Ar*H*), 7.17 (d, *J* = 7.2 Hz, 2H, Ar*H*), 6.76 – 6.56 (m, 1H, N*H*), 5.03 (d, *J* = 2.4 Hz, 1H, C*H*), 4.45 – 4.36 (m, 2H, CH_2), 3.81 (d, *J* = 3.3 Hz, 1H, O*H*); **^{13}C NMR** (176 MHz, $CDCl_3$) *δ* 171.8 (C), 138.0 (C), 137.7 (C), 134.6 (C), 129.1 (CH), 128.9 (CH), 128.2 (CH), 128.0 (CH), 127.84 (CH), 127.77 (CH), 127.7 (CH), 73.7 (CH), 43.6 (CH_2); **HRMS** (ESI) Found: 276.0786. $C_{15}H_{14}NO_2Cl$+H ([M+H]$^+$): requires 276.0786.

[It should be noted that the chemical shifts of the CH peak at 5.03 ppm and the OH at 3.81 ppm are concentration dependent. Data in accordance with the literature *Adv. Synth. Catal.* **2011**, 353, 1234 – 1240]

***N*-(3,4-Dimethoxyphenethyl)hexanamide 20**

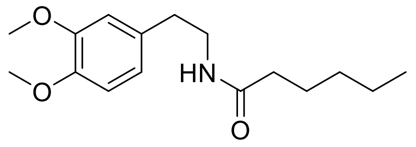

Reaction performed on a 5 mmol scale for 20 h. Purified by flash column chromatography (40% EtOAc in petrol).

White solid, 70%; **M. p** 61 – 63 °C; **v_{max}** (film/cm^{-1}): 3313 (NH), 2949 (CH), 1635 (CO); **^{1}H NMR** (700 MHz; $CDCl_3$) *δ* 6.80 (1H, d, *J* = 7.8 Hz, Ar*H*), 6.73 – 6.71 (2H, m, Ar*H*), 5.48 (1H, br. s,

N*H*), 3.86 (3H, s, OC*H*$_3$), 3.85 (3H, s, OC*H*$_3$), 3.49 (m, C*H*$_2$NH), 2.75 (2H, t, *J* = 6.9 Hz, ArC*H*$_2$), 2.11 (2H, t, *J* = 7.6 Hz, OCC*H*$_2$), 1.59 (2H, quin, *J* = 7.6 Hz, OCCH$_2$C*H*$_2$), 1.27 (4H, m, OCCH$_2$CH$_2$C*H*$_2$C*H*$_2$), 0.88 (3H, t, *J* = 7.1 Hz, CH$_2$C*H*$_3$); **^{13}C NMR** (175 MHz, CDCl$_3$) *δ* 173.2 (C), 149.2 (C), 147.8 (C), 131.6 (C), 120.8 (CH), 112.0 (CH), 111.4 (CH), 56.0 (CH$_3$), 56.0 (CH$_3$), 40.7 (CH$_2$), 36.9 (CH$_2$), 35.4 (CH$_2$), 31.6 (CH$_2$), 25.6 (CH$_2$), 22.5 (CH$_2$), 14.1 (CH$_3$); **MS**: LC-MS ES$^+$ [M$^+$] 279.9

Yield in TAME 97%

[Data in accordance with the literature *J. Am. Chem. Soc*. **1952,** 74, 1316–1317]

***N*-(2-Hydroxy-2-phenylethyl)hexanamide 21**

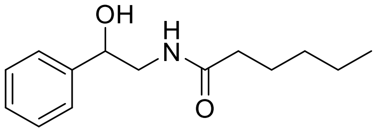

Reaction performed on a 5 mmol scale for 20 h. Purified by flash column chromatography (50% EtOAc in petrol).

White solid, 54%; **M.p** 78 – 81 °C; **v_{max}** (film/cm^{-1}): 3384 (NH), 3287 (OH), 2924 (CH), 1609 (CO), 1560 (CC); 236.1651; **^{1}H NMR** (700 MHz, CDCl$_3$) *δ* 7.35 – 7.31 (m, 4H, Ar*H*), 7.29 – 7.26 (m, 1H, Ar*H*), 6.04 (s, 1H, N*H*), 4.83 – 4.79 (m, 1H, OHC*H*), 3.81 (s, 1H, O*H*), 3.66 (ddd, *J* = 14.1, 6.8, 3.3 Hz, 1H, NC*H*$_2$), 3.31 (ddd, *J* = 14.0, 7.7, 5.0 Hz, 1H, NC*H*$_2$), 2.17 – 2.12 (m, 2H, OCC*H*$_2$), 1.59 (m, 2H, OCCH$_2$C*H*$_2$), 1.34 – 1.23 (m, 4H, OCCH$_2$CH$_2$C*H*$_2$C*H*$_2$), 0.88 (t, *J* = 7.2 Hz, 3H, C*H*$_3$); **^{13}C NMR** (176 MHz, CDCl$_3$) *δ* 175.0 (C), 142.0 (C), 128.6 (CH), 127.9 (CH), 126.0 (CH), 73.9 (CH), 47.6 (CH$_2$), 36.7 (CH$_2$), 31.5 (CH$_2$), 25.5 (CH$_2$), 22.5 (CH$_2$), 14.1 (CH$_3$); **MS:** LC-MS ES$^+$ [M+H$^+$] 236.0; **HRMS** ESI Found 236.1654 [$C_{14}H_{21}NO_2$+H]$^+$ requires 236.1651.

Yield in TAME using 1.1 equiv. of amine 50%

***tert*-Butyl (*tert*-butoxycarbonyl)-D-alanyl-L-phenylalaninate 22**

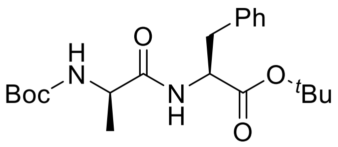

Reaction performed on a 3 mmol scale for 16 h. Purified using the standard resin work up.

White solid, 95%; **[α]$_D^{24.7}$** +43.6 (*c* 1.0, $CHCl_3$); **M.p** 96-99 °C; **v_{max}** (solid/cm^{-1}) 3293 (NH), 2974 (CH), 2930 (CH), 1712 (CO), 1628 (CO); **^{1}H NMR** (700 MHz, $CDCl_3$) *δ* 7.25 (t, *J* = 7.1 Hz, 2H, Ar*H*), 7.20 (t, *J* = 7.2 Hz, 1H, Ar*H*), 7.13 (d, *J* = 7.5 Hz, 2H, Ar*H*), 6.61 (d, *J* = 7.3 Hz, 1H, N*H*), 5.09 (s, 1H, N*H*), 4.69 (m, 1H, C*H*CH_3), 4.15 (br s, 1H, C*H*CH_2), 3.10 – 3.02 (m, 2H, CH_2), 1.41 (s, 9H, C(CH_3)$_3$), 1.37 (s, 9H, C(CH_3)$_3$), 1.29 (d, *J* = 6.5 Hz, 3H, CHCH_3); **^{13}C NMR** (176 MHz, $CDCl_3$) *δ* 172.4 (C), 170.4 (C), 155.5 (C), 136.2 (C), 129.7 (CH), 128.5 (CH), 127.1 (CH), 82.5 (C), 80.1 (C), 53.8 (CH), 50.3 (CH), 38.1 (CH_2), 28.4 (CH_3), 28.0 (CH_3), 18.6 (CH_3). Yield in TAME 96%

[Data in accordance with the literature *Sci. Adv.*, **2017**, 3, e1701028]

***tert*-Butyl (*tert*-butoxycarbonyl)-L-alanyl-L-phenylalaninate 23**

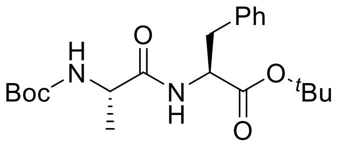

Reaction performed on a 3 mmol scale for 16 h. Purified using the standard resin work up.

White solid, 79%; **[α]$_D^{24.7}$** -43.4 (*c* 1.0, $CHCl_3$); **v_{max}** (solid/cm^{-1}) 3303 (NH), 2974 (CH), 2931 (CH), 2854 (CH), 1712 (CO), 1632 (CO); **^{1}H NMR** (700 MHz, $CDCl_3$) *δ* 7.26 (t, *J* = 7.2 Hz, 2H, Ar*H*), 7.21 (m, 1H, Ar*H*), 7.14 (d, *J* = 7.4 Hz, 2H, Ar*H*), 6.58 (s, 1H, N*H*), 5.02 (d, *J* = 6.5 Hz, 1H, N*H*), 4.73 (m, 1H, C*H*CH_3), 4.17 (br s, 1H, C*H*CH_2), 3.14 – 3.02 (m, 2H, CH_2), 1.42 (s, 9H, (CH_3)$_3$), 1.39 (s, 9H, (CH_3)$_3$), 1.29 (d, *J* = 7.0 Hz, 3H, CHCH_3); **^{13}C NMR** (176 MHz, $CDCl_3$) *δ* 172.2 (C), 170.5 (C), 155.5 (C), 136.2 (C), 129.7 (CH), 128.5 (CH), 127.1 (CH), 82.5 (C), 80.2 (C), 53.6 (CH), 50.2 (CH), 38.2 (CH_2), 28.4 (CH_3), 28.1 (CH_3), 18.8 (CH_3).

1-Methyl-*N*-((1R,3r,5S)-9-methyl-9-azabicyclo[3.3.1]nonan-3-yl)-1H-indazole-3-carboxamide 24

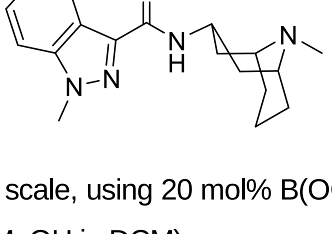

Reaction performed on a 2.5 mmol scale, using 20 mol% $B(OCH_2CF_3)_3$ for 72 h. Purified by flash column chromatography (0 – 10% MeOH in DCM).

White waxy solid, 45%. **v_{max}** (solid/cm^{-1}) 3409 (NH), 2925 (CH), 2087 (CH), 1636 (CO), 1530 (CC); **^{1}H NMR** (700 MHz, $CDCl_3$) *δ* 8.38 (dt, *J* = 8.2, 1.0 Hz, 1H, Ar*H*), 7.41 – 7.38 (m, 1H,

Ar*H*), 7.37 (dt, *J* = 8.5, 0.9 Hz, 1H, Ar*H*), 7.25 (m, 1H, Ar*H*), 6.78 (d, *J* = 8.2 Hz, 1H, N*H*), 4.55 (tdt, *J* = 11.4, 8.4, 6.7 Hz, 1H, NHC*H*), 4.07 (s, 3H, ArNC*H*$_3$), 3.08 (d, *J* = 10.9 Hz, 2H, NC*H*), 2.55 – 2.51 (m, 2H, NHCHC*H*$_2$), 2.50 (s, 3H, NC*H*$_3$), 2.01 – 1.92 (m, 3H, CH$_2$C*H*$_2$CH and C*H*$_2$CH$_2$CH), 1.54 – 1.50 (m, 1H, C*H*$_2$CH$_2$CH), 1.37 (ddd, *J* = 11.6, 8.9, 3.2 Hz, 2H, NHCHC*H*$_2$), 1.08 – 1.02 (m, 2H, CH$_2$C*H*$_2$CH); **^{13}C NMR** (176 MHz, CDCl$_3$) *δ* 162.0 (C), 141.4 (C), 137.7 (C), 126.9 (CH), 123.2 (CH), 122.9 (C), 122.6 (CH), 109.1 (CH), 51.4 (CH), 40.83 (CH), 40.76 (CH$_3$), 36.0 (CH$_3$), 33.2 (CH$_2$), 25.0 (CH$_2$), 14.5 (CH$_2$).
[Data in accordance with the literature *Sci. Adv.*, **2017**, 3, e1701028]

6. ^{1}H and ^{13}C NMR

N-(Cyclohexylmethyl)picolinamide 1

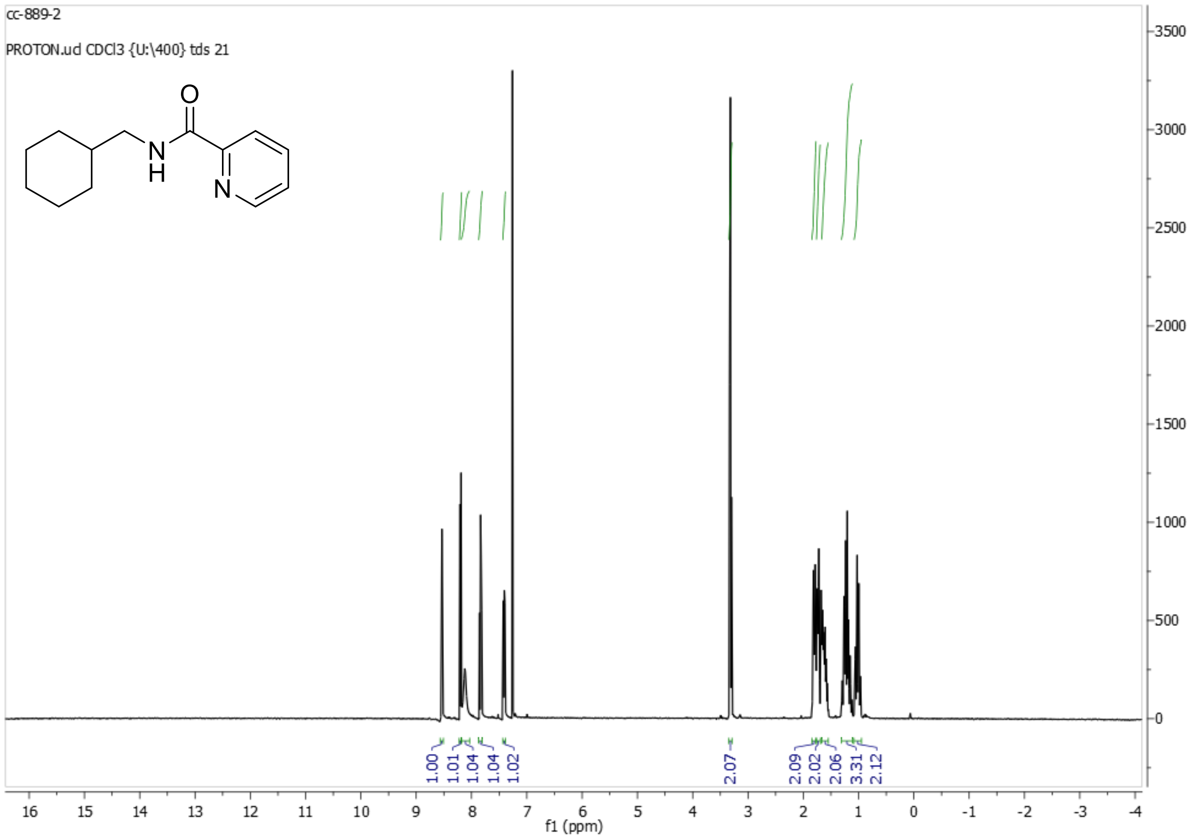

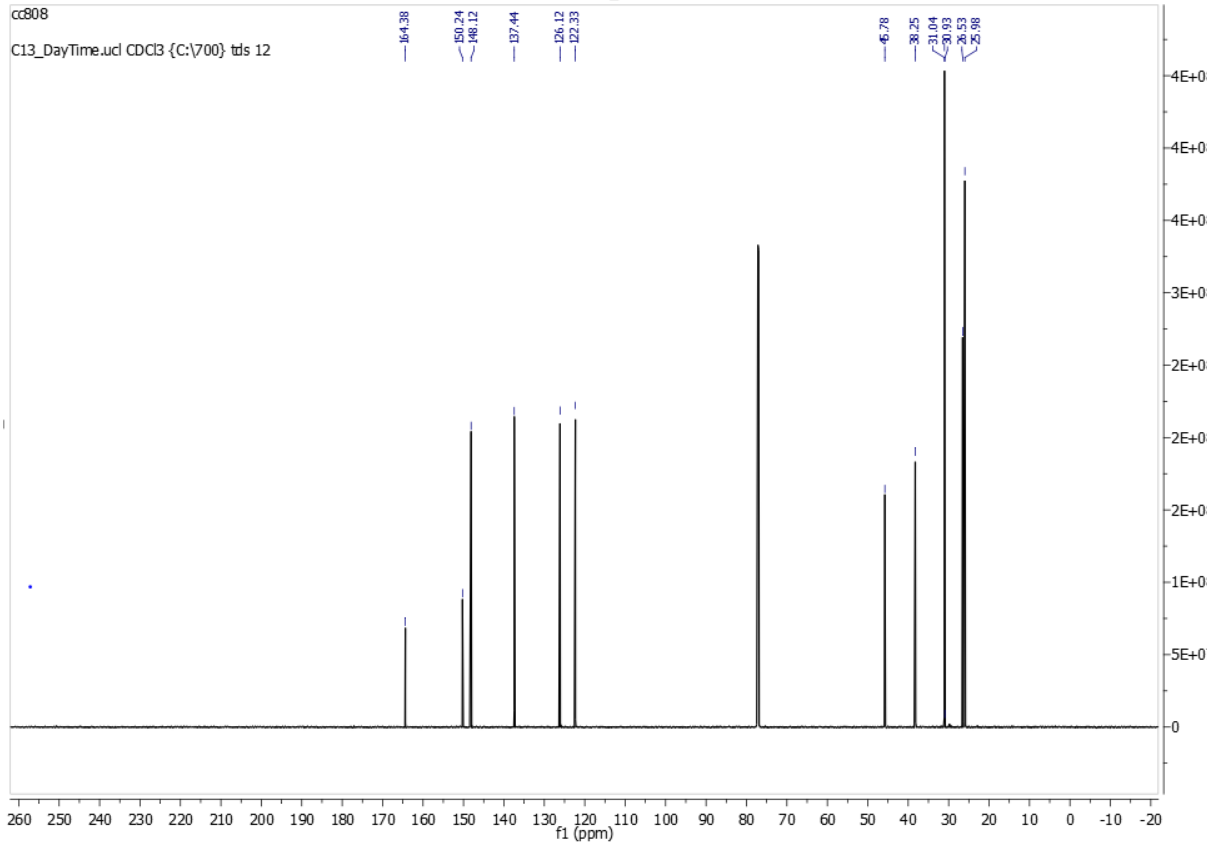

N-Cyclohexylpicolinamide 2

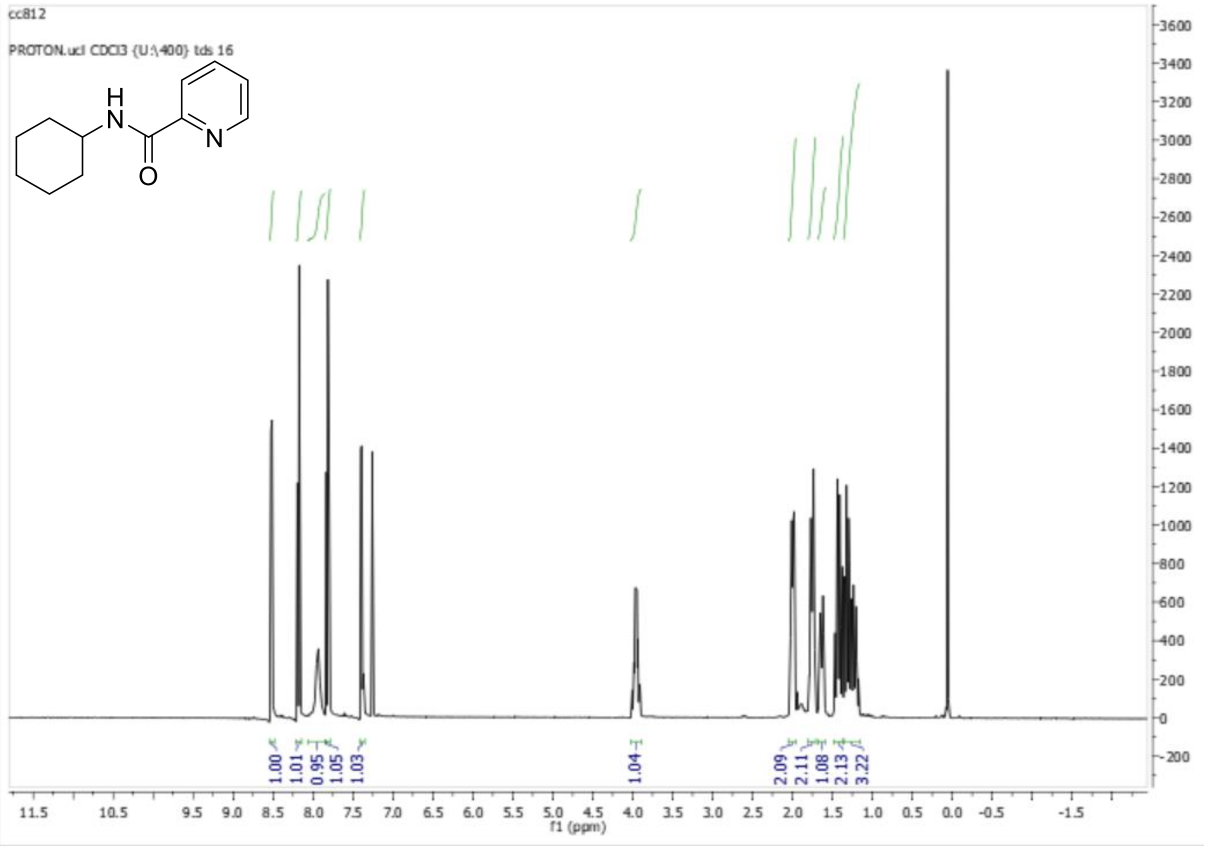

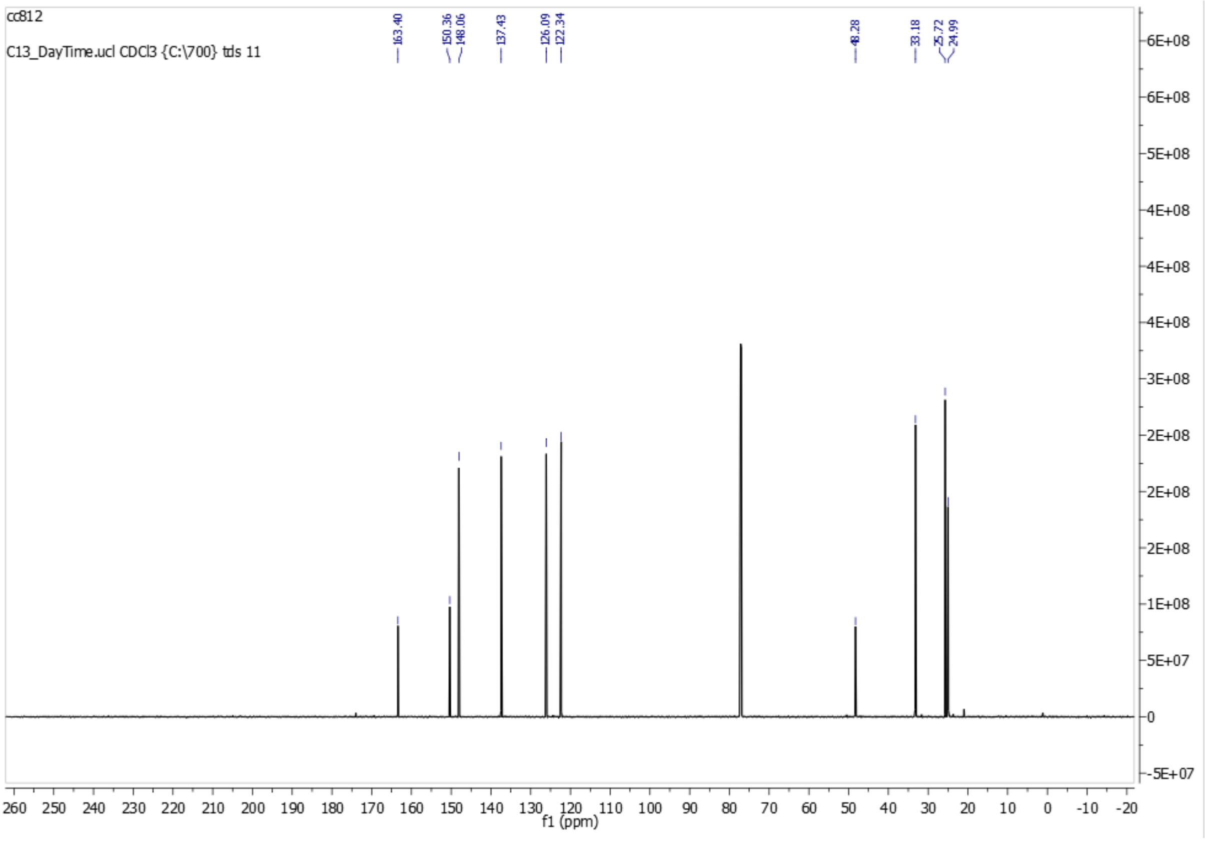

N-(4-Methoxybenzyl)quinoline-4-carboxamide 3

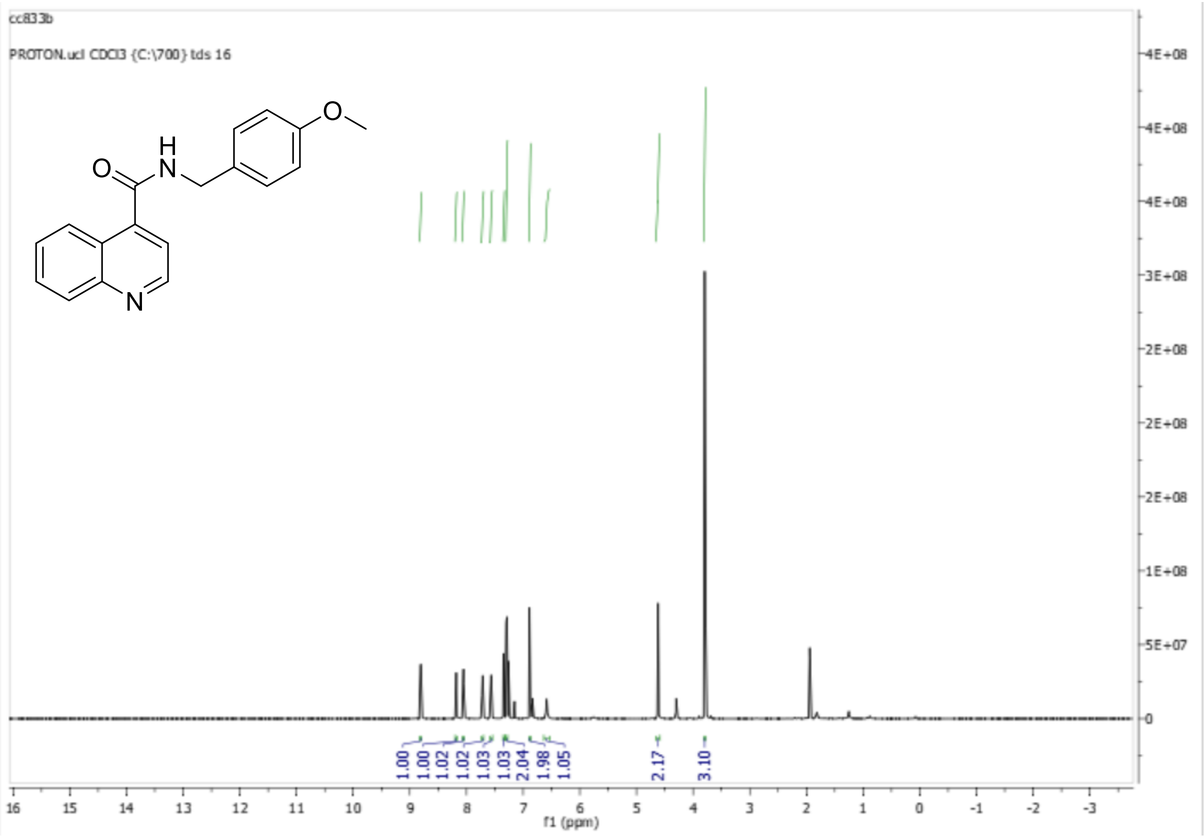

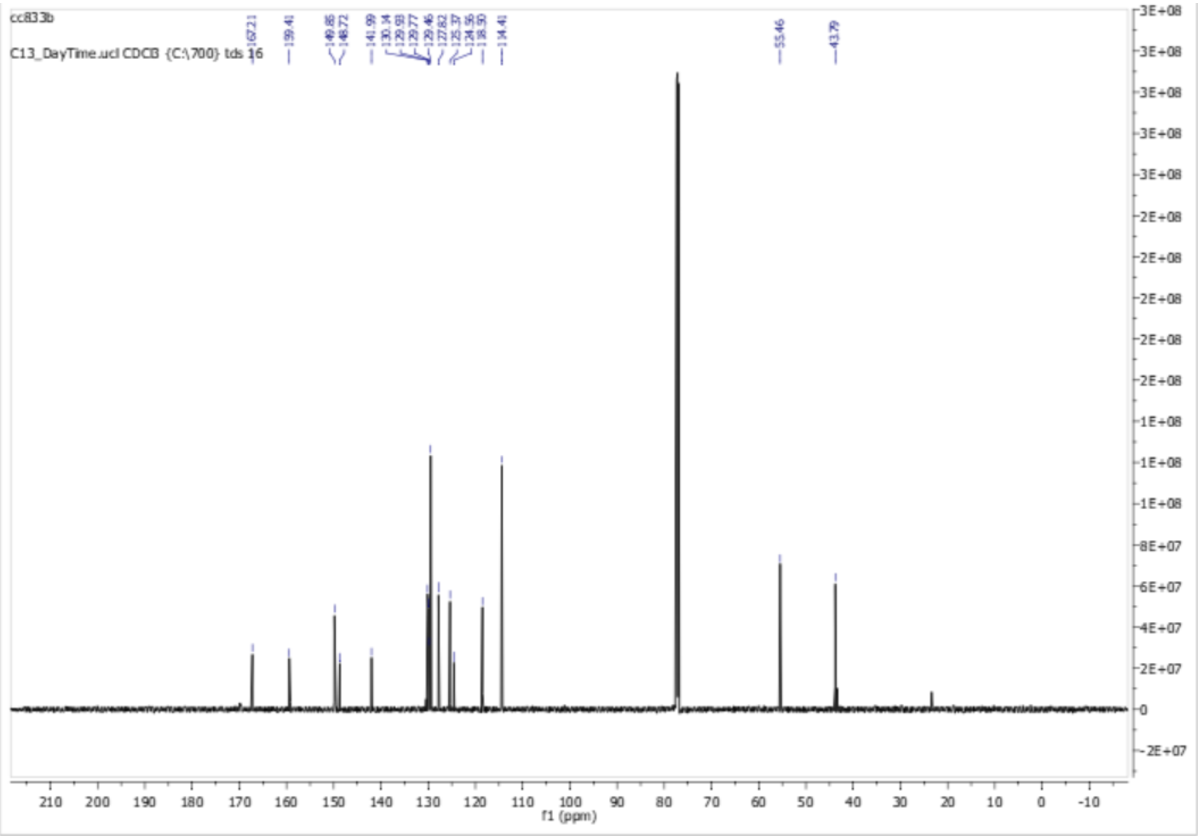

N-Benzyltetrahydrofuran-2-carboxamide 4

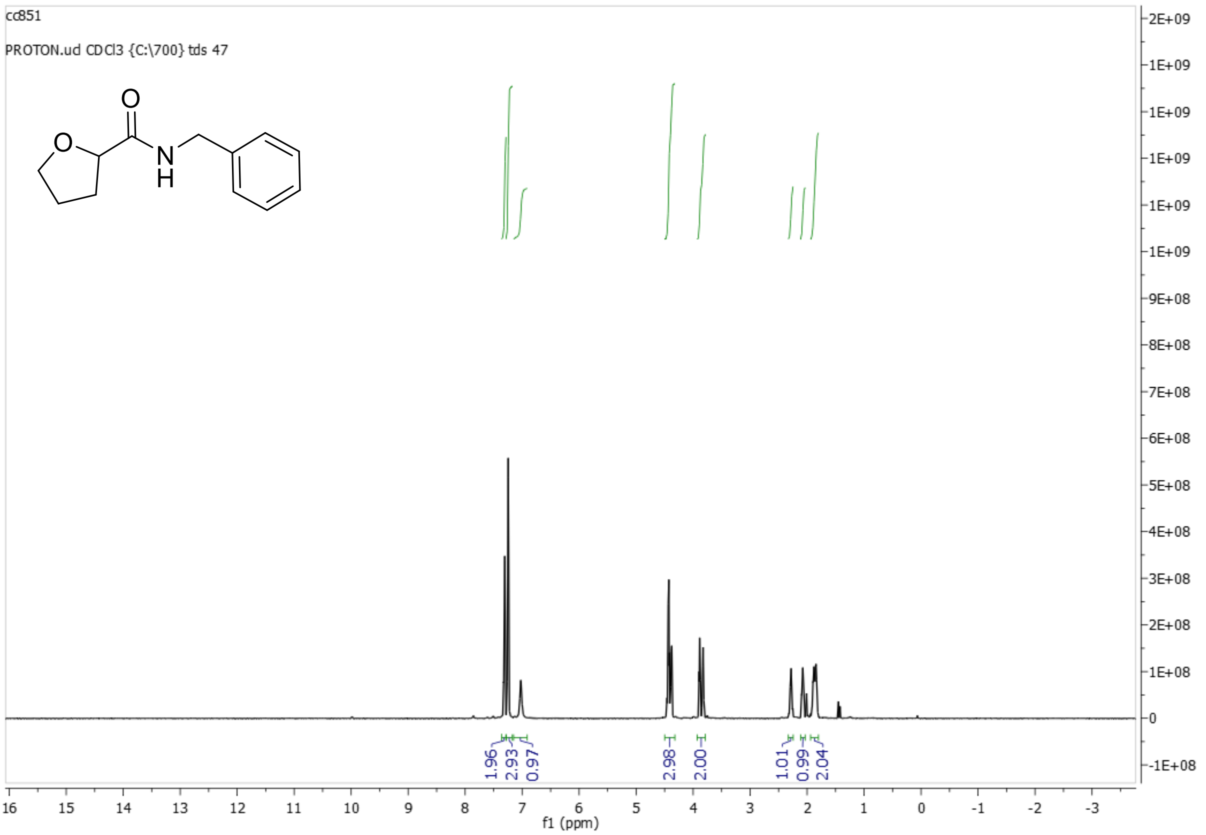

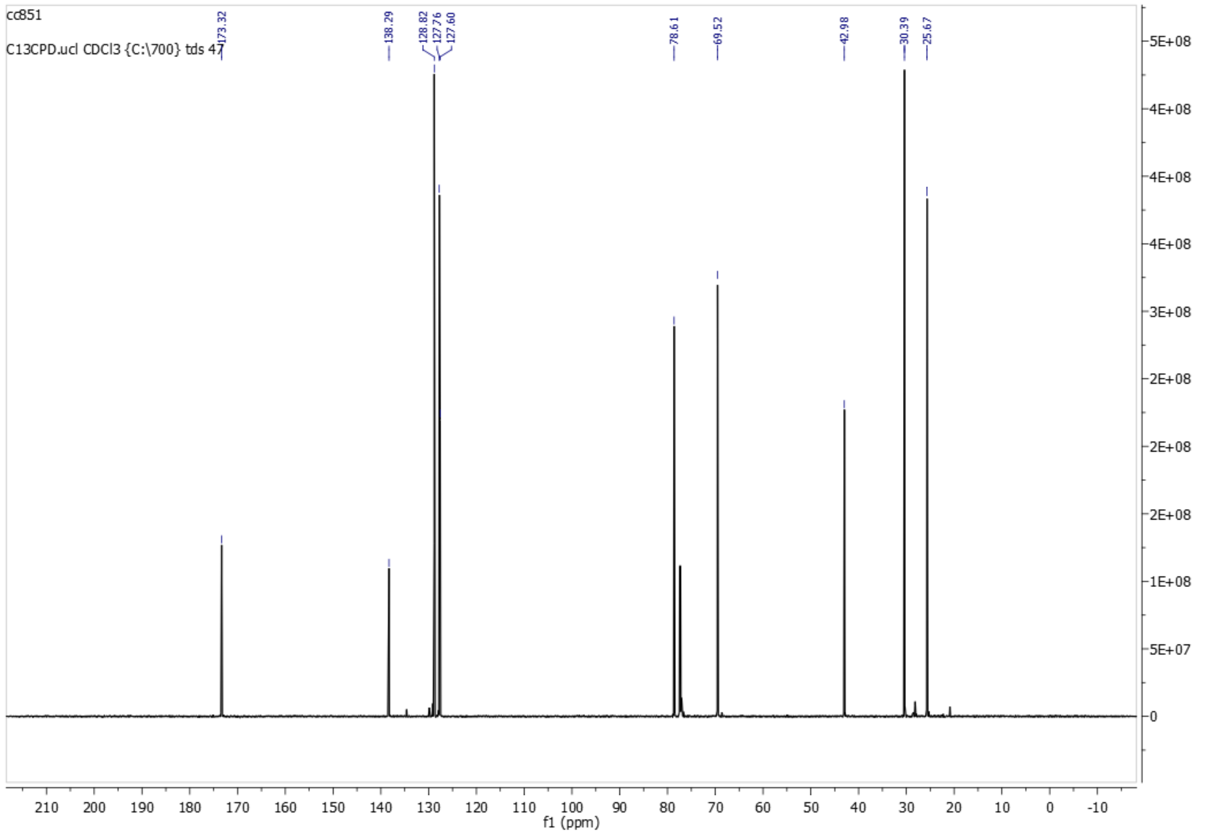

***tert*-Butyl 4-(tetrahydrofuran-2-carbonyl)piperazine-1-carboxylate 5**

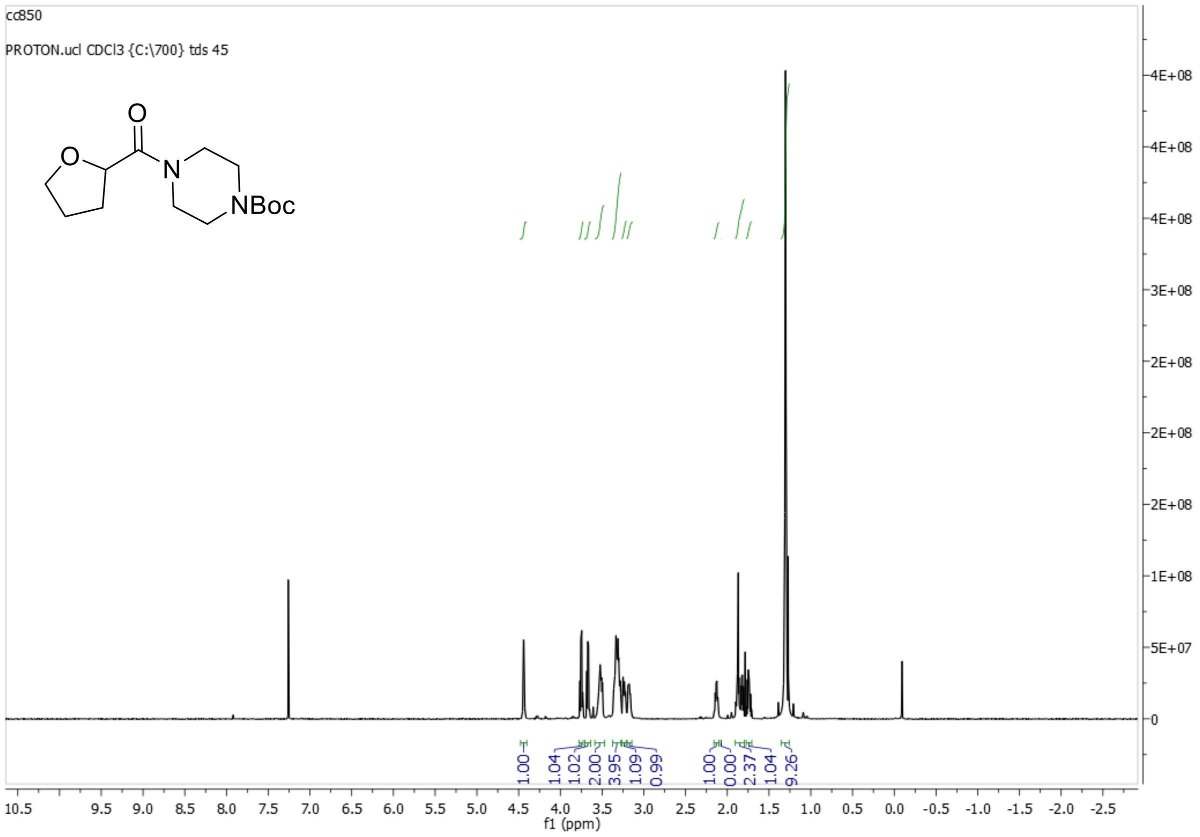

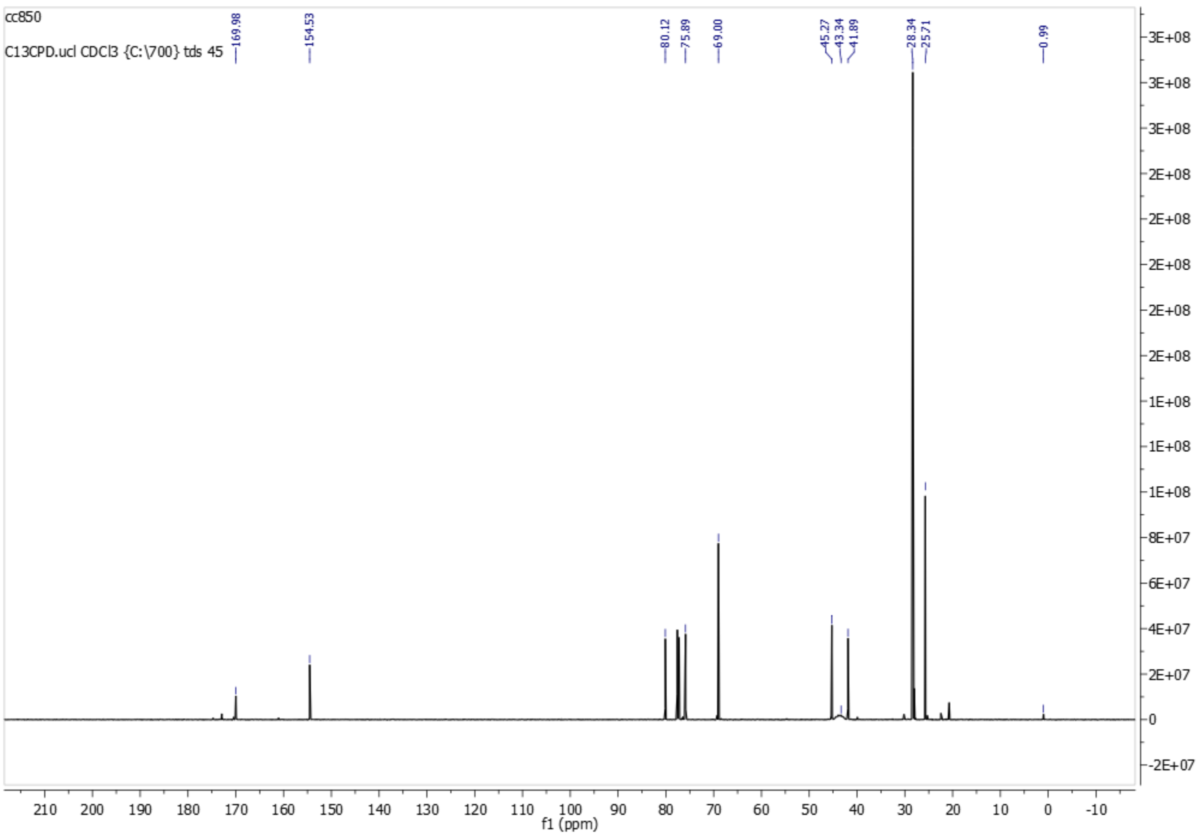

1-(Piperidin-1-yl)-2-(thiophen-2-yl)ethan-1-one 6

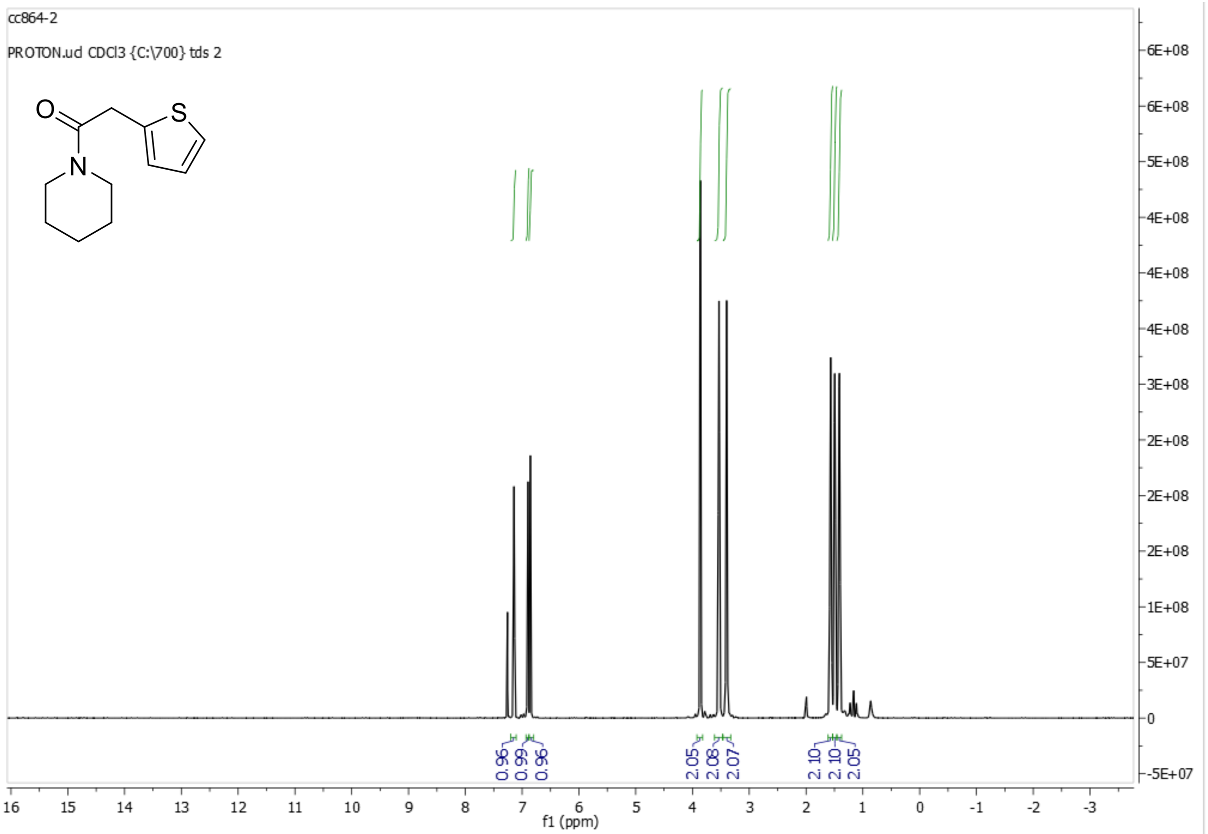

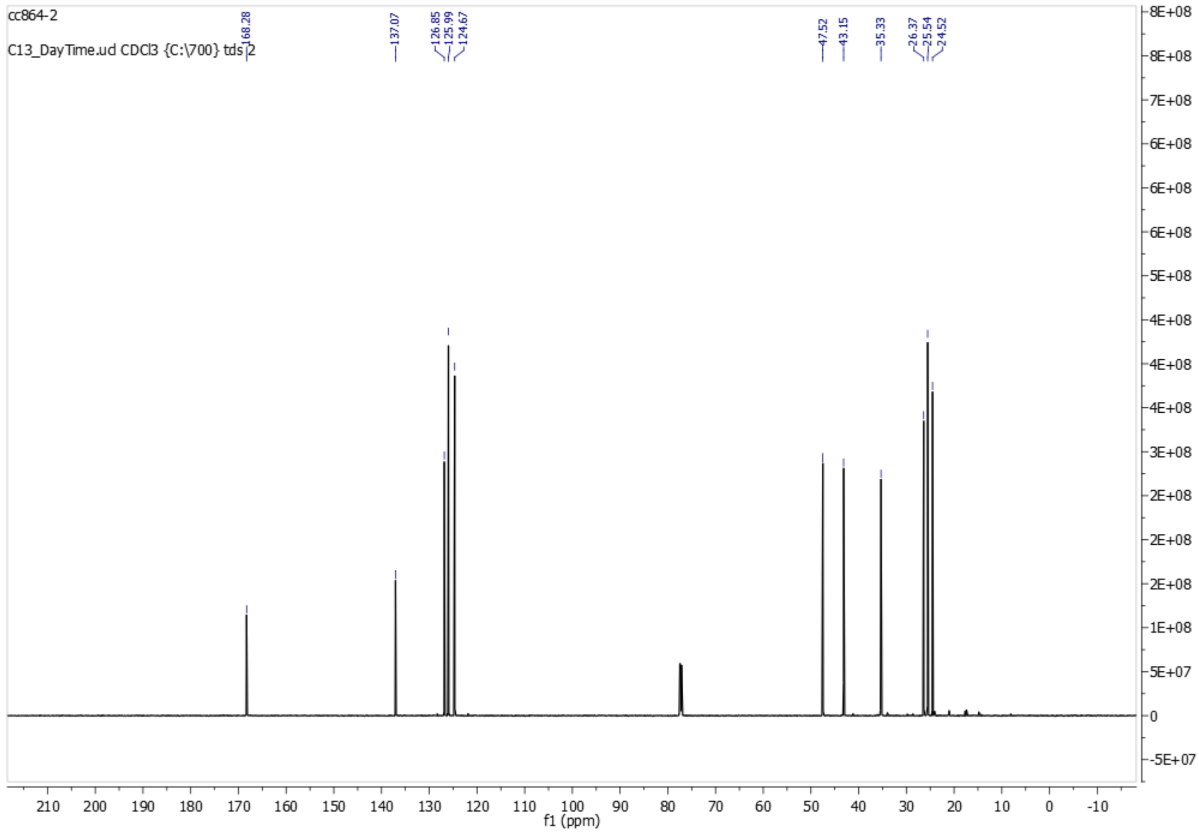

Indolin-1-yl(pyridin-2-yl)methanone 7

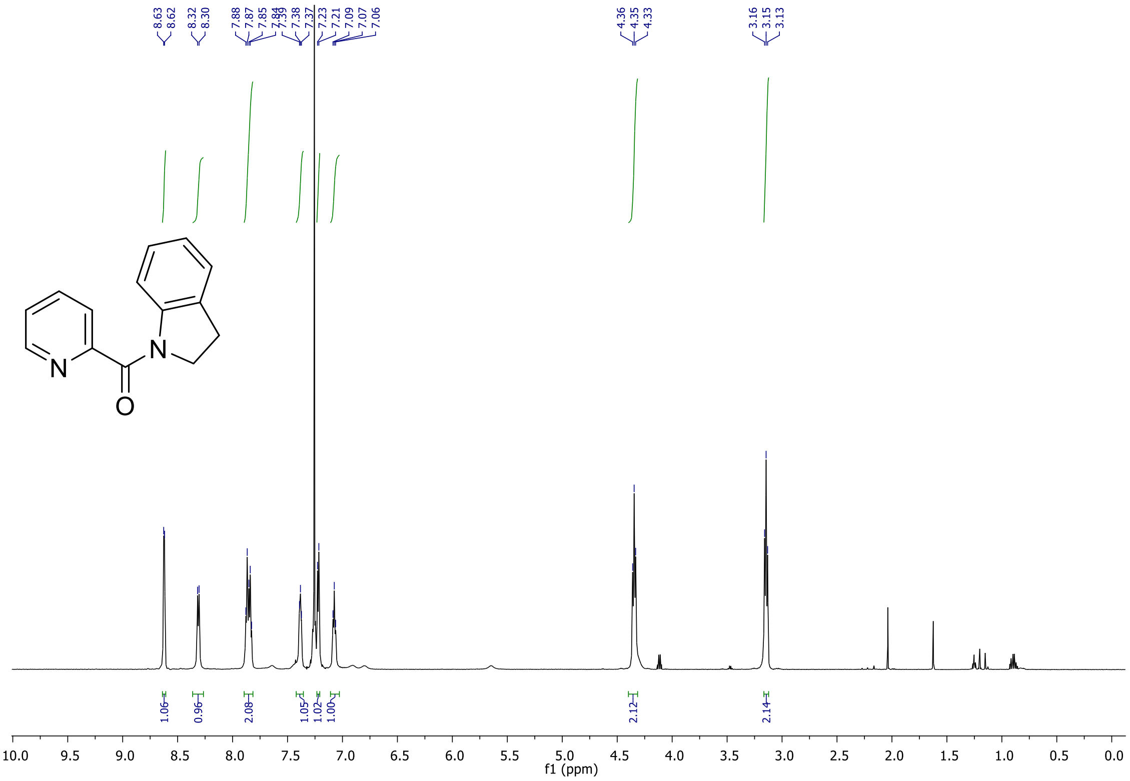

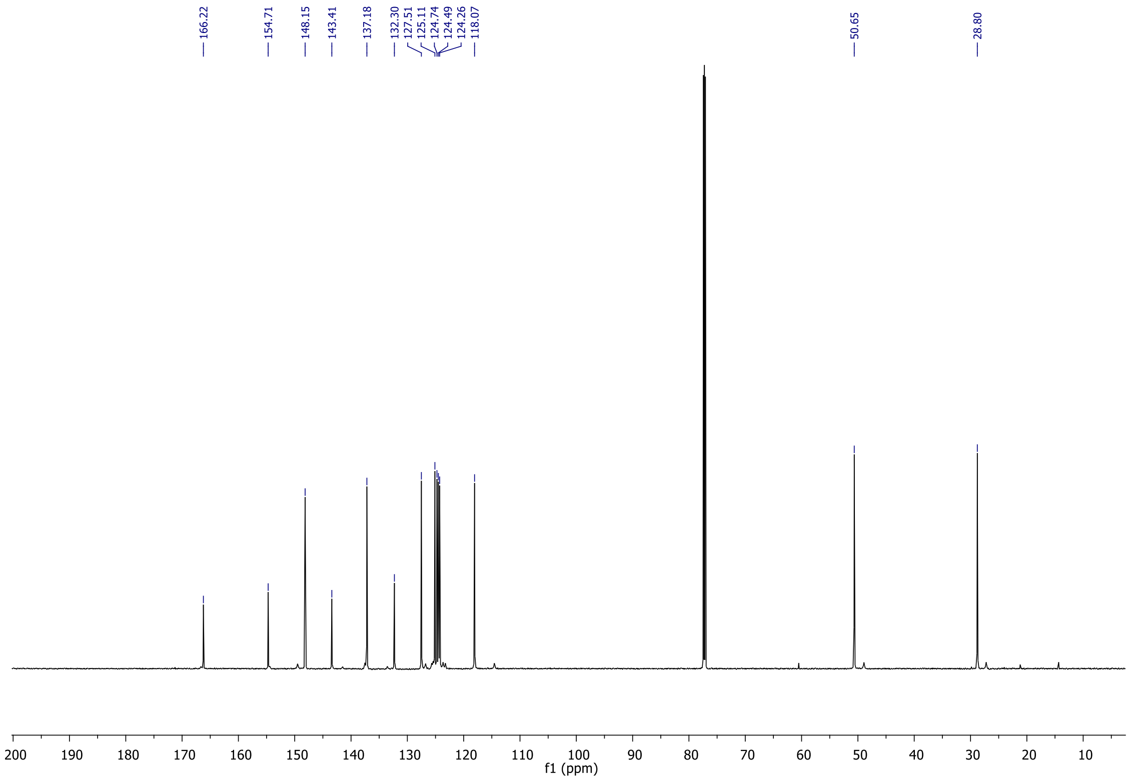

***N*-(2-Methoxyphenyl)-2-phenylacetamide 8**

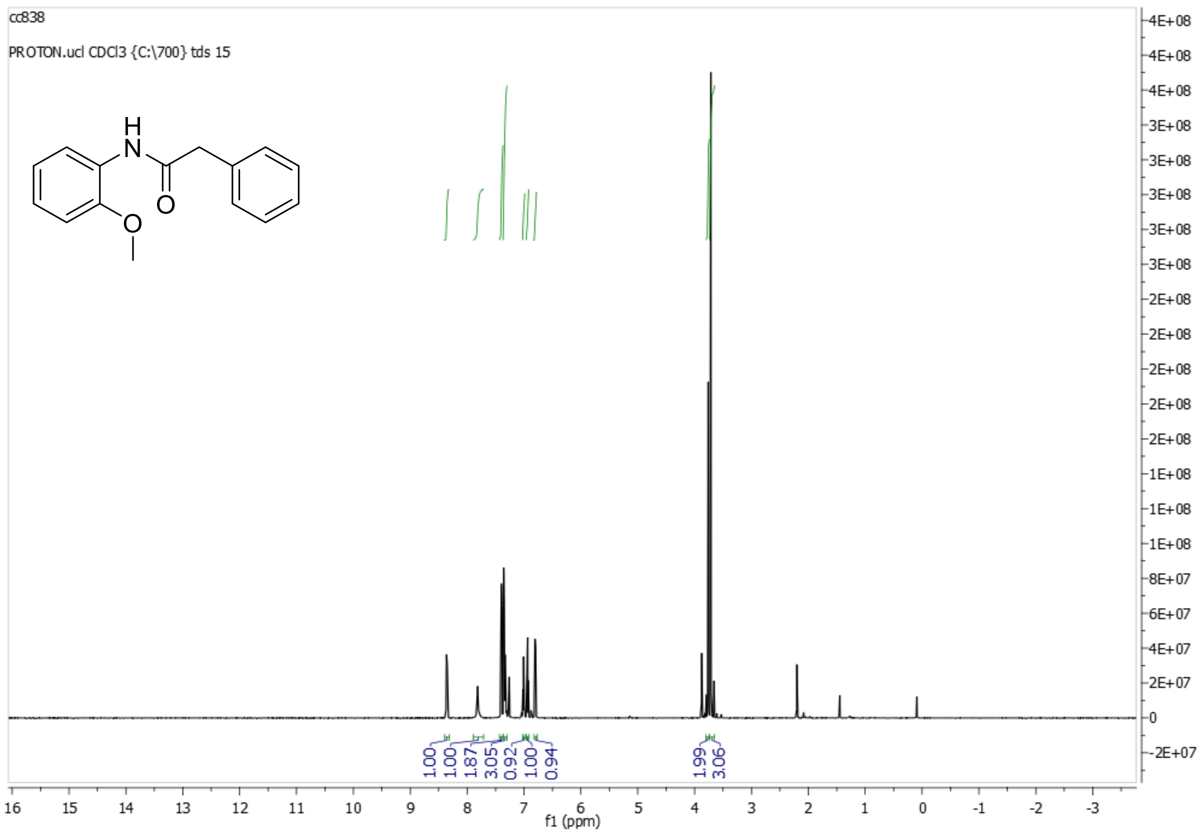

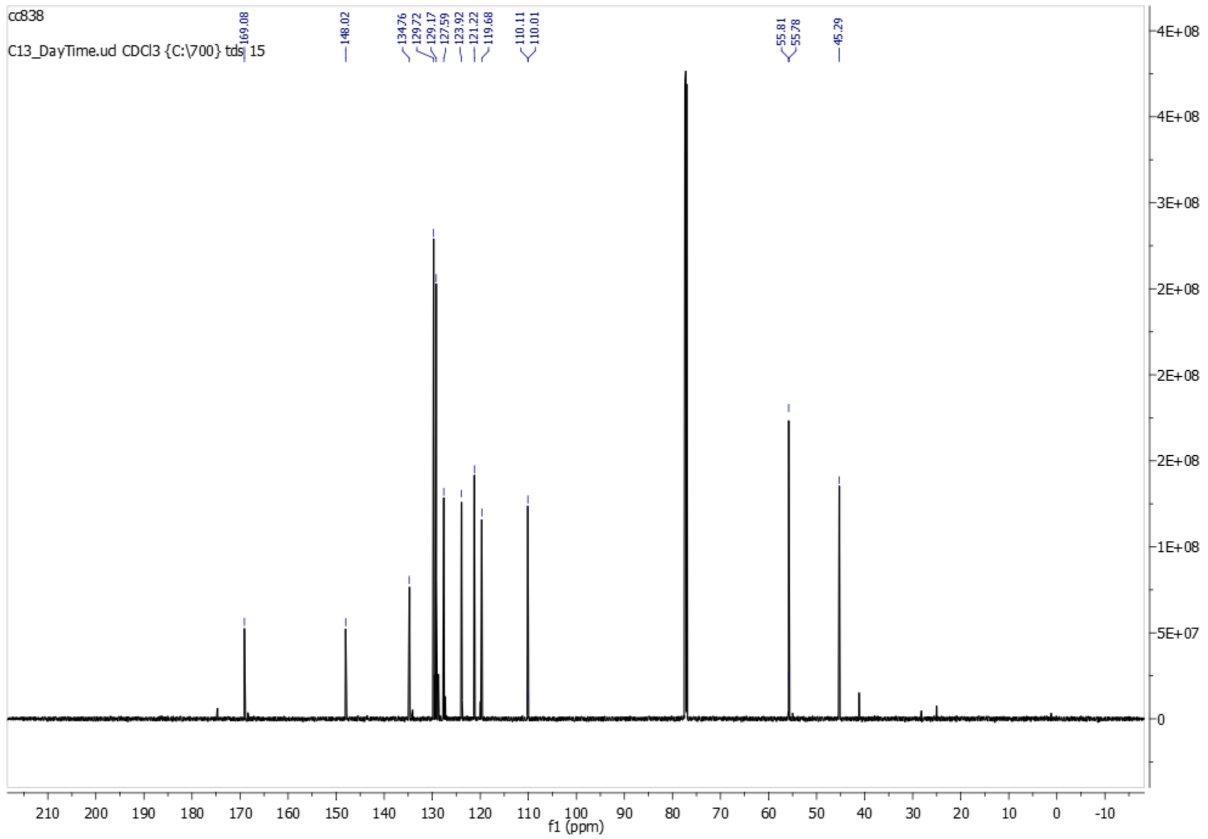

N-(2-Hydroxyphenyl)-2-phenylacetamide 9

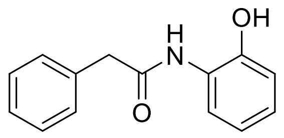

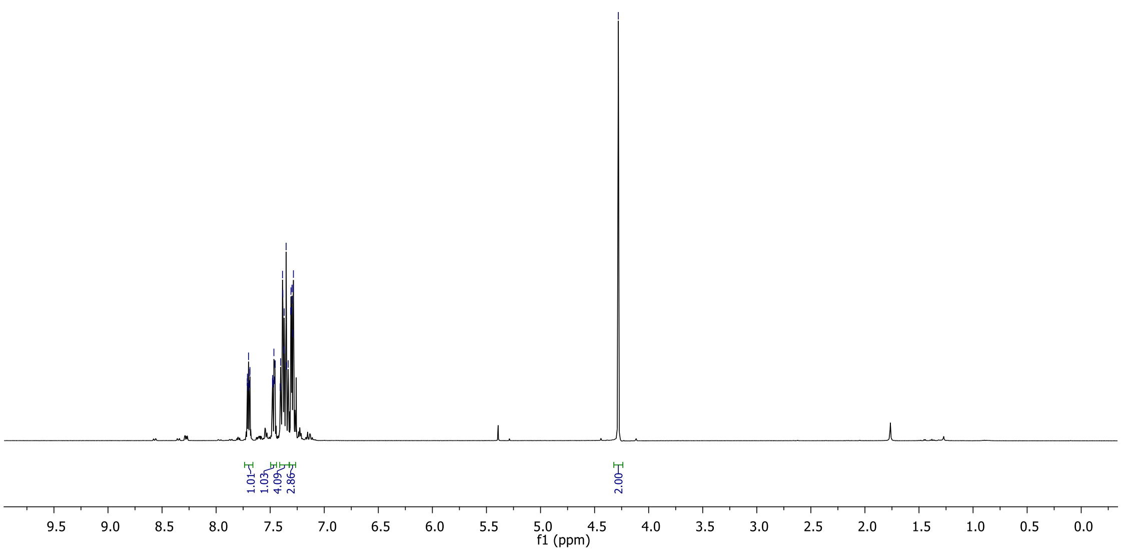

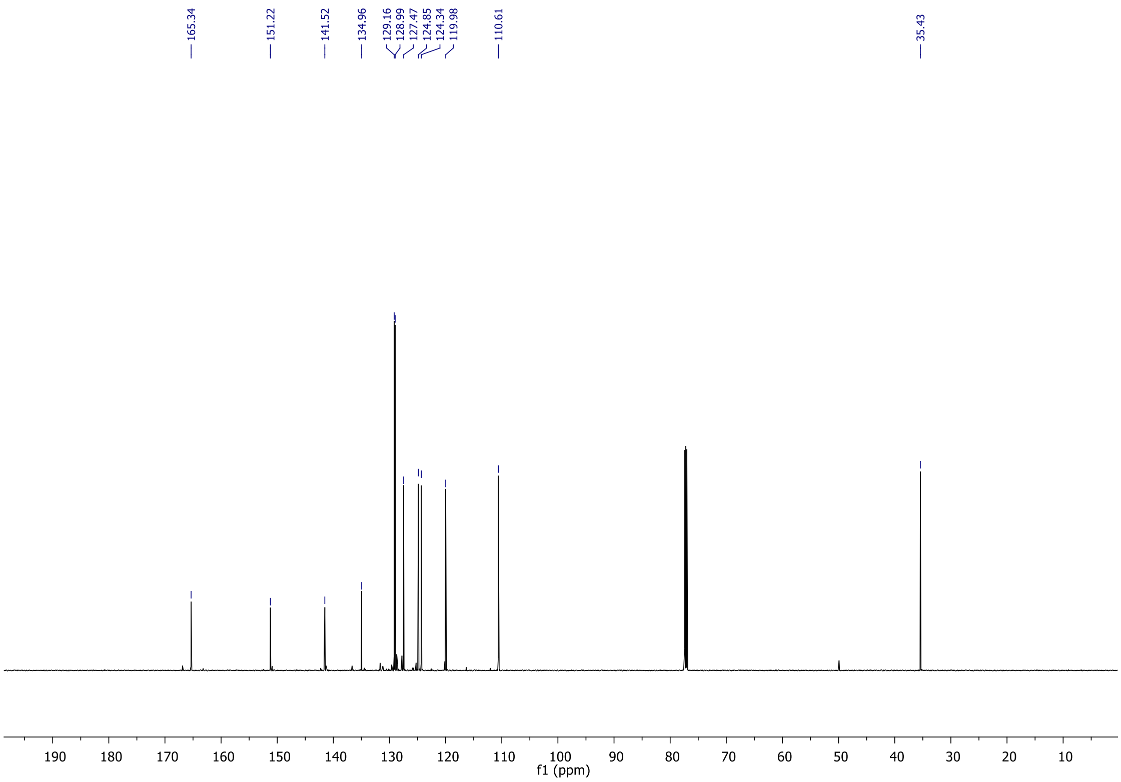

N-Mesityl-2-phenylacetamide 10

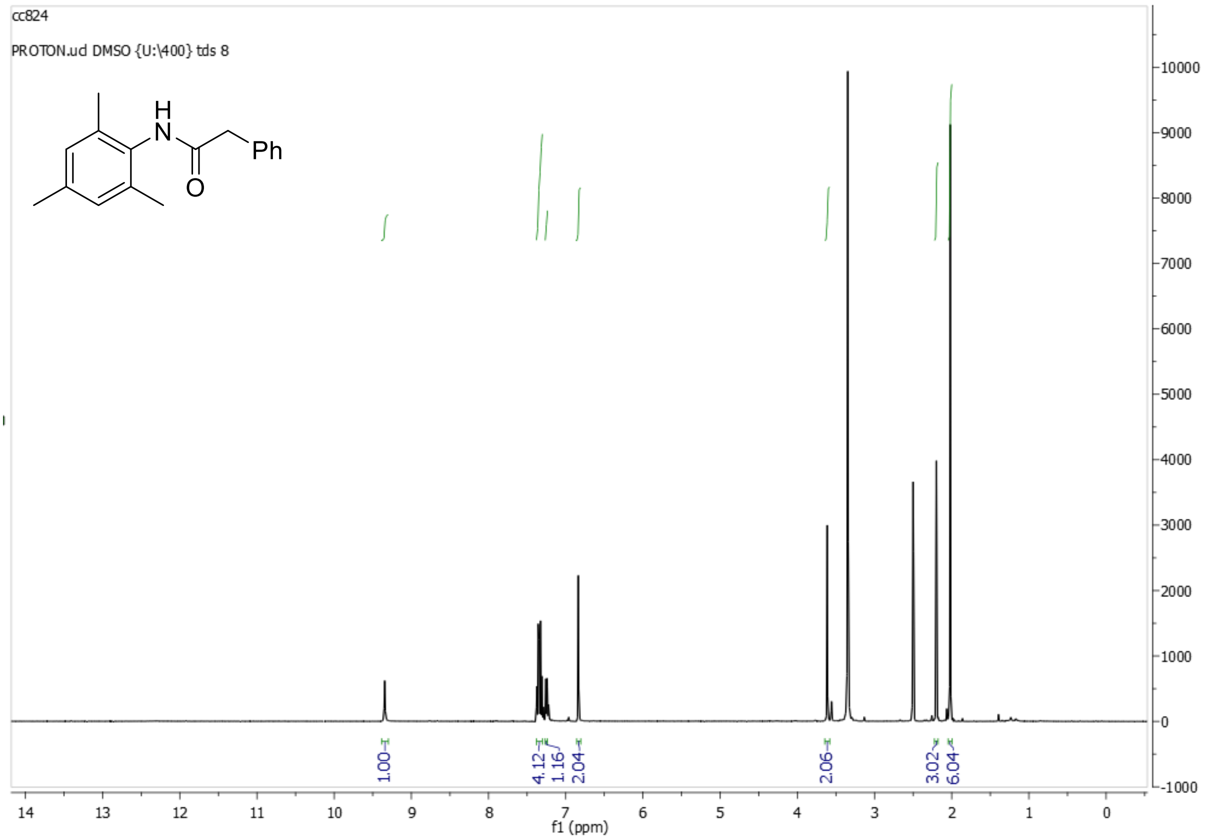

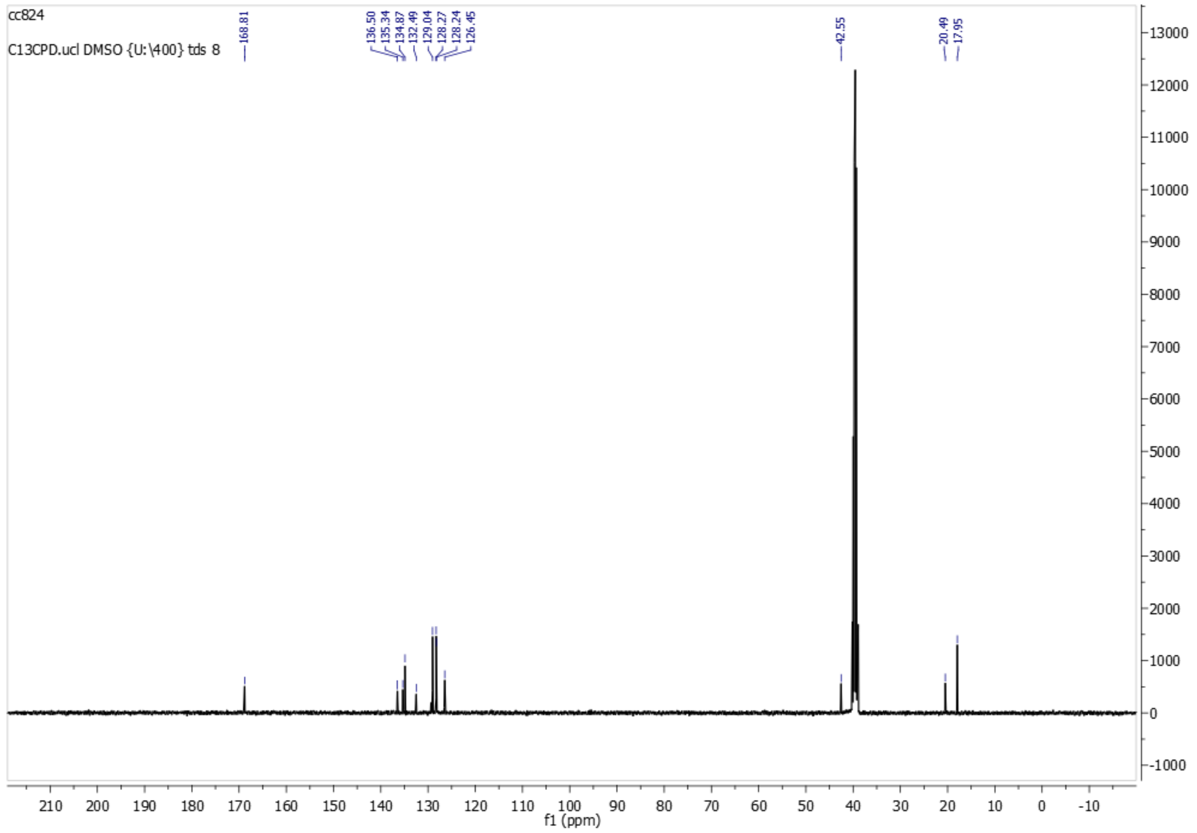

2-Phenyl-*N*-(3-(trifluoromethyl)phenyl)acetamide 11

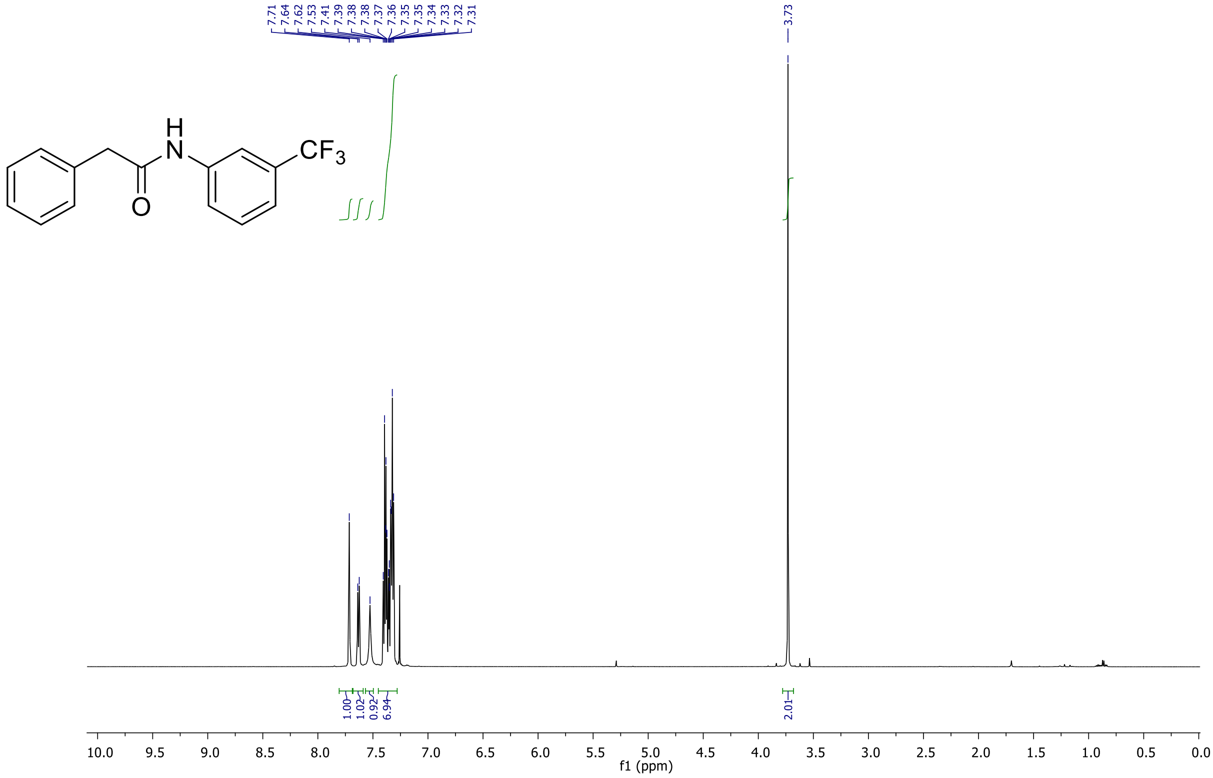

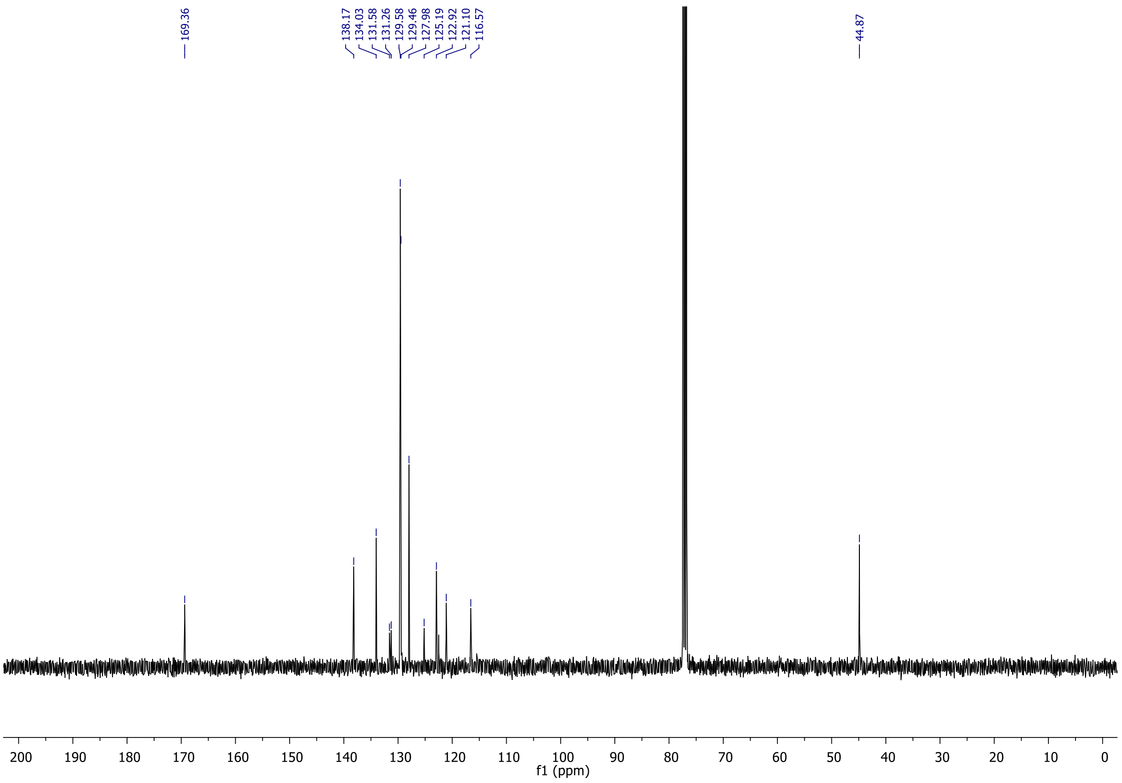

_N_-(3,5-bis(Trifluoromethyl)phenyl)-2-phenylacetamide 12

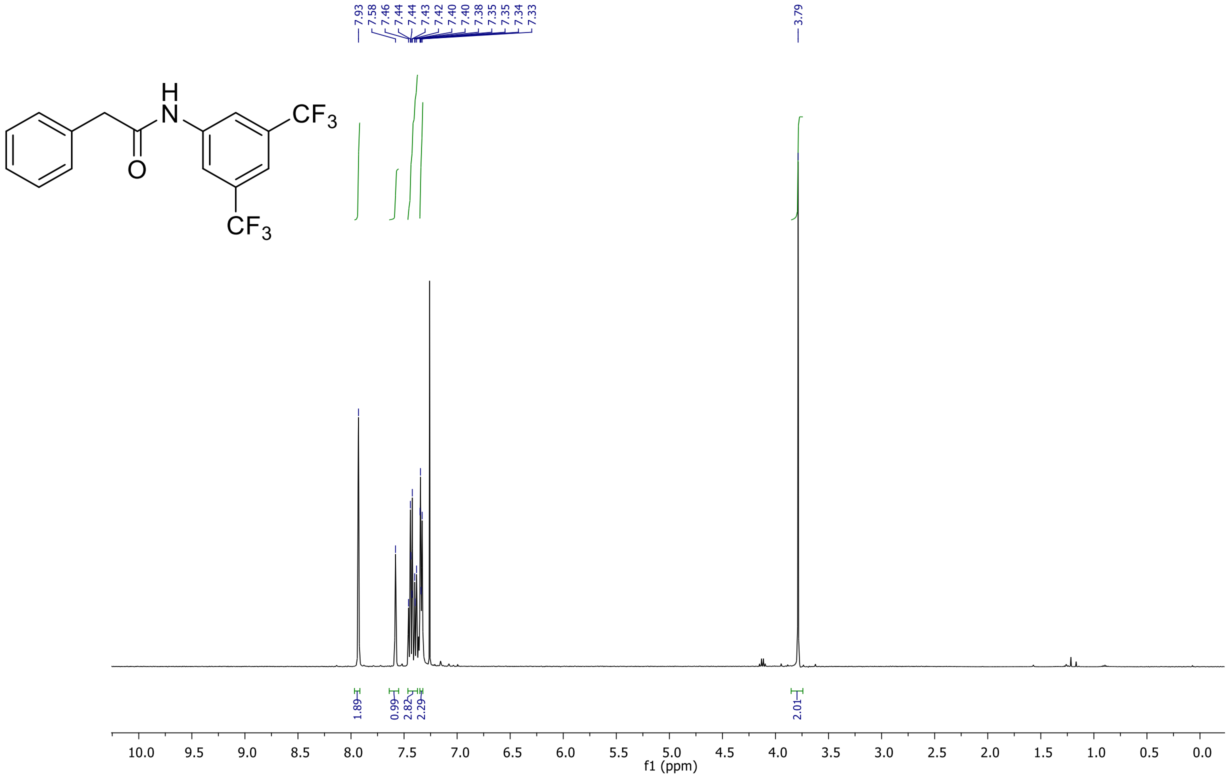

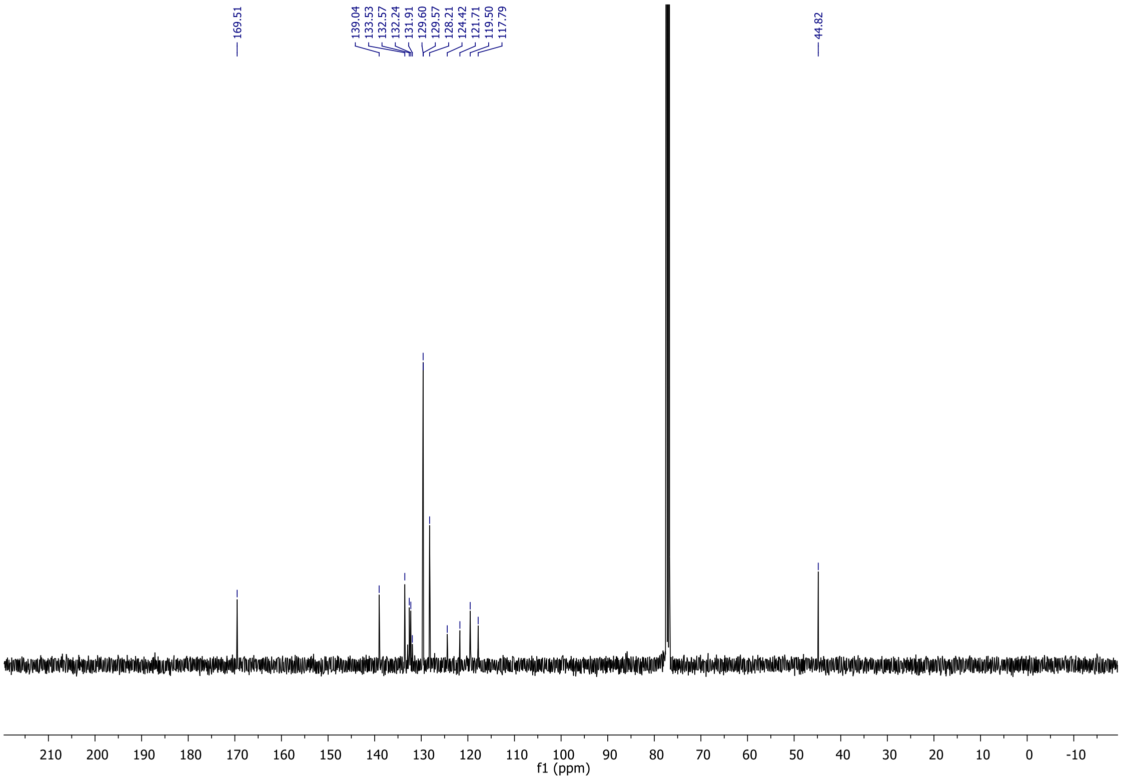

N-(3,5-Dichlorophenyl)picolinamide 13

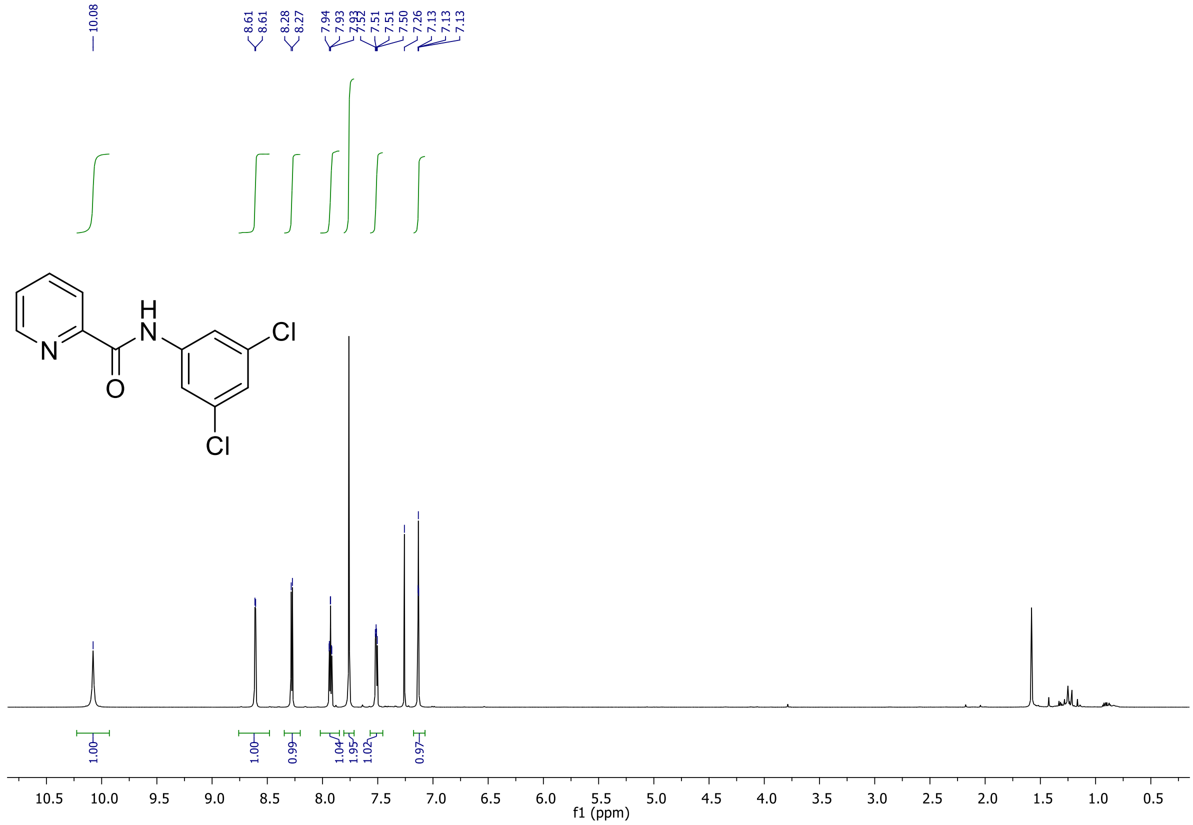

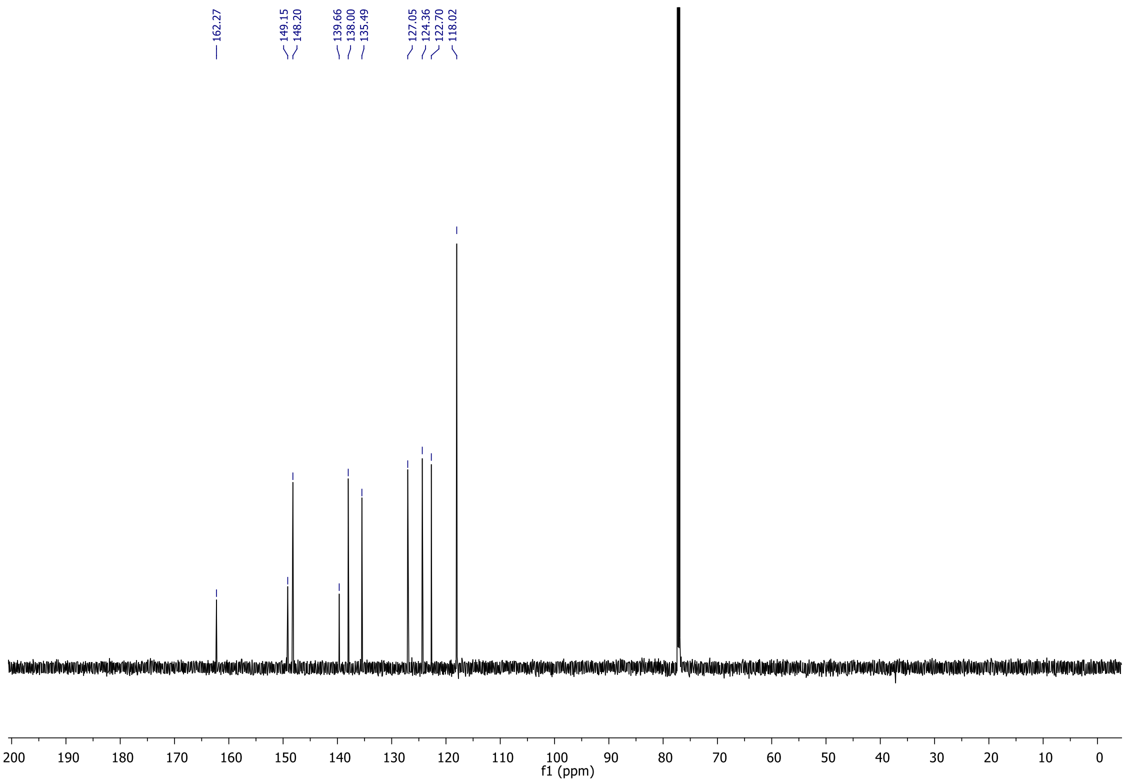

2-Phenyl-*N*-(pyridin-2-yl)acetamide 14

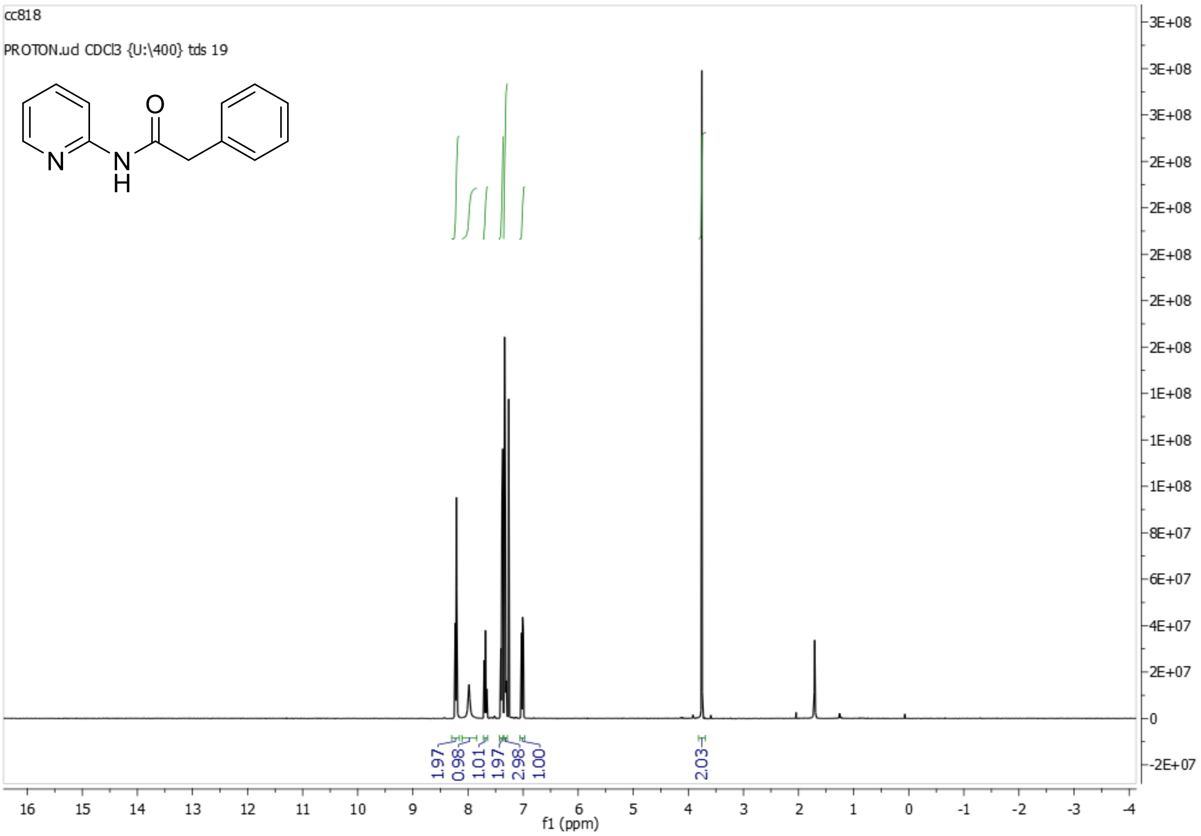

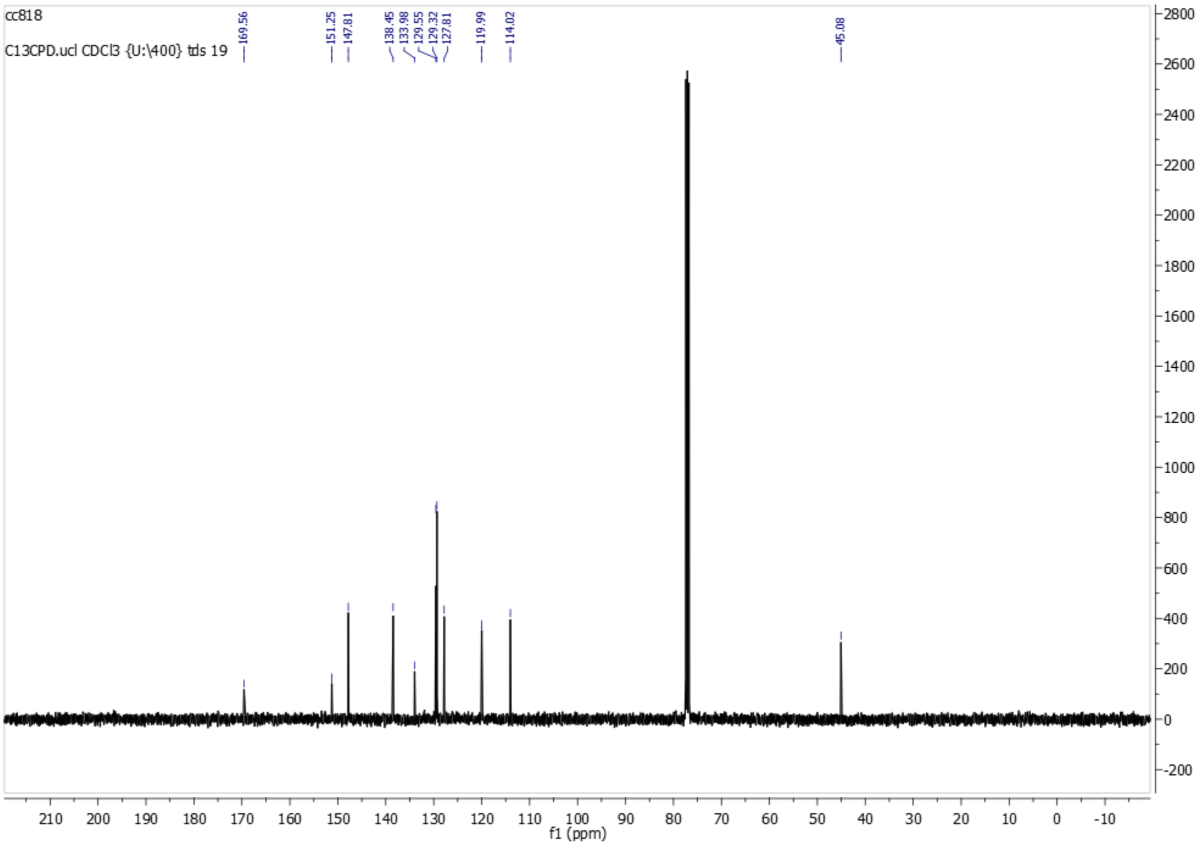

2-Phenyl-*N*-(pyridin-3-yl)acetamide 15

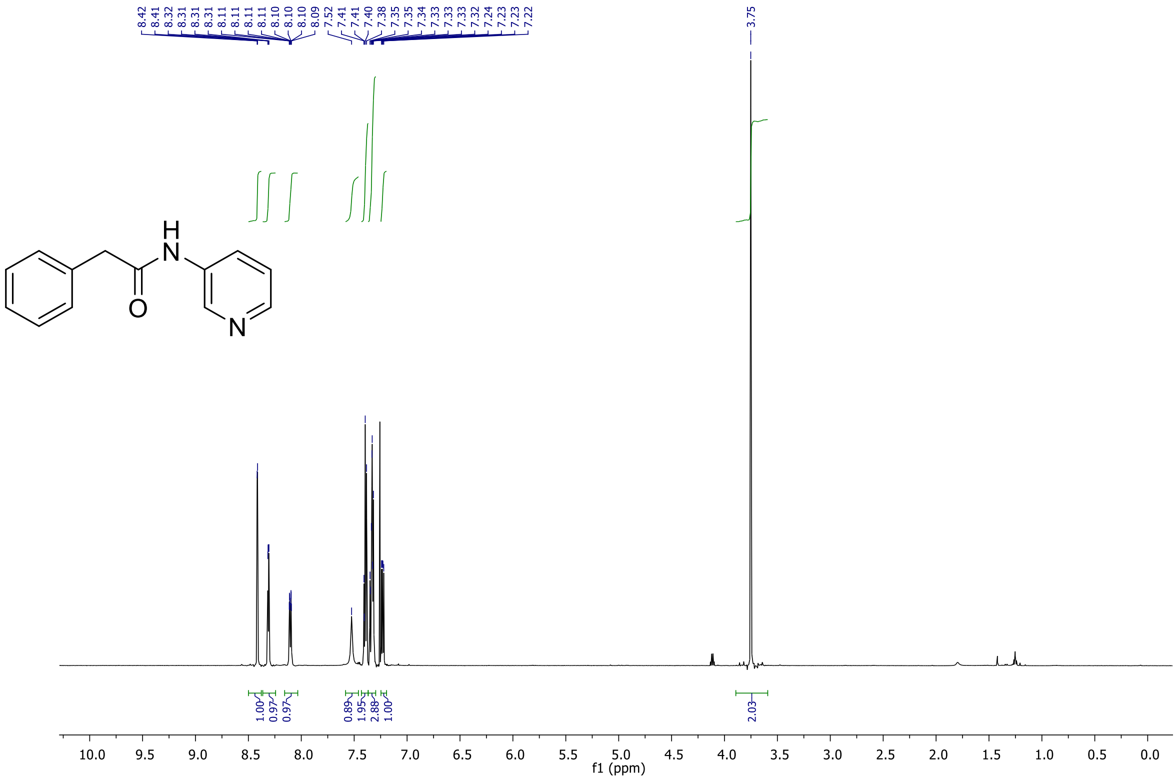

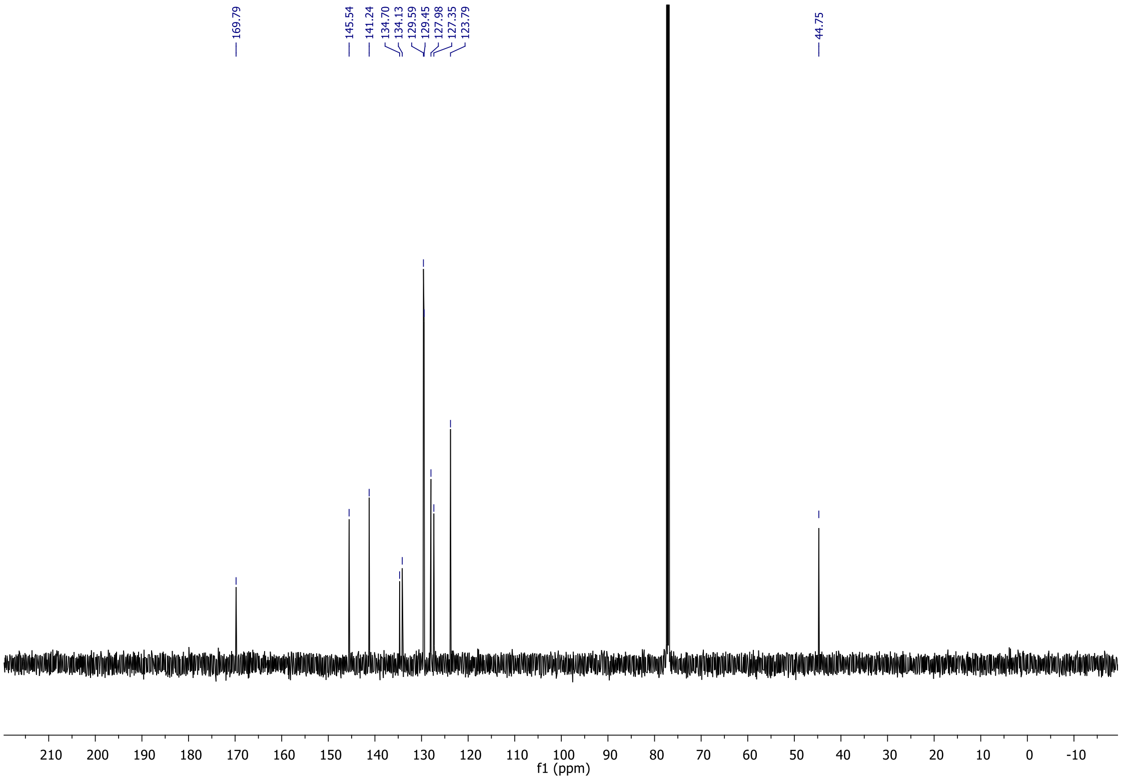

N-(4-Fluorobenzyl)-2,2-diphenylacetamide 16

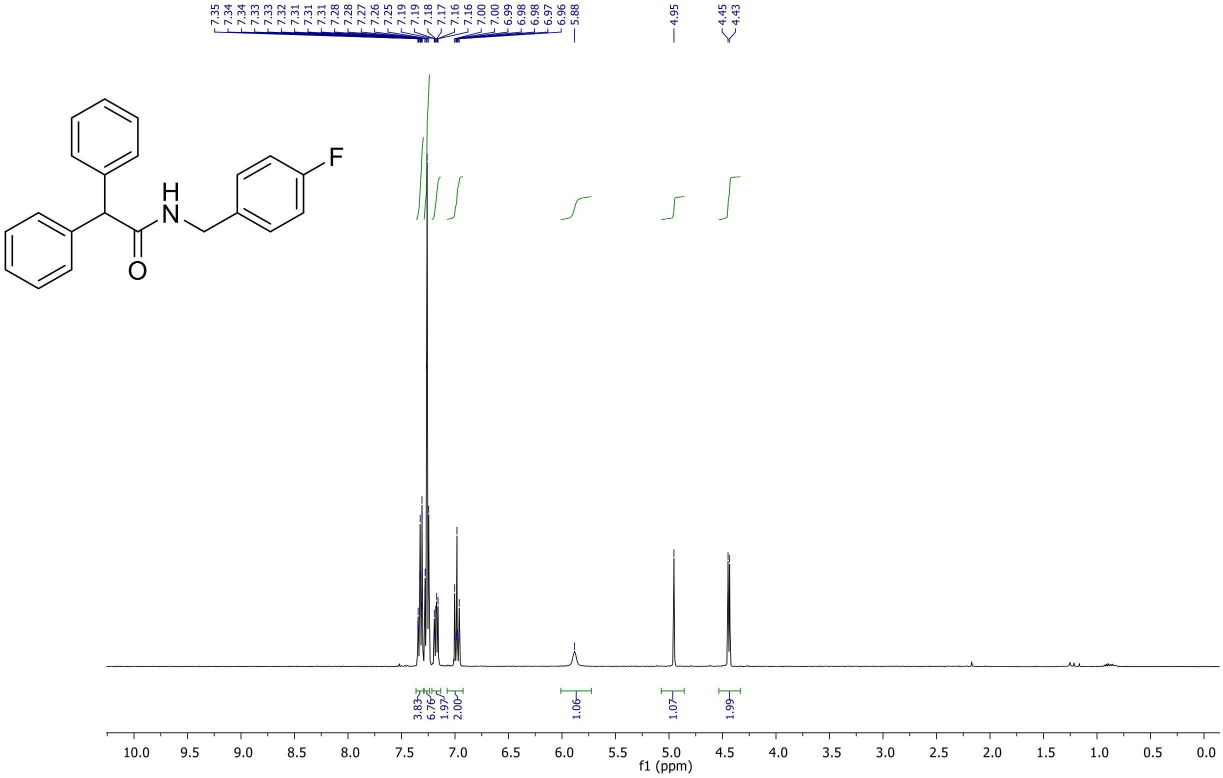

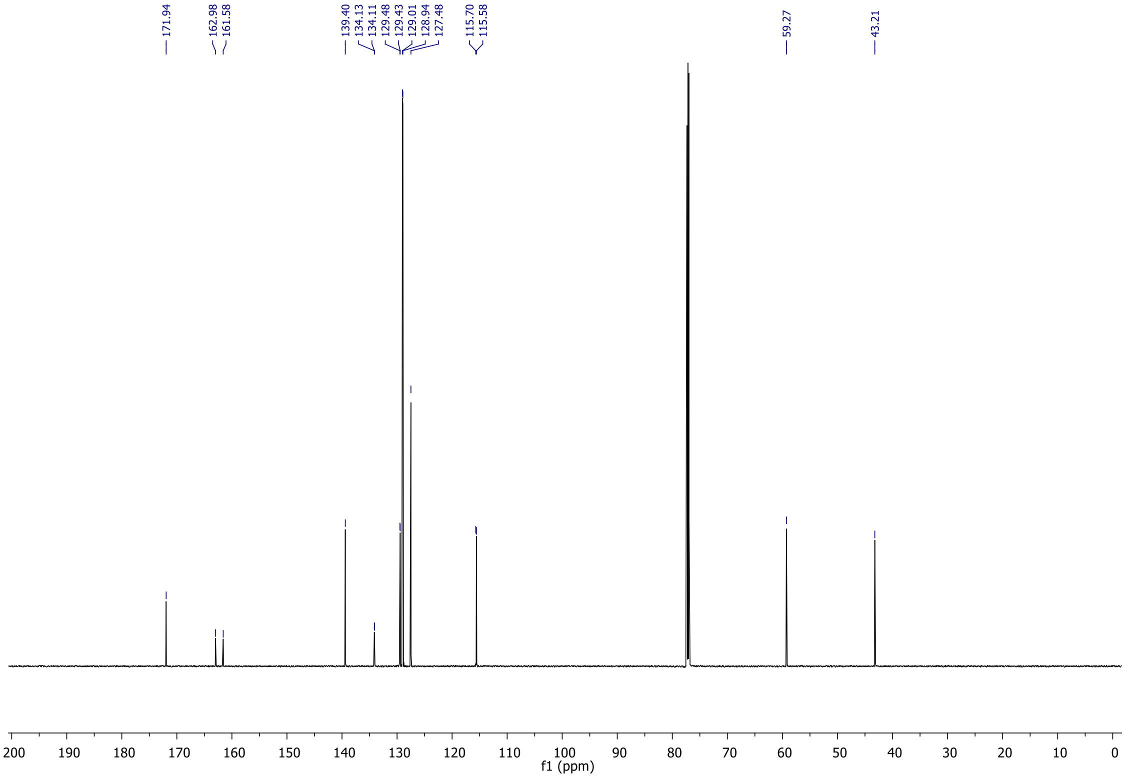

1-(Piperidin-1-yl)pentan-1-one 17

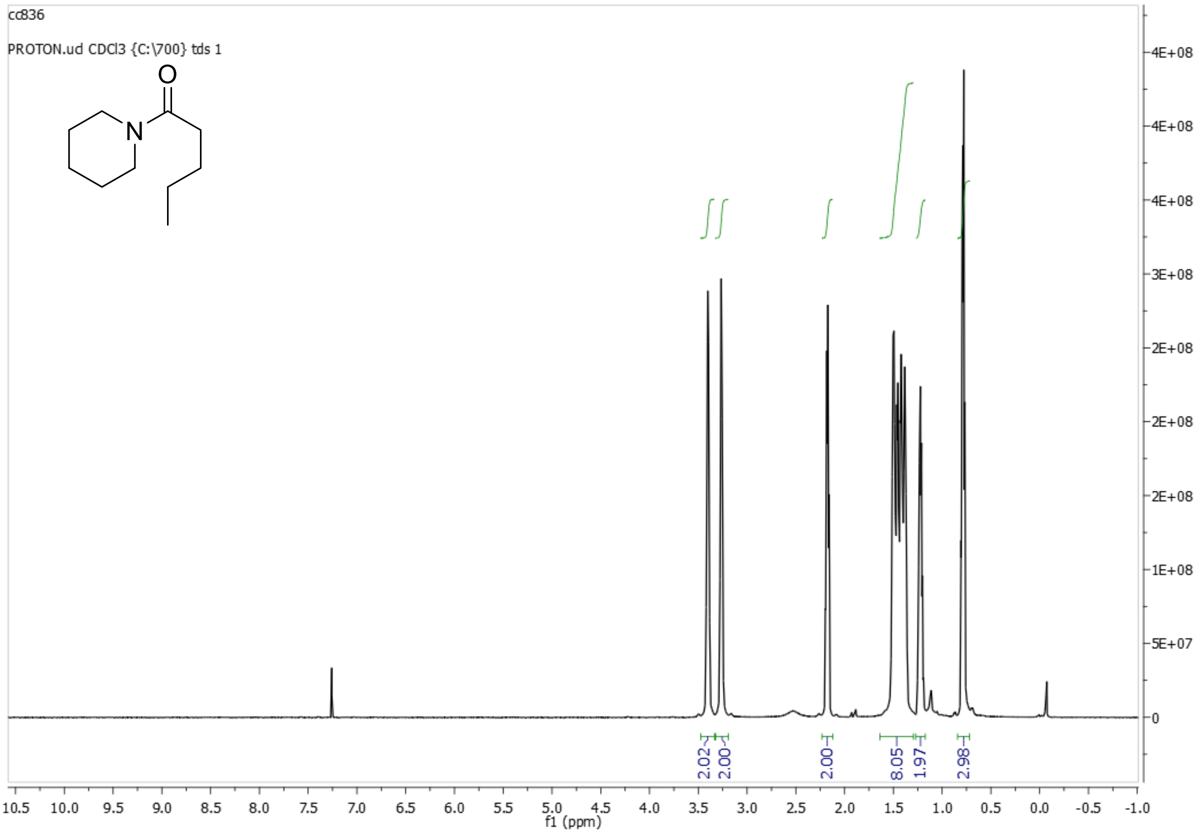

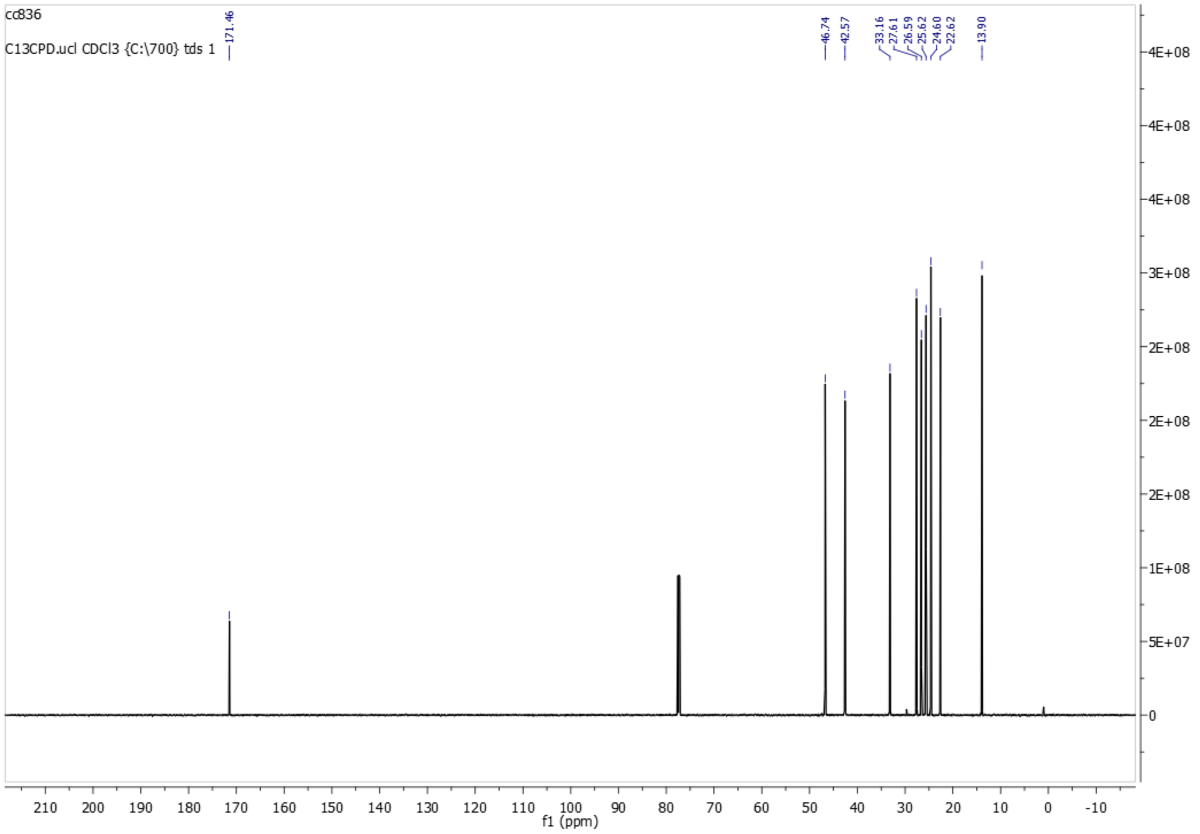

2-Phenyl-*N*-(1-phenylethyl)acetamide 18

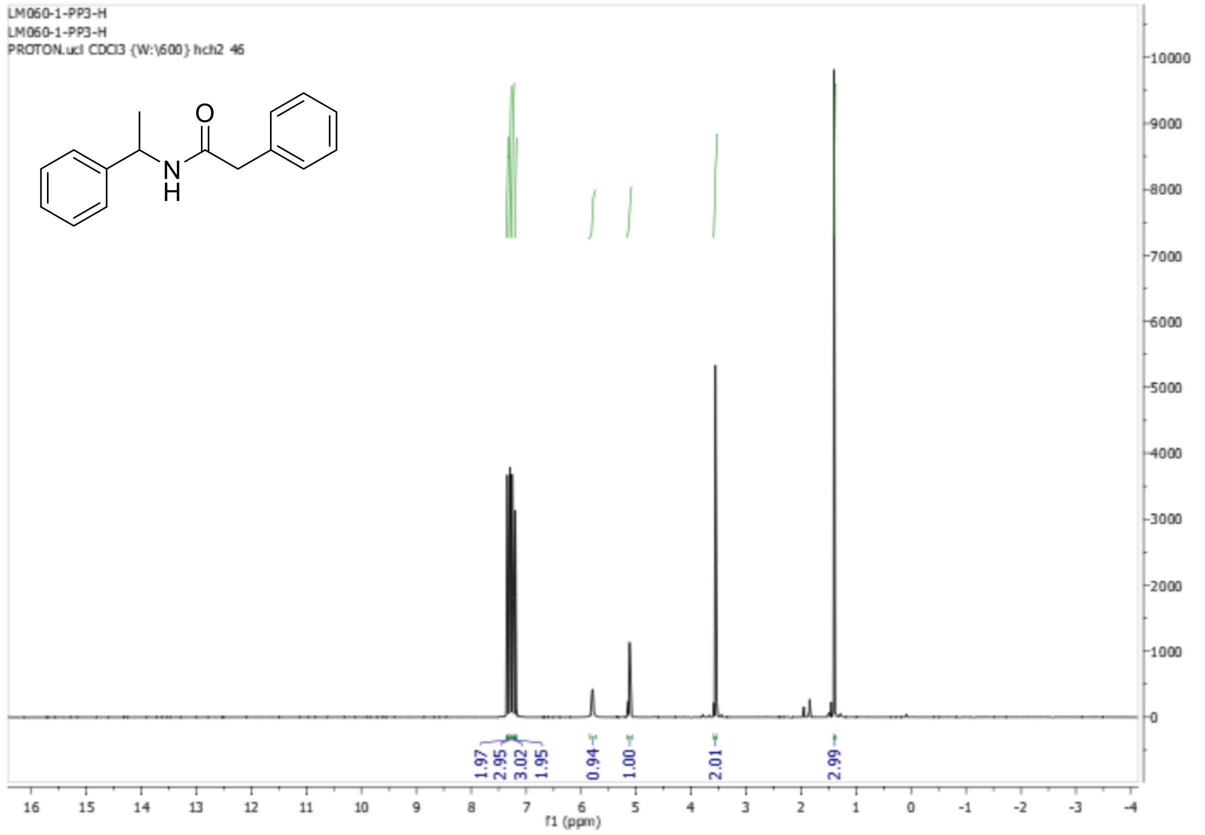

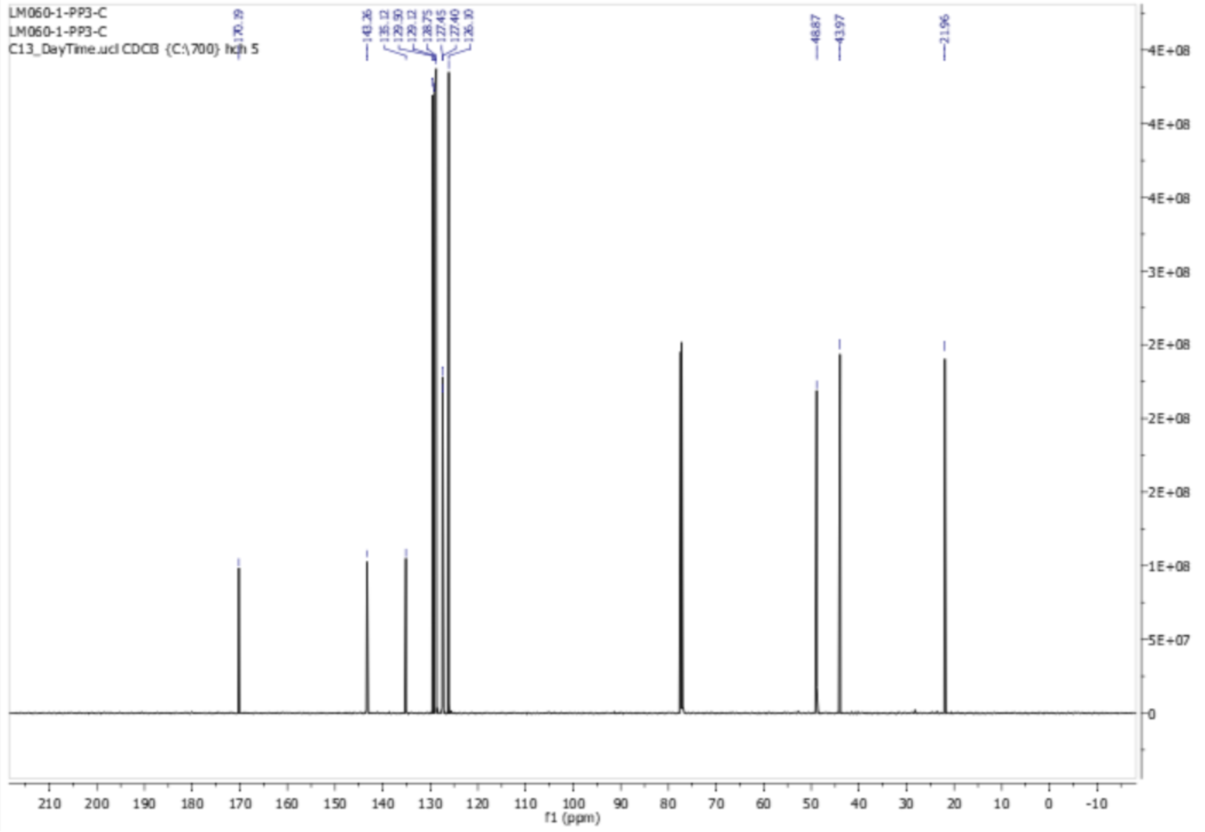

N-Benzyl-2-(2-chlorophenyl)-2-hydroxyacetamide 19

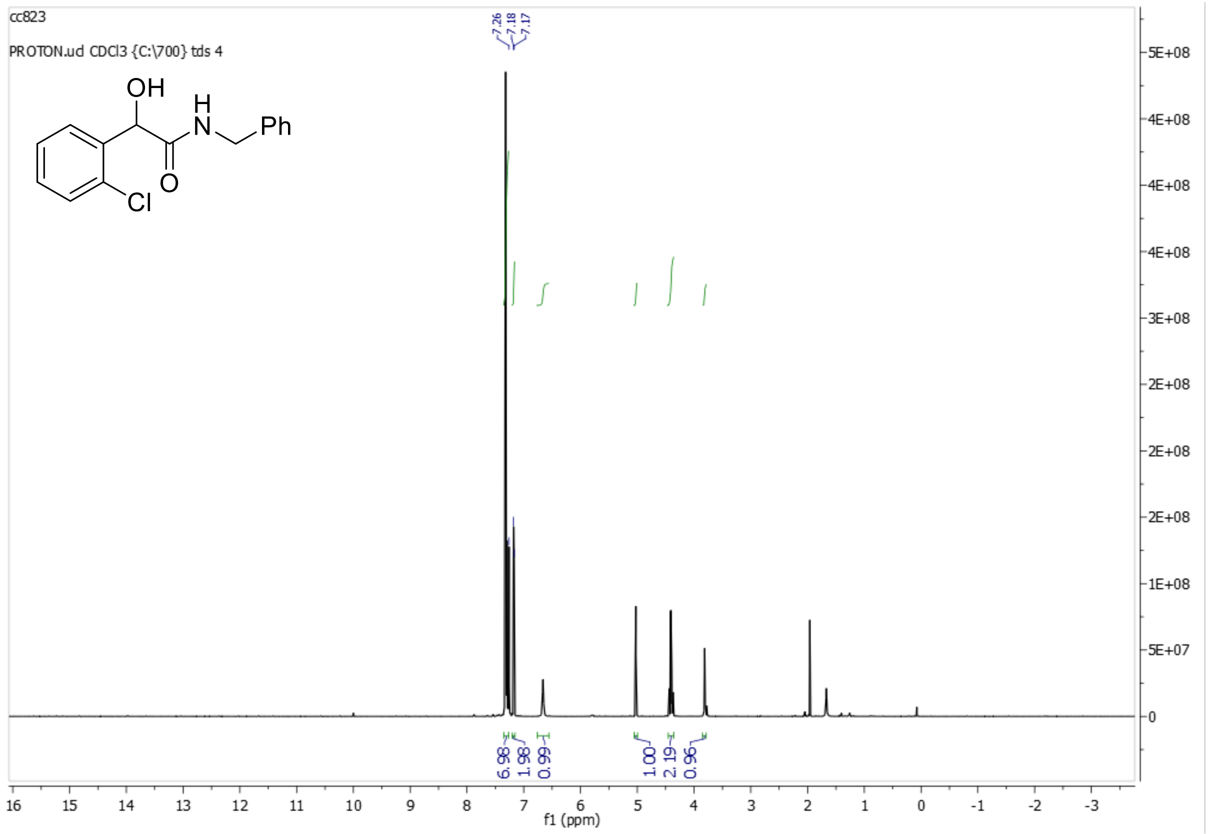

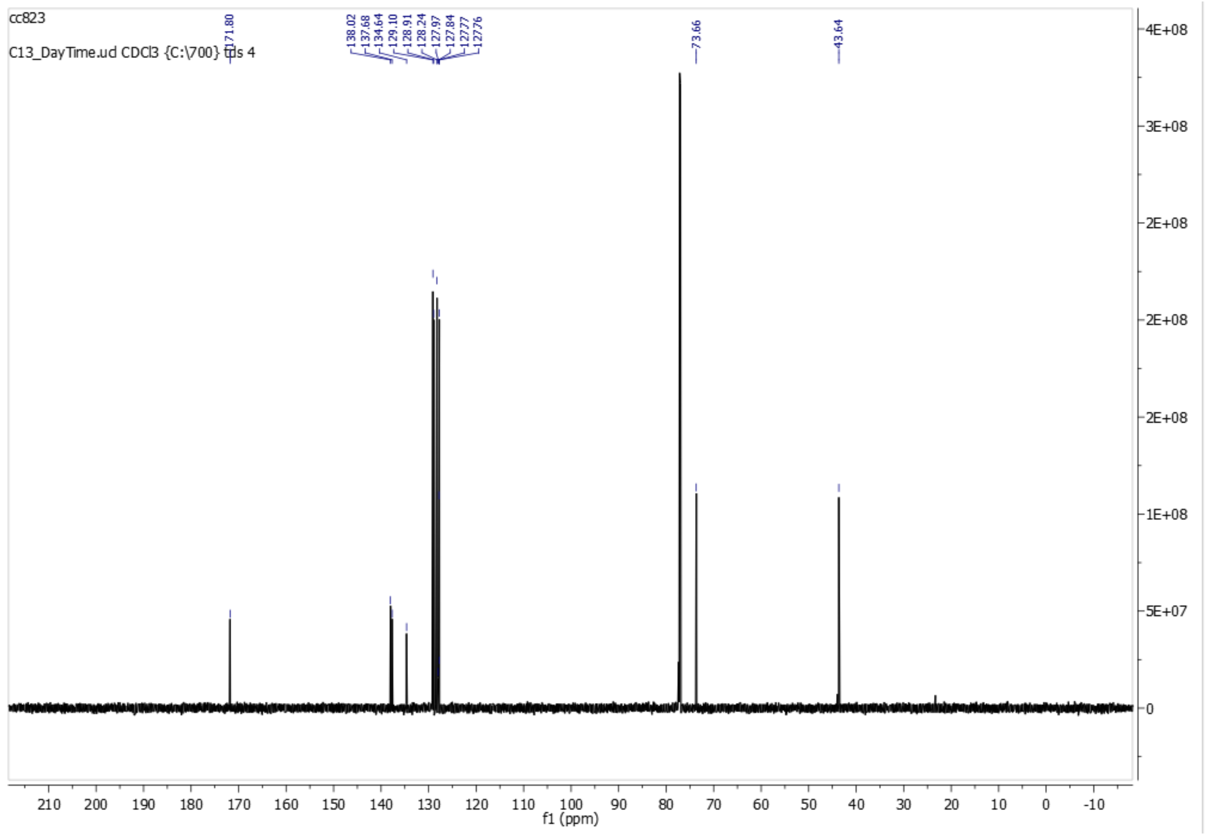

N-(3,4-Dimethoxyphenethyl)hexanamide 20

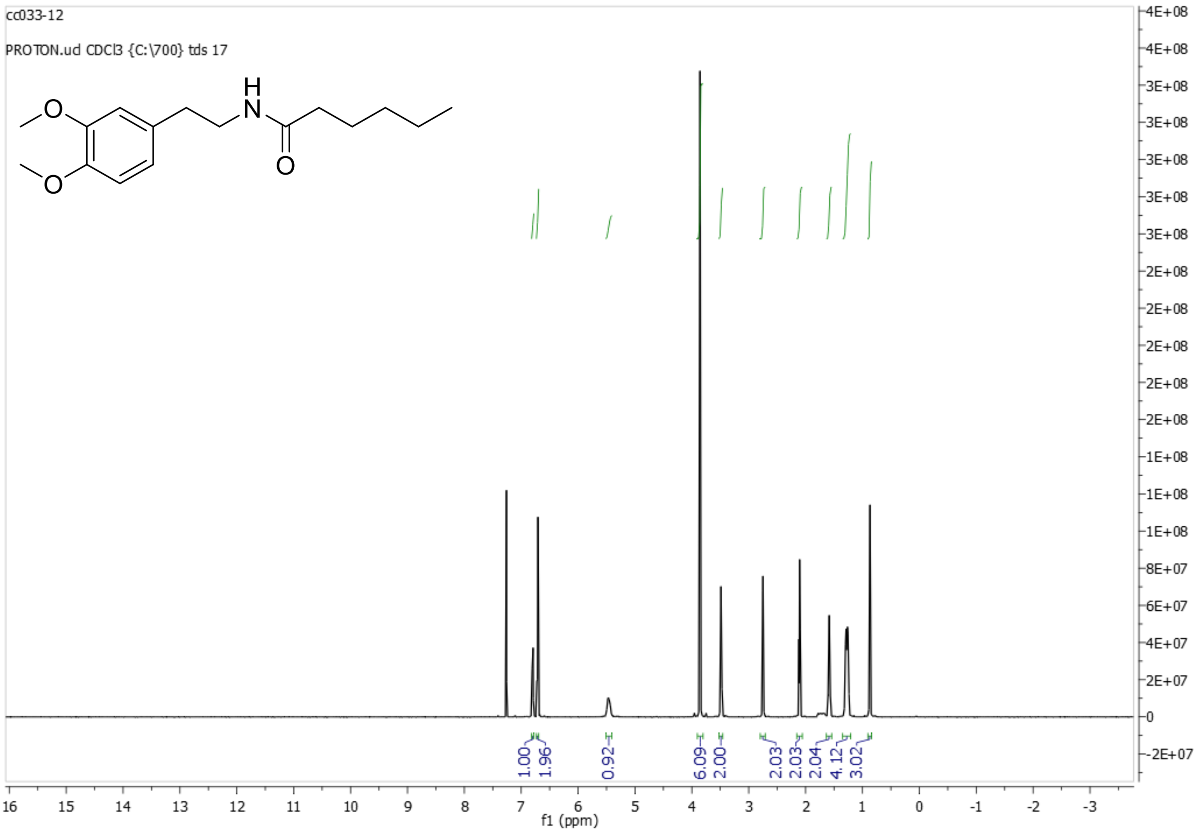

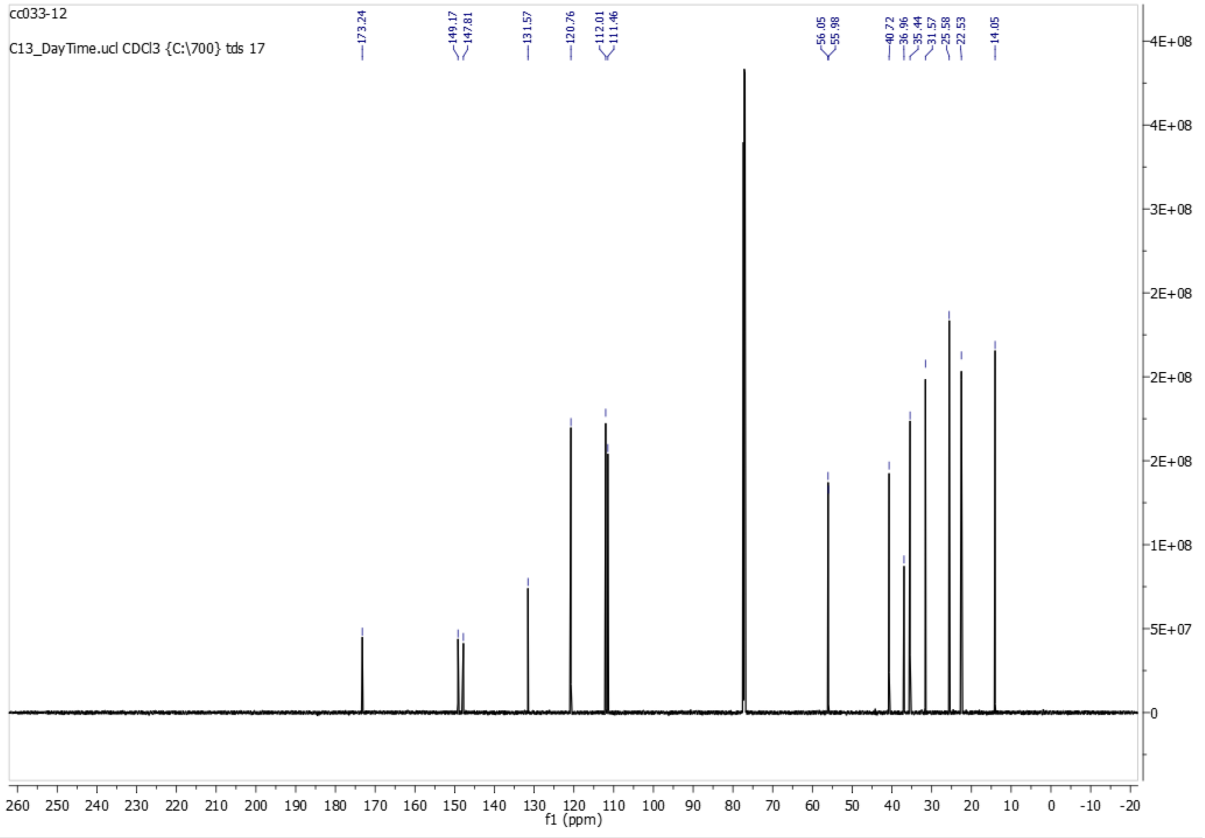

N-(2-Hydroxy-2-phenylethyl)hexanamide 21

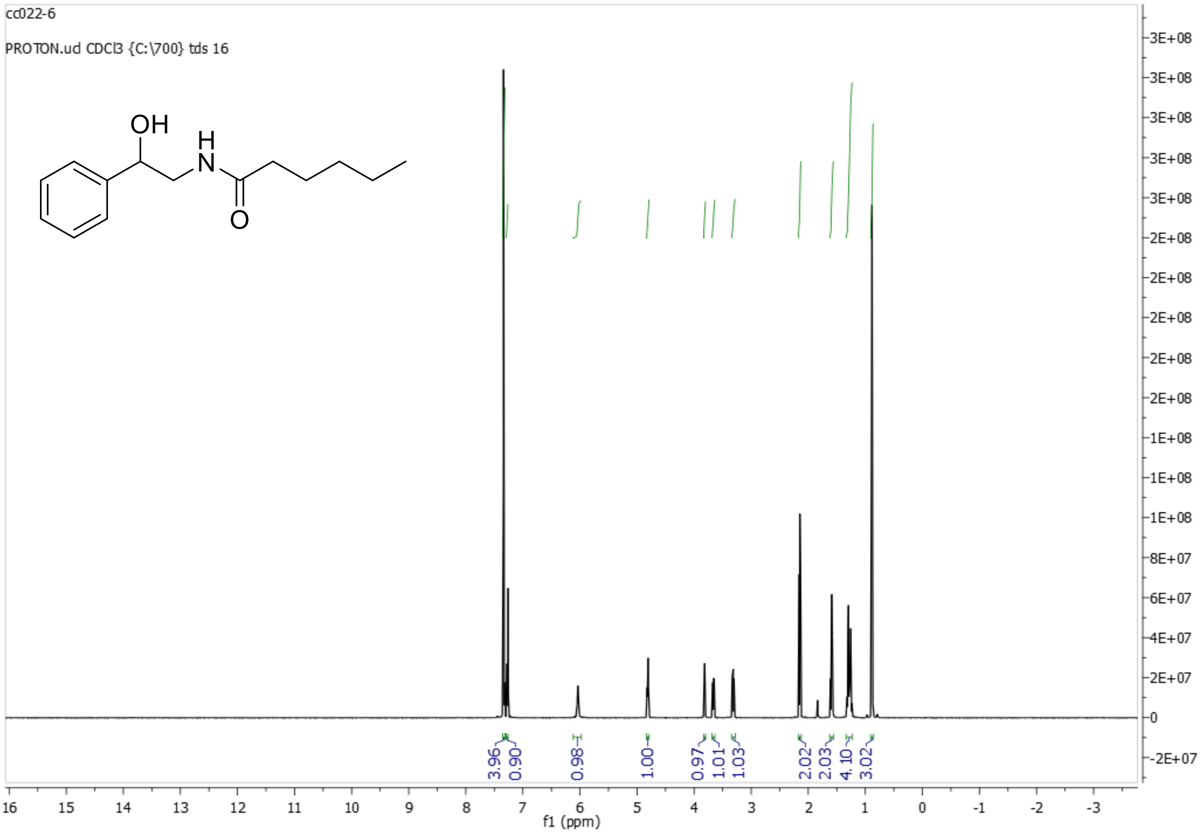

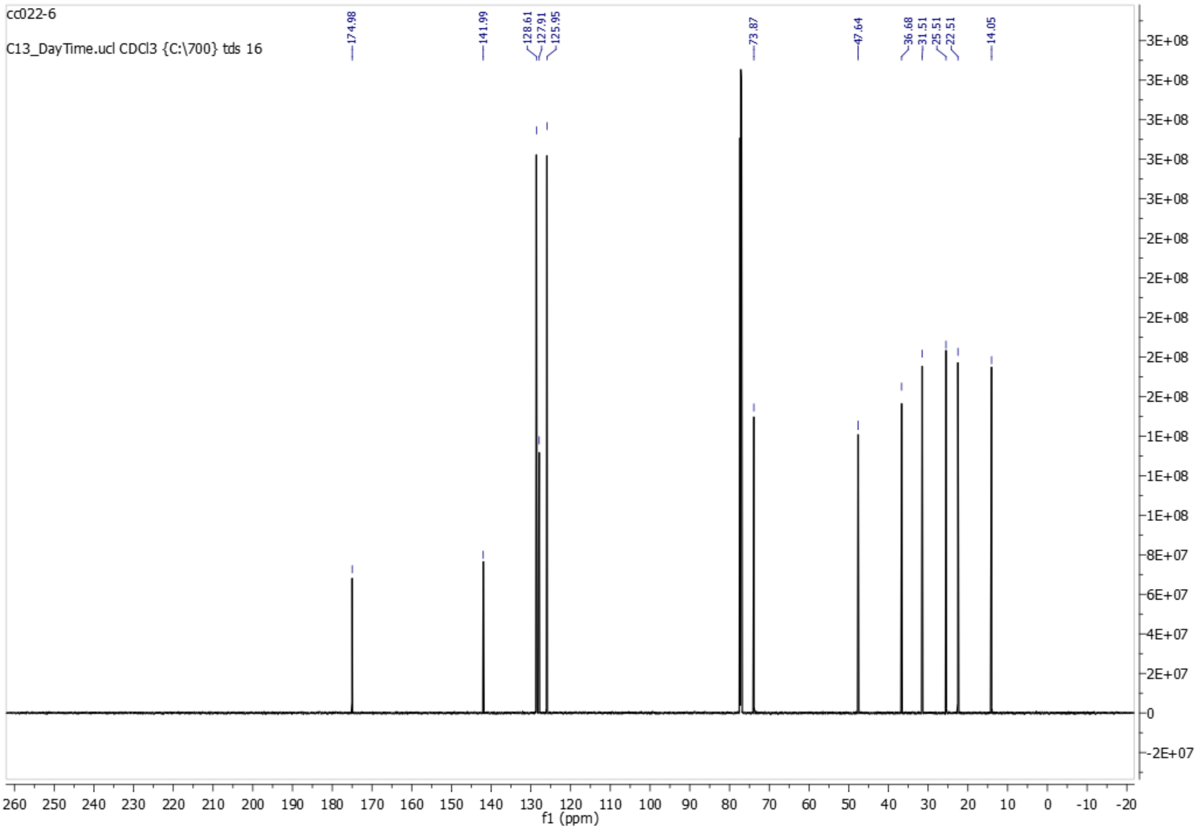

tert-Butyl (_tert_-butoxycarbonyl)-D-alanyl-L-phenylalaninate 22

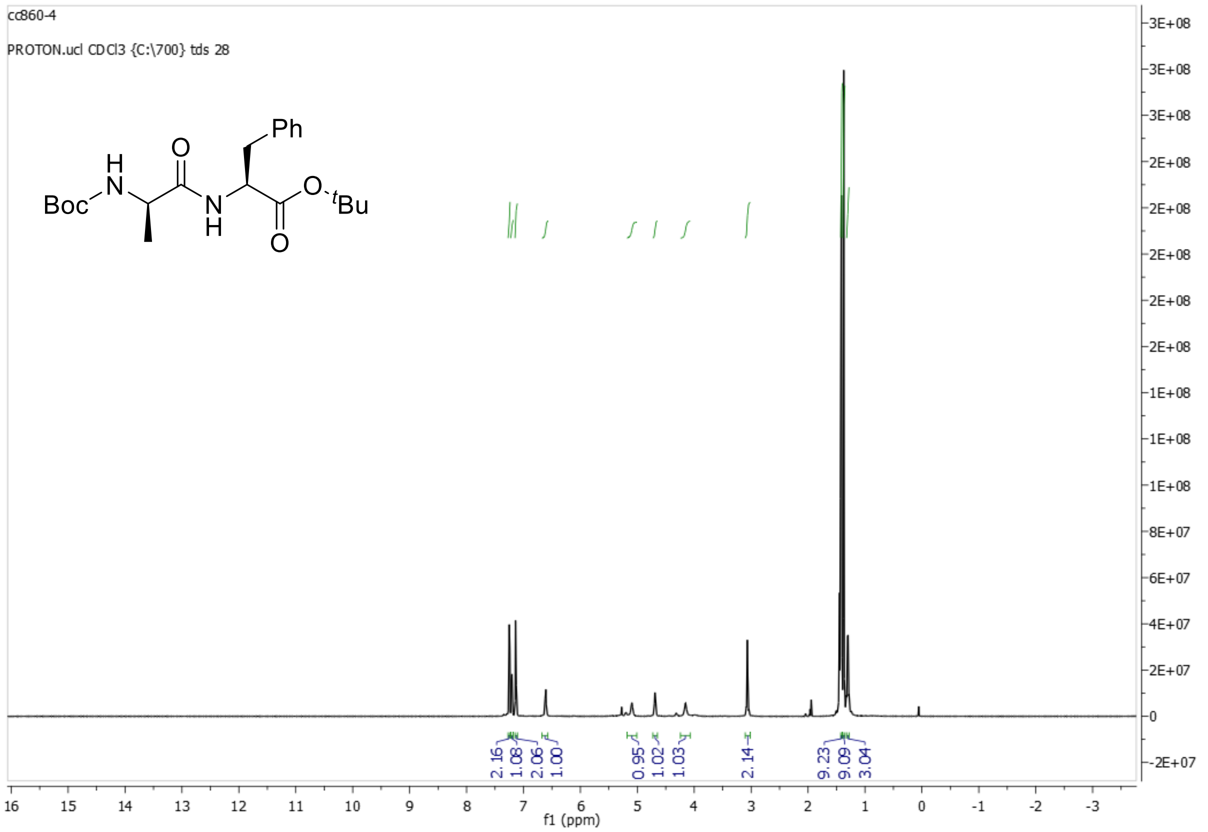

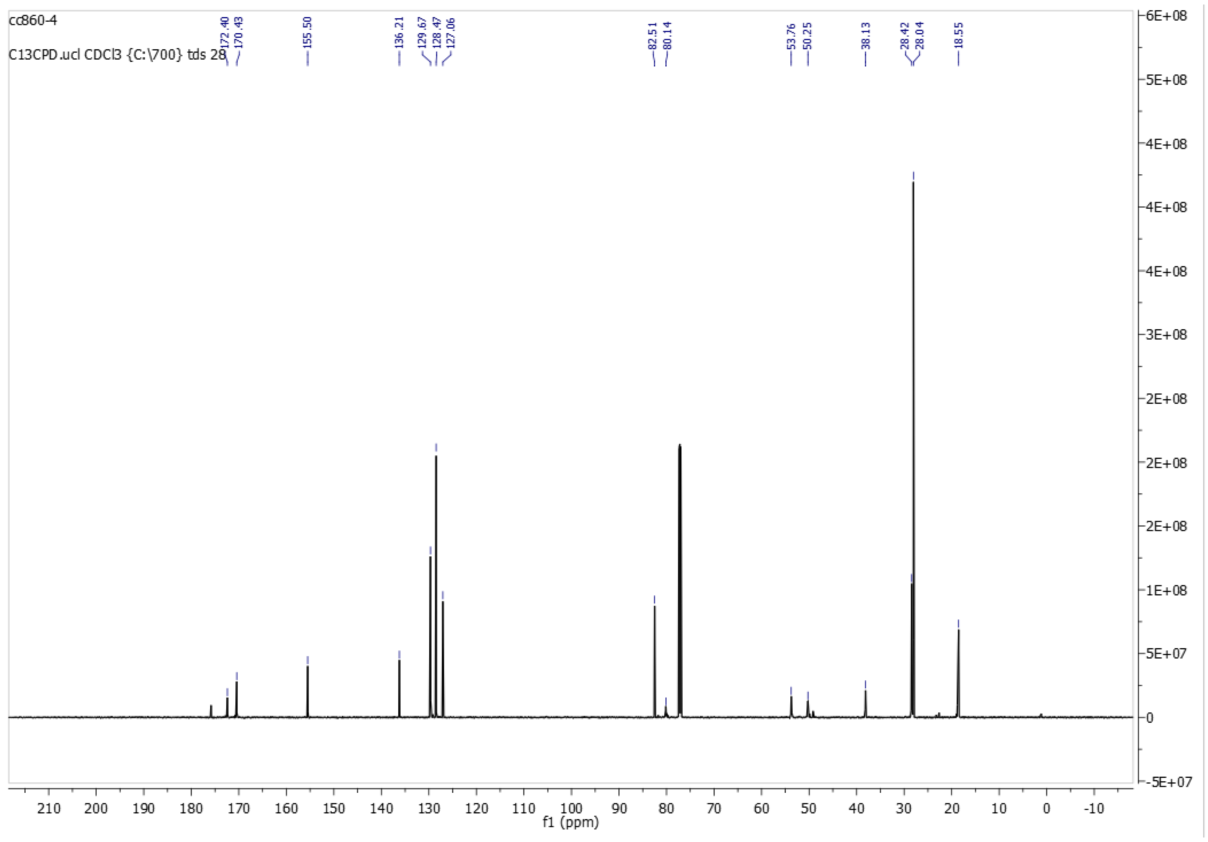

tert-Butyl (_tert_-butoxycarbonyl)-L-alanyl-L-phenylalaninate 23

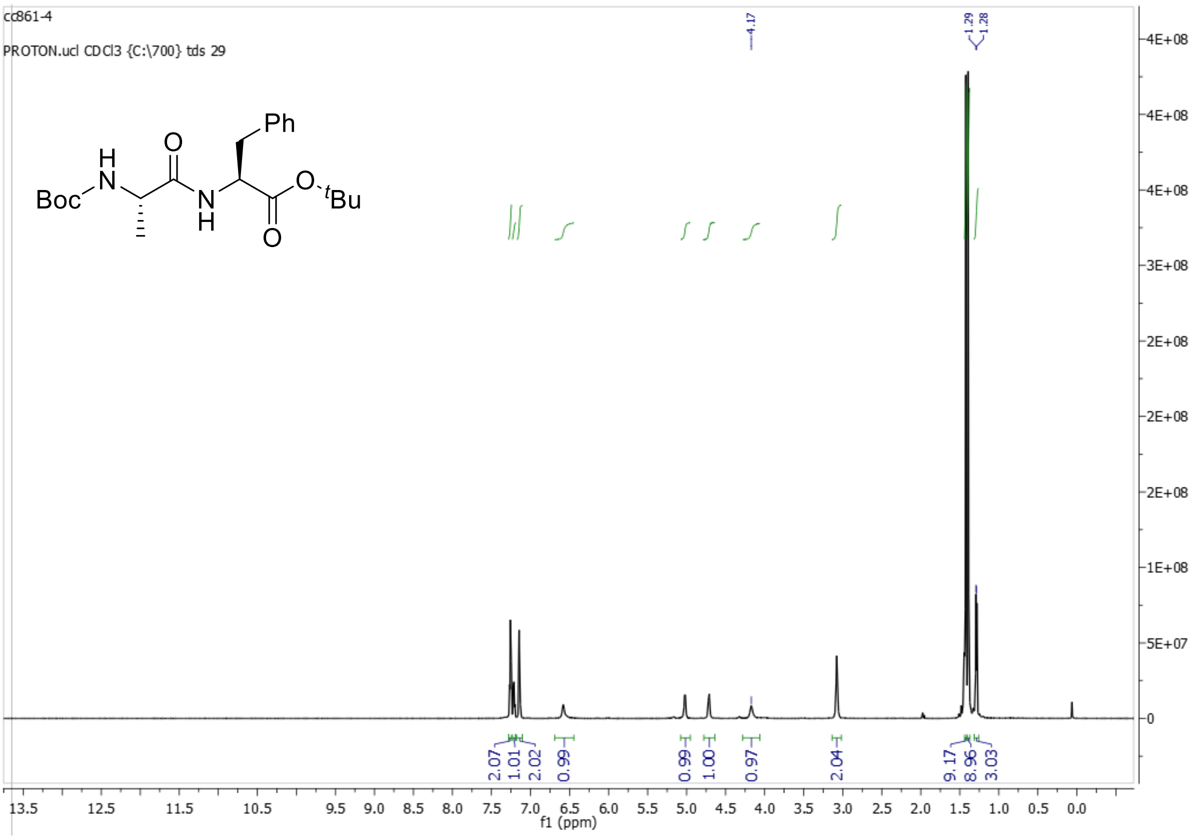

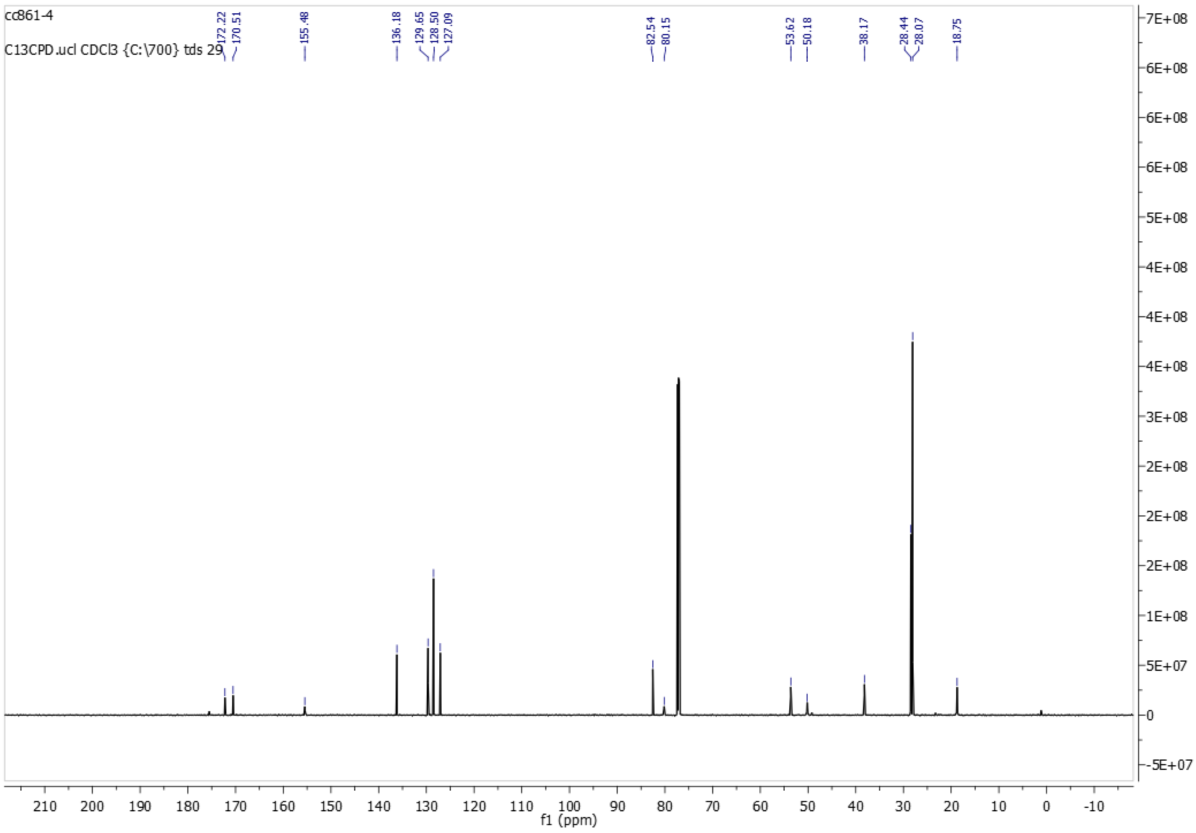

Superimposed 1H NMR spectra of dipeptides 22 and 23

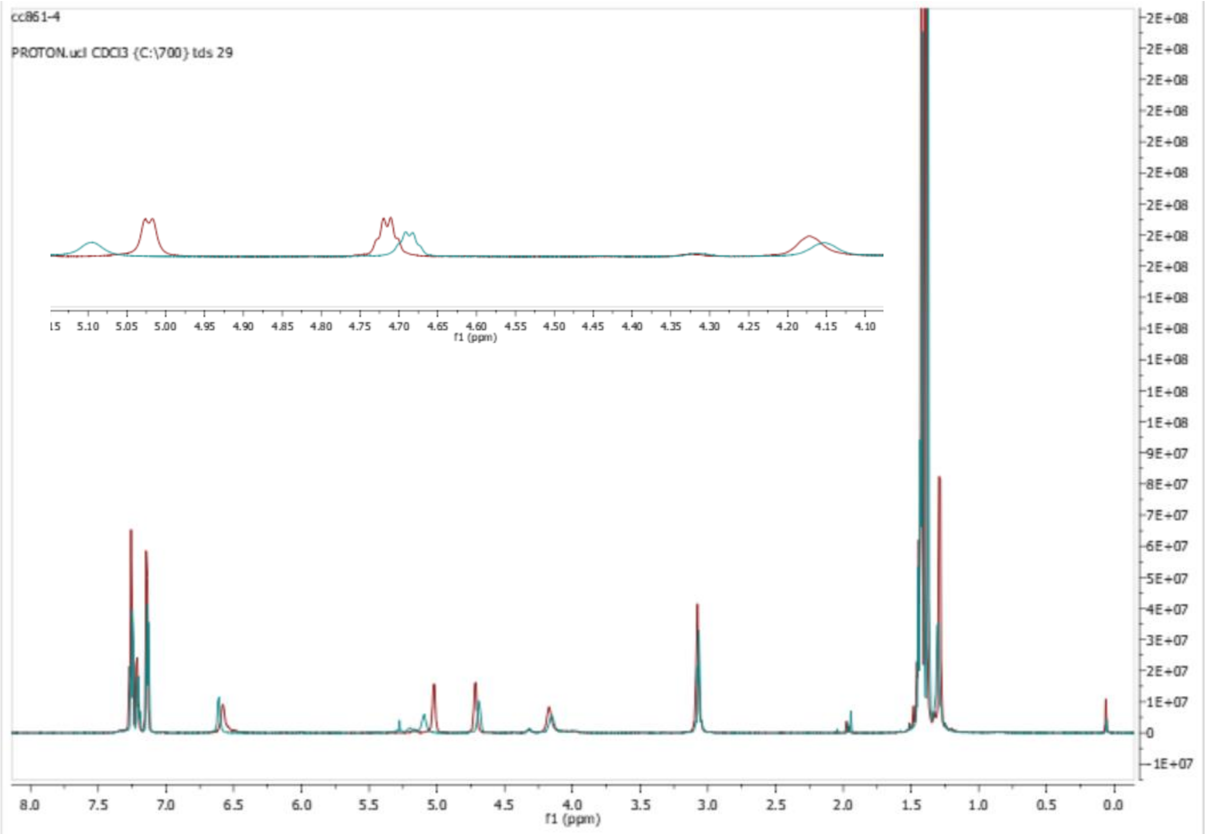

1-Methyl-*N*-((1R,3r,5S)-9-methyl-9-azabicyclo[3.3.1]nonan-3-yl)-1H-indazole-3-carboxamide 24

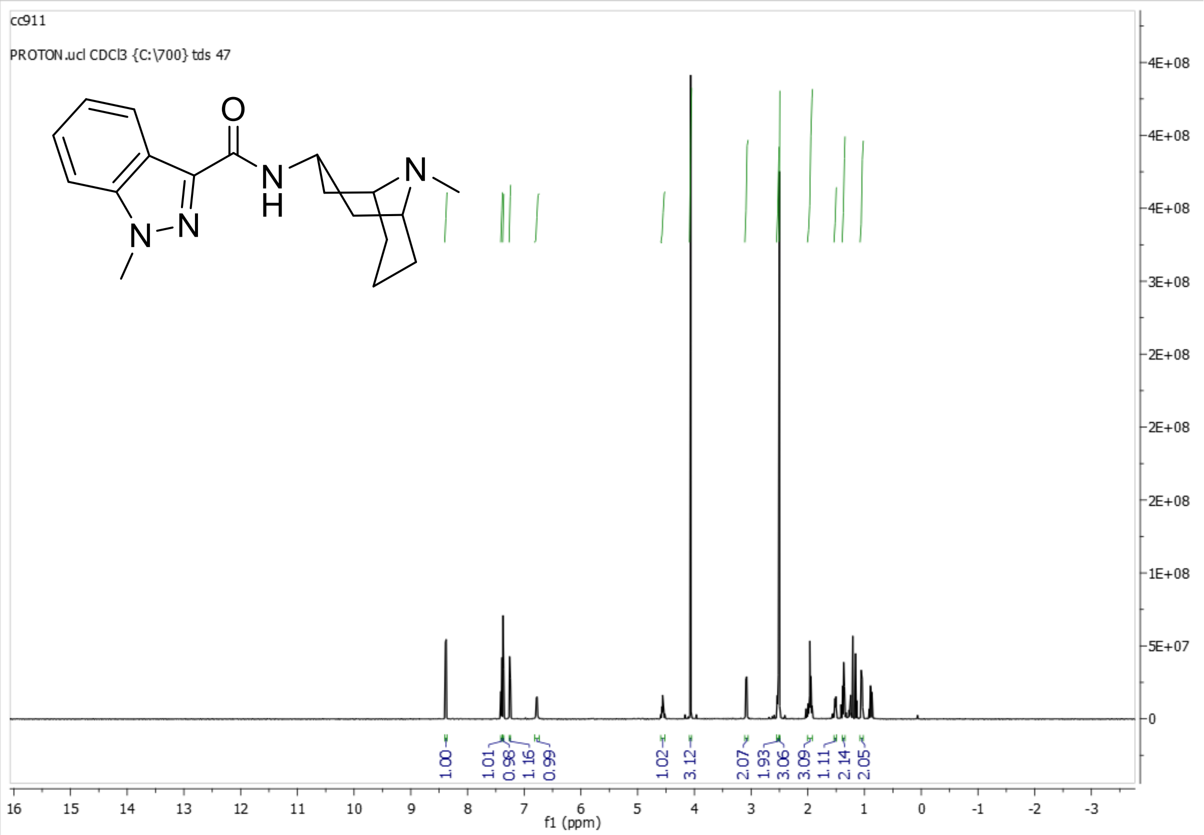

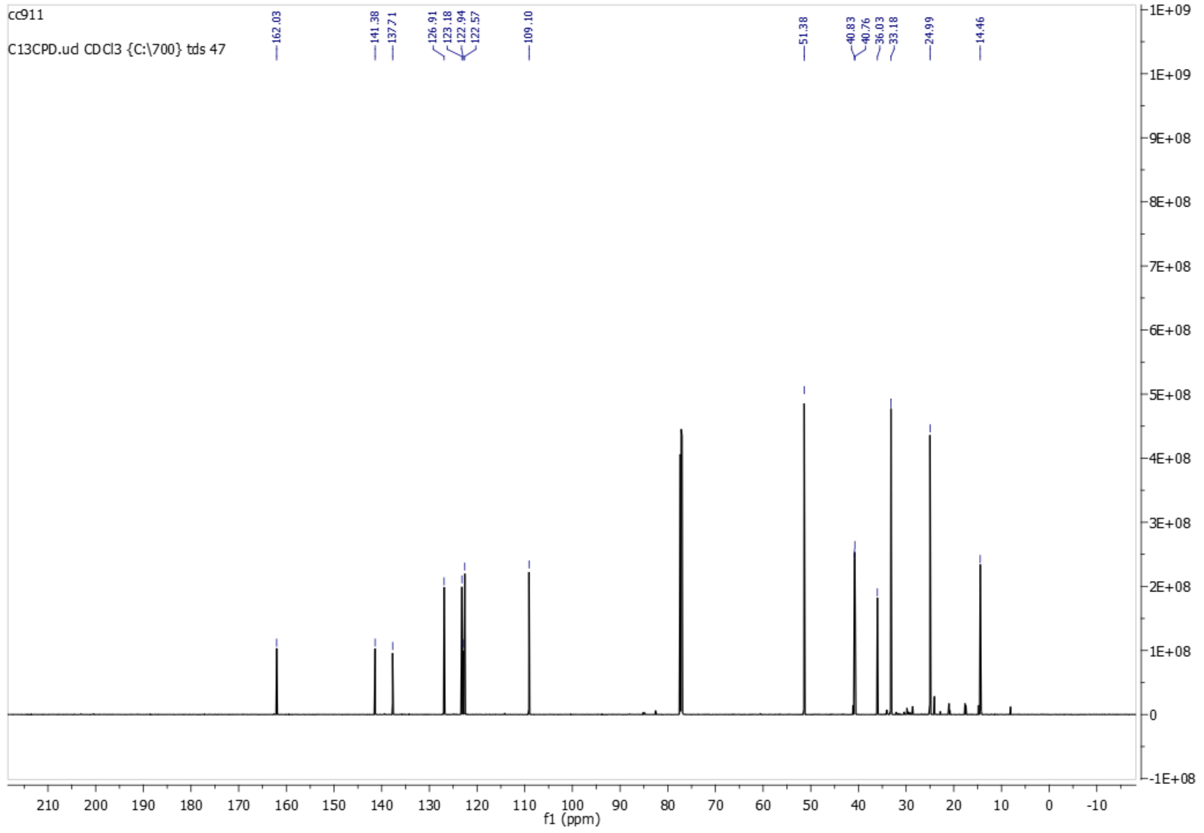

Supplement: Supplementary file 1 [file OB-017-C9OB01012B-s001.pdf]
